# Bulbillosins A - E, azaphilones from *Tengochaetabulbillosa* sp. nov. (*Chaetomiaceae*), a root endophyte of the Chinese medicinal plant *Astertataricus*

**DOI:** 10.3897/imafungus.16.141036

**Published:** 2025-02-17

**Authors:** Diana Astrid Barrera-Adame, Yasmina Marin-Felix, Ana Kristin Wegener, Michael Lalk, Marc Stadler, Timo H. J. Niedermeyer

**Affiliations:** 1 Department of Pharmaceutical Biology, Institute of Pharmacy, Freie Universität Berlin, Königin-Luise-Str. 2+4, 14195 Berlin, Germany Freie Universität Berlin Berlin Germany; 2 Department Microbial Drugs, Helmholtz Centre for Infection Research, Inhoffenstraße 7, 38124 Braunschweig, Germany Helmholtz Centre for Infection Research Braunschweig Germany; 3 Institute of Microbiology, Technische Universität Braunschweig, Spielmannstraße 7, 38106 Braunschweig, Germany Technische Universität Braunschweig Braunschweig Germany; 4 Department of Pharmaceutical Biology/Pharmacognosy, Institute of Pharmacy, Martin Luther University Halle-Wittenberg, 06120 Halle (Saale), Germany Martin Luther University Halle-Wittenberg Halle (Saale) Germany; 5 Current affiliation: Winckelmann Apotheke, 39576 Stendal, Germany Winckelmann Apotheke Stendal Germany; 6 Department of Cellular Biochemistry and Metabolomics, Institute of Biochemistry, University of Greifswald, Felix-Hausdorff-Strasse 4, 17487 Greifswald, Germany University of Greifswald Greifswald Germany

**Keywords:** *Ascomycota*, *Asteraceae*, cytotoxicity, endophyte, mass spectrometry imaging, *Sordariales*

## Abstract

*Astertataricus* is a plant used in Traditional Chinese Medicine. From its roots, we isolated four endophytic fungi strains. After mass spectrometry analysis and subsequent molecular networking and dereplication, one of the strain’s extracts showed a cluster of yet undescribed natural products. Additionally, the extract was found to be lethal for the nematode *Caenorhabditiselegans* and cytotoxic against eukaryotic cell lines. The fungal strain was characterized by morphological and molecular studies, allowing its description as a new species in the genus Tengochaeta (Chaetomiaceae), *Tengochaetabulbillosa*. After cultivation and extraction of the strain, the major secondary metabolites were isolated. Structure elucidation based on nuclear magnetic resonance spectroscopy and high-resolution tandem mass spectrometry revealed these compounds to be five new azaphilones. Additionally, the localization of these azaphilones in the host plant was studied by mass spectrometry imaging of different plant tissues, revealing that they were mainly localized in the aerial parts of the plant. The main compound, bulbillosin A, was evaluated for its activity against sixty cancer cell lines, revealing a differential cytotoxicity profile.

## Introduction

*Astertataricus* (*Asteraceae*) is a plant used in Traditional Chinese Medicine (Li et al. 2022). It has been known for the production of astins, cyclic peptides with antitumor activity (Morita et al. 1993, 1995; Xu et al. 2013). Recently, however, it has been revealed that the astins are actually produced by an endophytic fungus, *Cyanodermellaasteris* (*Lecanoromycetes*, *Ascomycota*), isolated from the inflorescence axis of the plant (Jahn et al. 2017; Schafhauser et al. 2019).

Endophytes are microorganisms that colonize living healthy plants in a symbiotic or mutualistic relationship, without causing any apparent disease (Owen and Hundley 2004). There are many reports about specialized metabolites produced by endophytes (Venugopalan and Srivastava 2015; Caruso et al. 2020). However, the spatial distribution of these metabolites *in situ*, in the host plants, remains often unknown. New techniques like imaging mass spectrometry could help to understand better the role that endophyte metabolites can play in fungus-plant interactions (Ho et al. 2017).

Endophytes are also an important source of novel secondary metabolites with potential pharmaceutical applications. Activities that have been described for compounds isolated from endophytes include anticancer or immunosuppressive activity (Porras-Alfaro and Bayman 2011; Caruso et al. 2020). Root endophytes have been less studied compared to mycorrhizae, belittling the role that they play as a fundamental component of ecosystems (Porras-Alfaro and Bayman 2011). To minimize the risk of re-discovering known compounds and the respective costs due to the loss of time and resources (Marmann et al. 2014), efficient dereplication strategies are essential. An important computational tool in this regard is GNPS (Wang et al. 2016; Aron et al. 2020), allowing the rapid analytical screening and dereplication of the metabolites present in crude extracts. Combined with the software Cytoscape (Shannon et al. 2003) to visualize the results, it facilitates the identification of structural similarities and differences of compounds contained in extracts, enabling rapid prioritization for subsequent natural product chemistry workflows.

Azaphilones are specialized metabolites known to be produced by a wide range of fungal genera like *Chaetomium*, *Aspergillus*, *Penicillium*, *Pestalotiopsis*, *Diaporthe*, *Talaromyces*, *Monascus*, *Epicoccum*, and *Hypoxylon* (Chen et al. 2020; Yang et al. 2021; Huang et al. 2022). Structurally, they are characterized by an oxygenated pyrano-quinone bicyclic core, known as isochromene (Chen et al. 2020). Recent research demonstrated that azaphilone derivatives possess various biological activities, including the inhibition of enzymes, antimicrobial, cytotoxic, antiviral, antileishmanial, and antimalarial activities (Chen et al. 2020).

Azaphilone biosynthesis uses polyketide and fatty acid synthesis pathways to assemble the base scaffold. Azaphilones are classified into 13 structural types (Chen et al. 2020), usually named according to the species from which they were first isolated. Thus, compounds that share e.g. the cohaerin core structure, but differ only in the lipophilic side chain, are given different names, such as minutellins, cohaerins, and longirostrerones that were isolated from *Jackrogersellaminutellum* (Kuhnert et al. 2017) and *Jackrogersellacohaerens* (Quang et al. 2006; Surup et al. 2013), both formerly known as *Annulohypoxylon* species (Wendt et al. 2018), and *Staphylotrichumlongicolle*, formerly known as *Chaetomiumlongirostre* (Panthama et al. 2011; Wang et al. 2019), respectively. Some azaphilones have become compounds of chemotaxonomic importance, e.g. cohaerin A and minutellins, which only occur in closely related species (Kuhnert et al. 2017, 2021).

In this manuscript, we describe the isolation and identification of *Tengochaetabulbillosa*, a new endophytic fungus from roots of *A.tataricus*, as well as the isolation, structure elucidation, and biological activity characterization of the novel azaphilones bulbillosins A to E, which are responsible for the ethyl acetate (EtOAc) extract’s toxicity against HeLa cells and *Caenorhabditiselegans*. Furthermore, we studied the localization of the bulbillosins in different plant tissues by MALDI mass spectrometry imaging (MSI).

## Material and methods

### Fungal material

*Astertataricus* plants were initially obtained from SARASTRO-STAUDEN, Austria, and planted in a greenhouse (24 °C, 16 h light per day, light intensity on plant 105 µmol*s^-1^*m^-2^)). Healthy roots and leaves were collected from the plants in October 2019. Surface disinfection and isolation of fungal endophytes were carried out with slight modifications to procedures described earlier (Zhou et al. 2018). The tissues were washed thoroughly with running tap water and neutral soap. In sterile conditions, the samples were immersed in 70 % ethanol solution (v/v) for 1 min and subsequently transferred to 1.5 % sodium hypochlorite solution (with 2 drops of tween 20 in 500 mL sodium hypochlorite solution) for 10 min. The surface-disinfected tissues were rinsed three times with sterile distilled water and dried with sterile filter paper. The surface-disinfected samples were aseptically cut into small fragments (5 mm^2^), evenly placed on petri dishes containing malt extract agar (MEA, malt extract 13 g/L, dextrin 3 g/L, glycerol 2.4 g/L, gelatin peptone 1 g/L, agar 15 g/L, before autoclaving) medium supplemented with chloramphenicol 250 mg/L to suppress bacterial growth according to established protocols (Manganyi et al. 2018). The last washing was used as negative control. Colonies grown on each plate were distinguished based on their surface appearance such as texture and color. The distinguishable colonies were sub-cultured several times on MEA plates, and incubated at 25 °C for 7–10 days until obtaining pure strains.

### Morphological characterization

Reproductive structures were described from the fungus growing on oatmeal agar (Sigma–Aldrich, St. Louis, MO, USA). Measurements were made for 30 replicates of each structure. Photomicrographs were taken with a Keyence VHX-970F microscope (Neu-Isenburg, Germany) and a Nikon eclipse Ni compound microscope, using a DS-Fi3 (Nikon, Tokyo, Japan) and NIS-Elements imaging software v. 5.20. Culture characteristics were described for colonies growing on malt extract agar (MEA, HiMedia, Mumbai, India), OA, potato carrot agar (PCA, HiMedia), and potato dextrose agar (PDA, HiMedia) at 25 °C. Colony colors were annotated following The Royal Horticultural Society London (1996) (O’Donnell and Cigelnik 1997).

### DNA isolation, amplification and phylogenetic study

DNA of the fungus was extracted and purified directly from a colony growing on yeast-malt extract agar (YM agar, malt extract 10 g/L, yeast extract 4 g/L, d-glucose 4 g/L, agar 20 g/L, pH 6.3 before autoclaving), following the Fungal gDNA Miniprep Kit EZ-10 Spin Column protocol (NBS Biologicals, Cambridgeshire, UK). The amplification of the internal transcribed spacer (ITS) regions and the large subunit (LSU) of the nuclear ribosomal RNA (rRNA) gene complex and partial fragments of the second largest subunit of DNA directed RNA polymerase II (*rpb2*) and beta-tubulin (*tub2*) genes was performed according to (White et al. 1990) (ITS), (Vilgalys and Hester 1990) (LSU), (Miller and Huhndorf 2005) (*rpb2*), and (O’Donnell and Cigelnik 1997) and (Groenewald et al. 2003) (*tub2*). The PCR reactions were carried out using the JumpStart™ Taq ReadyMix™ (Sigma–Aldrich, St. Louis, MO, USA), and the products were sequenced using the Sanger Cycle Sequencing method at Microsynth Seqlab GmbH (Göttingen, Germany). Consensus sequences were obtained using Geneious 7.1.9 (Kearse et al. 2012).

The phylogenetic analysis was carried out based on the combination of the four loci of our isolate and type material of selected members of the *Chaetomiaceae*, including the genera more related to *Tengochaeta* according to the phylogenetic analysis done by Wang et al. (2022), and *Jugulosporavestita* and *Pseudorhypophilamangenotii* as outgroup (Suppl. material 1: table S1). Each locus was aligned separately using MAFFT v. 7 (Katoh and Standley 2013), and manually corrected in MEGA v. 10.2.4 (Kumar et al. 2018). Loci were concatenated after visually checking for no conflicts. The maximum-likelihood (ML) and Bayesian inference (BI) methods were performed for the phylogenetic analysis as described earlier (Harms et al. 2021). Bootstrap support (bs) ≥ 70% and posterior probability values (pp) ≥ 0.95 were considered significant (Alfaro et al. 2003). The sequences generated in this study were deposited in GenBank (Suppl. material 1: table S1), and the alignments of each locus are available in the Suppl. material 1.

### Cultivation and extraction of *T.bulbillosa*

Pieces of MEA agar plates well-colonized with *T.bulbillosa* were placed on MEA agar in petri dishes (total 5 L of medium). The comparison between MEA and MEB showed that in MEB medium, the compounds of interest were not produced. The petri dishes were incubated at 25 °C and 16 h light per day (light intensity on fungi 105 µmol*s^-1^*m^-2^, determined using a light meter, Li-Cor LI-250A) for 21 days (Barolo et al. 2023). The fungus and the agar were homogenized with an Ultra-Turrax and subsequently extracted four times with 7 L of ethyl acetate (EtOAc). Each solvent extraction took place overnight while stirring at 175 rpm. The solvent was removed under reduced pressure using a rotary evaporator to yield 1.3 g of extract.

### Data processing with MZmine

The raw HPLC-MS data of *T.bulbillosa*EtOAc extract generated after cultivation on MEA or in MEB were converted to the .mzXML format with using MS Convert (v. 3.0.24002-c5ebe15-proteowizard) (Chambers et al. 2012). The converted positive ionization mode files were processed with MZmine 4.1.0 (Schmid et al. 2023). The parameters for processing were as follows: HPLC: RT wavelet range, 0.3–12 min; maximum peaks in chromatogram, 15; minimum consecutive number of scans, 4; approximate feature FWHM, 0.08 min; RT tolerance intra-sample, 0.04 min; RT tolerance sample-to-sample, 0.20 min). Orbitrap: ion mode, positive; noise threshold by factor of lowest signal, MS^1^ 3.0 and MS^2^ 2; minimum feature height, 1.0E^5^; *m/z* tolerance scan-to-scan, 0.0020 or 10 ppm; *m/z* tolerance intra-sample, 0.0015 or 3 ppm; *m/z* tolerance sample-to-sample, 0.0015 or 5 ppm. Filters: original feature list, Keep original feature list; minimum samples per aligned feature max of 1 sample or 0.0 %). MEAEtOAc extract was used as blank. The resulting aligned peak lists were exported using the GNPS-Feature Based Molecular Networking (v.28.2) and Sirius/CSI FIngerID modules.

### GNPS feature-based molecular networking

The Feature-Based Molecular Networking analyses (GNPS, v 28.2) (Wang et al. 2016; Nothias et al. 2020), positive ionization mode, was created with the GNPS default parameters. The precursor ion mass tolerance was 0.02 Da, the MS/MS fragment ion mass tolerance 0.02 Da. The edges were filtered for a cosine score above 0.7 and at least 6 matched peaks. For the GNPS automatic library search, all matches were required to have a score above 0.6 and at least 6 matched peaks. Cytoscape 3.10.2 was used for molecular network visualization.

### Metabolite annotation using SIRIUS

The sirius_specs.*mgf* file from MZmine was processed with Sirius (v 6.0.1) (Dührkop et al. 2019). The parameters used were as follows: Possible ionizations: [M + H]^+^, [M + Na]^+^, [M + K]^+^, [M-H_2_O + H]^+^, [M+H_2_O + H]^+^; Allowed elements in molecular formula: H,C,N,O,P,S,Cl; Instrument profile: Orbitrap; mass accuracy: 5 ppm for MS^2^, DB for molecular formulas and structures: BIO, maximum *m/z* to compute: 850 with H,C,N,O,S,Cl as allowed elements. The prediction of the chemical class was made with CANOPUS (Djoumbou Feunang et al. 2016; Dührkop et al. 2021) using the NPClassifier taxonomy (Kim et al. 2021).

### Isolation of compounds

The EtOAc extract was redissolved in MeOH and fractionated using flash chromatography with a RP-18 cartridge (CHROMABOND® Flash RS 40 C_18_ec, 176 × 26.7 mm, 43 g sorbent, Macherey-Nagel GmbH & Co.KG, Düren, Germany), eluting with H_2_O (A) and CH_3_CN (B) using a gradient from 5 to 100% B (0–25 min) followed by 100% B (25–30 min), flow rate 20 mL/min. Time-based fractionation resulted in 15 fractions. The solvent was removed from the fractions using a vacuum centrifuge. Fractions 11 to 13 contained the compounds of interest.

Compound isolation was performed by HPLC (Dionex UltiMate 3000, Thermo Fisher Scientific) equipped with an F5 column (Kinetex F5, 5 µm, 100 Å, 250×10 mm, Phenomenex) using H_2_O (A) and CH_3_CN (B) (0.1% trifluoroacetic acid each) and the following gradients: Fraction 11 (40 to 50% B (0–3 min), 50 % B (3–25 min), 50 to 100% B (25–26 min), 100 % B (26–31 min) flow rate 4.7 mL/min); Fraction 12 (40 to 50% B (0–3 min), 50 % B (3–22 min), flow rate 4.7 mL/min); Fraction 13 (50 to 60% B (0–3 min), 60 % B (3–22 min), flow rate 4.7 mL/min). The purity of the compounds was assessed by HPLC-MS as described above.

### Spectroscopic and spectrometric analysis of the isolated compounds

CD spectra were recorded on an Olis CD spectrophotometer (Athens, Georgia, USA), model DSM 20. NMR spectra were recorded in CD_3_CN on a Bruker Avance NEO (5mm QCI-P cryo-probe, sample temperature 300K) or a JEOL ECZ600R spectrometer, both operating at 600.13 MHz (^1^H) and 150.1 MHz (^13^C) using standard parameters. Chemical shifts were referenced to the residual solvent signals (*δ*_H_ 1.94, *δ*_C_ 1.33/118.26). NMR data were analyzed with MestReNova v. 12.0.0-20080. High-resolution electrospray ionization mass spectrometry (HRESIMS^2^) data were acquired on a Q Exactive Plus mass spectrometer (Thermo Fisher Scientiﬁc, Waltham, Massachusetts, USA) equipped with a heated ESI interface coupled to an UltiMate3000 HPLC system (Thermo Fisher Scientiﬁc). The following parameters were used for the data acquisition: pos. and neg. ion mode, ESI spray voltage 3.5 kV, scan range *m/z* 133–2000. Chromatography was performed on a Kinetex C18 column (50 × 2.1 mm, 2.6 μm, 100 Å; Phenomenex, California, USA) with H_2_O (A) and CH_3_CN (B) (0.1 % formic acid each), using a gradient from 5 % to 100 % B (0–16 min) followed by 100 % B (16–20 min), flow rate 0.4 mL/min. Data were evaluated with FreeStyle 1.6 (Thermo Fisher Scientific).

### Mosher’s derivatization

The derivatization procedure was performed directly in the NMR tube (Orlov and Ananikov 2011). For (*R*)-MPTA ester preparation, 0.3 mg of **1** were dissolved in 500 µL of pyridine-d_5_ and 2 mg (*S*)-MPTA chloride was added. The mixture was kept at 25 °C overnight (Surup et al. 2013); ^1^H NMR (pyridine- d5, 600 MHz, only signals assigned for derivatized 4-hydroxy-2-methyl-6-oxocyclohex-1-en-1-yl moiety): d 2.933 (12-H_b_), d 2.907 (12-H_a_), 2.771 (14-H_b_), d 2.740 (14-H_a_), d 1.750 (16-H_3_). The (*S*)-MPTA ester was prepared in the same manner by the addition of (*R*)-MPTA chloride; ^1^H NMR (d_5_-pyridine, 600 MHz, only signals assigned for derivatized 4-hydroxy-2-methyl-6-oxocyclohex-1-en-1-yl moiety): d 2.932 (12-H_b_), d 2.905 (12-H_a_), 2.777 (14-H_b_), d 2.746 (14-H_a_), d 1.747 (16-H_3_).

### MALDI-mass spectrometry imaging

#### MSI sample preparation

Flower, leaf, stem, rhizome, and root from *A.tataricus* were harvested, embedded in a gelatin solution (10 %, w/v), and immediately frozen in liquid nitrogen to form a solid block. Embedded samples were stored at -70 °C until sectioning. The tissues were sectioned with a thickness of 14 μm at -21 °C using a cryotome (MICROM HM 500 M, MICROM International GmbH, Walldorf, Germany) and thaw-mounted on VWR Superfrost Plus slides. The samples were dried in a desiccator for 15 min, and stored at -70 °C. The samples were first observed using an inverse microscope (Axio Observer, ZEISS, Jena, Germany), images were taken with an Axiocam 712 color digital camera (ZEISS) for later comparison with the MSI results. The tissue sections were coated with 25 mg/mL super-DHB in CH_3_CN/H_2_O (1:1 v/v - 0.1 % trifluoroacetic acid), using a pneumatic sprayer (SunChrome, Friedrichsdorf, Germany). Nitrogen was used as spraying gas, with a total amount of 18.96 μg/mm^2^ super-DHB. For all the slides, the first three layers were sprayed with a reduced flowrate.

#### Image acquisition

Atmospheric pressure MALDI-MSI measurements were performed on a Fourier transform orbital trapping mass spectrometer (Q Exactive Plus, Thermo Fisher Scientific) equipped with an AP-MALDI (ng) UHR source (MassTech Inc, Columbia, Maryland USA) with a laser spot size <10 μm. Imaging experiments were conducted in positive ion mode for 100–1100 *m/z* with 140,000 resolution at *m/z* 200, one microscan, 5×10^6^ AGC target, 500 ms maximum injection time, 4.5 kV spray voltage, 450 °C capillary temperature and 60 % for the S-lens RF value. The MALDI source parameters were adjusted as follows: CSR mode (Constant Speed Rastering), scanning velocity 2.3 mm/min for 20 µm and 3.45 mm/min for 30 µm pixel size, pulse rate 6 kHz, laser energy 31 %. The centroid raw data were converted from the Thermo raw files to imzML using the MassTech imzML Converter (ng) 1.0.1 (merge strategy “Average”) and normalized by TIC. The converted files were analyzed with MSi Reader (v 1.01). All images were linear interpolated in order 3, with *m/z* ± 5 ppm tolerance. SMART parameters (Xi et al. 2023) see Suppl. material 1: table S2.

### Bioactivity assays

#### Assay with *Caenorhabditiselegans*

To assess the toxicity of *T.bulbillosa*EtOAc extract for *C.elegans*, a plate-based toxicity assay was performed as reported elsewhere (Hunt 2017; Xiong et al. 2017) with some minor modifications in the protocol. Briefly, age synchronized populations were obtained by washing worms from maintenance plates with M9 buffer prior to bleaching gravid adults using 1 mL 5 N NaOH solution and 2 mL 5 % sodium hypochlorite solution. Worms dissolved after periodically shaking for a total of 8 min. The lysate was washed three times with M9 buffer, and eggs were separated from debris by density gradient centrifugation using a 60 % sucrose (w/v) solution. The pellet was washed 3 times with 10 mL M9-buffer and centrifuged after each wash. (Peixoto et al. 2016). The eggs were left in M9-buffer to hatch overnight. Before performing the worm viability rate assays, the freshly hatched L1 larvae were transferred to NGM agar plates seeded with *E.coli* OP50 for 48 h until they reached the L4-adult stadium. Each well of a 24-well plate containing 500 µl NGM agar was preseeded with 20 µL of an *E.coli* OP50 suspension (OD600 0.7). After incubating the plates for 24 h, the bacterial lawn had developed evenly. The extracts were freshly dissolved in 10 % DMSO (v/v). 30 µL of the solution were applied to the wells. To calculate the final exposure concentration of carrier solvent and extracts, the volume of agar was taken into account. Each well had a final DMSO concentration of 0.5 %, which also served as (solvent) control. A minimum of 15 adult worms were transferred to each well to start the assay. Viability rate was assessed in triplicates after 24 h.

#### Cell culture and cell survival rate

HeLa cells were maintained in Dulbecco’s modified Essential Medium (DMEM) supplemented with 10 % fetal bovine serum, which was heat-inactivated at 60 °C for 30 min, and glutamine (2 mM). Cells were cultured at 37 °C in a humidified atmosphere containing 5 % CO_2_. For estimation of the cell viability against the EtOAc extract dissolved in 10 % DMSO, a serial dilution of the test extracts from 1 to 0.125 mg/mL DMEM supplemented with 10 % fetal bovine serum was prepared in 96-well flat-bottom polystyrene microplates. Doxorubicin solution (100 µM) was used as control. Cells with concentration of 5×10^4^ cells/per well were used. 100 μL of cell suspension were added to a 96-well plate containing the serial dilution of the test compounds. A growth control (cells in medium without extract) and a sterility control (medium only) were added. After 48 h of incubation at 37 °C, the cells were fixed with 100 μl cold 10 % trichloroacetic acid (incubation 1 h, 4 °C), which was subsequently washed out. 100 μL of 0.057 % SRB solution (in 1 % acetic acid) was added to each well, followed by 30 minutes of further incubation at 4 °C. The dye was removed from the wells, each well was washed three times with 200 µl acetic acid (1 %) and dried. Before analysis, 200 μl Tris buffer (10 mM; pH 10.5) were added to each well. Absorbance was determined using a TECAN infinite M200 Pro plate reader at 510 nm. Cell viability was calculated in relation to the growth control. All experiments were performed in duplicate.

#### NCI-60 human tumor cell lines screen

Compound **1** was submitted to the NCI-60 panel. Initially, **1** was tested at a single high dose of 10 µM. Subsequently, the compound was tested in the five-dose screen. The standard operating protocol for the NCI-60 cell line screen has been well-documented (Shoemaker 2006; Cawrse et al. 2019; National Cancer Institute 2021; Irungu et al. 2024), as well as the cells lines included in the NCI-60 screen (National Cancer Institute 2015).

### List of abbreviations

**CD** Circular dichroism

**CD_3_CN** Acetonitrile-d_3_

**COSY** Correlation spectroscopy

**DHB** 2,5-dihydroxybenzoic acid

**DMEM** Dulbecco’s modified Essential Medium

**DMSO** Dimethyl sulfoxide

**DNA** Deoxyribonucleic acid

**ESI** Electrospray ionization

**EtOAc** Ethyl acetate

**GNPS** Global natural product social molecular networking

**GP** Growth percent

**HMBC** Heteronuclear multiple bond correlation

**HPLC** High-performance liquid chromatography

**HPLC-DAD** High-performance liquid chromatography coupled with diode-array detection

**HPLC-MS** High-performance liquid chromatography coupled with electrospray ionization tandem mass spectrometry

**HRESIMS** High-resolution electrospray ionization mass spectrometry

**HRMS** High resolution mass spectrometry

**HSQC** Heteronuclear single quantum coherence

**ITS** Internal transcribed spacer

**LSU** The large subunit

**M9** Minimal medium

**MALDI** Matrix-assisted laser desorption ionization

**MEA** Malt extract agar

**MEB** Malt extract broth

**MeOH** Methanol

**MS/MS** Tandem mass spectrometry

**MSI** Mass spectrometry imaging

**MTPA** Mosher’s acid

**NCI** National cancer institute

**NGM** Nematode growth medium

**NMR** Nuclear magnetic resonance

**NOESY** Nuclear overhauser effect spectroscopy

OA Oatmeal agar

**PCA** Potato carrot agar

**PCR** Polymerase chain reaction

**PDA** Potato dextrose agar

***rpb2*** RNA polymerase II second-largest subunit

**rRNA** Ribosomal RNA

**RT** Retention time

**SRB** Sulforhodamine B

***tub2*** Beta-tubulin

**YM** Universal medium for yeasts

## Results and discussion

The discovery of the astin-producing endophyte *C.asteris* motivated us to take a closer look at other potentially secondary metabolite-producing endophytes that can be isolated from the plant *A.tataricus*. Thus, we isolated additional fungal endophytes from different tissues of the plant.

To prioritize the isolated strains, the fungi were cultivated on malt extract agar (MEA) in a small scale, and their extracts were analyzed by HPLC-MS, followed by data evaluation using GNPS (Aron et al. 2020) and Cytoscape (Shannon et al. 2003). Furthermore, the extracts were assessed for toxicity using the nematode *C.elegans* as well as HeLa cells. One of the isolated strains showed pronounced toxicity against both *C.elegans* and HeLa cells (Suppl. material 1: table S3). In addition, GNPS analysis of the extract showed an interesting cluster with thirteen nodes that could not be dereplicated (Suppl. material 1: fig. S1), and which were found to contain the major compounds in the chromatogram of the extract when the strain was cultivated on MEA but not malt extract broth (MEB, Suppl. material 1: figs S2–S4). Finally, a superficial taxonomical examination of this strain suggested it to be a novel species. Thus, we continued working with this strain, as described in the following.

### Phylogenetic analysis of the fungal endophyte

**Figure 1.**
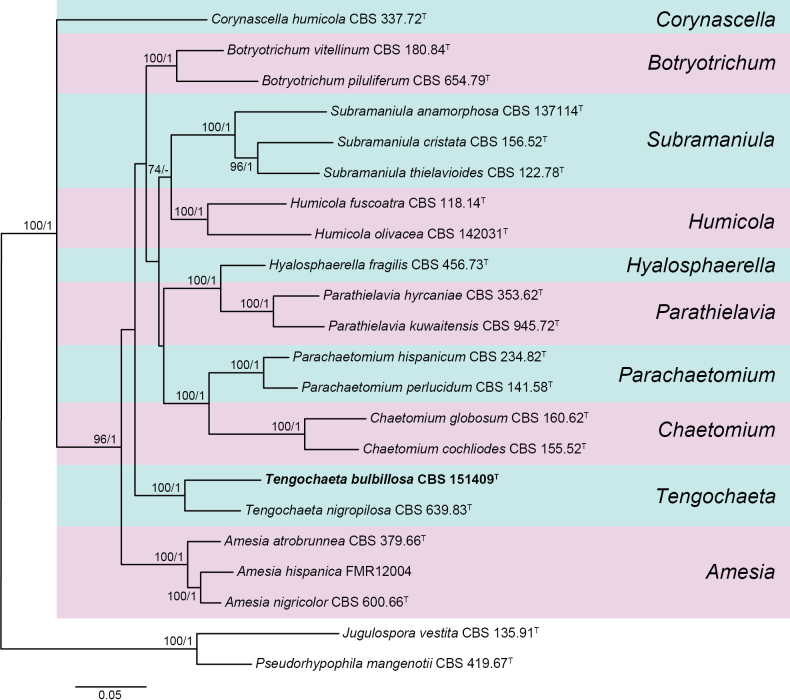
RAxML phylogram obtained from the combined ITS, LSU, *rpb2*, and *tub2* sequences of the isolate, strains belonging to the *Chaetomiaceae*, and *Jugulosporavestita* and *Pseudorhypophilamangenotii* as outgroups. Bootstrap support values ≥ 70/Bayesian posterior probability scores ≥ 0.95 are indicated along branches. Branch lengths are proportional to distance. The novel species is indicated in **bold**. Type material of the different species is indicated by ^T^.

To establish the identity of the selected isolate at species level, a phylogenetic analysis based on multi-gene datasets was conducted in conjunction with a detailed morphological characterization. The combined dataset consisted of 2713 bp, of which 611 bp corresponded to ITS, 832 bp to LSU, 524 bp to *rpb2*, and 746 bp to *tub2*. In the phylogenetic tree (Fig. 1), our isolate was located in a well-supported clade (100 bs/1 pp) representing the genus *Tengochaeta*. However, it was phylogenetically distant from *T.nigropilosa*, which is the only species in this genus to date, suggesting that it represented a new species. Both species also showed morphological differences, so a new species is hereby introduced.

### Taxonomy

#### Tengochaeta bulbillosa

Taxon classificationAnimaliaSordarialesChaetomiaceae

Y. Marin & D. Barrera-Adame sp. nov.

532E9550-2C0B-5285-8AE8-D5A65F688B82

856463

Fig. 2

##### Etymology.

Named after the formation of bulbils.

**Figure 2.**
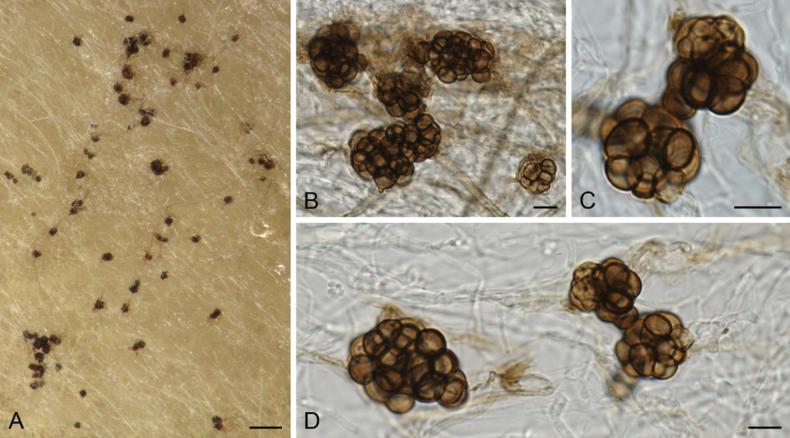
*Tengochaetabulbillosa* sp. nov. (CBS 151409^T^). **A–D** Bulbils. Scale bars: 100 µm (**A**); 10 µm (**B–D**).

##### Type material.

AUSTRIA: Kammer, Sarastro Stauden, roots of *Astertataricus*, Oct. 2019, isol. D. A. Barrera-Adame, ident. Y. Marin-Felix (holotype CBS H-25355; ex-type cultures CBS 151409).

##### Description.

Mycelium composed of hyaline to subhyaline or pale brown, septate, smooth-walled to verrucose, branched hyphae, 1–5 µm diam. Bulbils spherical to irregular, pale brown to dark brown, up to 9.5–70 µm diam, composed of globose to ellipsoidal or irregularly globose, pale brown to brown, smooth and thick-walled cells, 4.5–11 µm diam.

##### Culture characters.

Colonies on MEA attaining a diam. of 35–45 mm in 7 d at 25 °C, cottony, umbonate, circular to slightly lobate, margins fringed, grayed yellow (162A–D); reverse yellow orange (17A–D) and center grayed orange (166B). Colonies on OA attaining a diam. of 52–55 mm in 7 d at 25 °C, velvety to cottony, umbonate, circular, margins regular, grayed orange (167A–B), margins transparent to white, mycelia and center orange white (159B–C); reverse grayed orange (177B–C). Colonies on PCA attaining a diam. of 48–52 mm in 7 d at 25 °C, cottony, hemispherical, circular to slightly lobate, margins fringed, yellow orange (16B–D) and white mycelia; reverse orange (26A–B), margins yellow (13B–C), and center grayed orange (177A). Colonies on PDA attaining a diam. of 30–32 mm in 7 d at 25 °C, velvety to cottony, center folded, circular to slightly lobate, margins fringed, yellow (12C–D), ring yellow orange (20C), margins white; reverse yellow orange (20A–C) with center grayed orange (174B).

##### Notes.

*Tengochaetabulbillosa*, which produces bulbils, is only the second species reported in the genus. *Tengochaetanigropilosa*, which was isolated from soil in a *Pinus* forest in Spain, produces sexual morph characterized by ascomata with flexuous to undulate hairs, pyriform or broadly clavate asci and ellipsoidal to fusiform ascospores. No asexual morph has been observed in the latter species.

Bulbils have only been reported in two other genera of the family *Chaetomiaceae*, i.e. *Subramaniula* and *Trichocladium* (Wang et al. 2022). However, in the latter genus, the propagules are hyaline, while in our new species of *Tengochaeta*, they are pale brown to dark brown. The bulbils reported in different species of *Subramaniula*, i.e. *S.anamorphosa*, *S.asteroides* and *S.obscura*, are also pigmented (Ahmed et al. 2016). Bulbils in *T.bulbillosa* are smaller (9.5–70 µm diam) than in *S.asteroides* (58–100 × 44–71 µm) and *S.obscura* (27–73 × 20–36 µm). Comparison of the size of bulbils with *S.anamorphosa* is not possible due to the lack of this information in the original description. Moreover, cells composing the bulbils are larger in *S.asteroides* (7–12 × 7–9 µm) than in *T.bulbillosa* (4.5–11 µm diam) (Ahmed et al. 2016).

### Isolation and structure elucidation of bulbillosin A – E

Bulbillosins A to E (**1**–**5**, Fig. 3) were isolated from the EtOAc extract of *T.bulbillosa* using Flash Chromatography and semi-preparative HPLC. Their planar structures were elucidated using mass spectrometry and NMR spectroscopy (Suppl. material 1: figs S5–S33), stereochemistry was assigned using CD spectroscopy and Mosher’s method.

**Figure 3.**
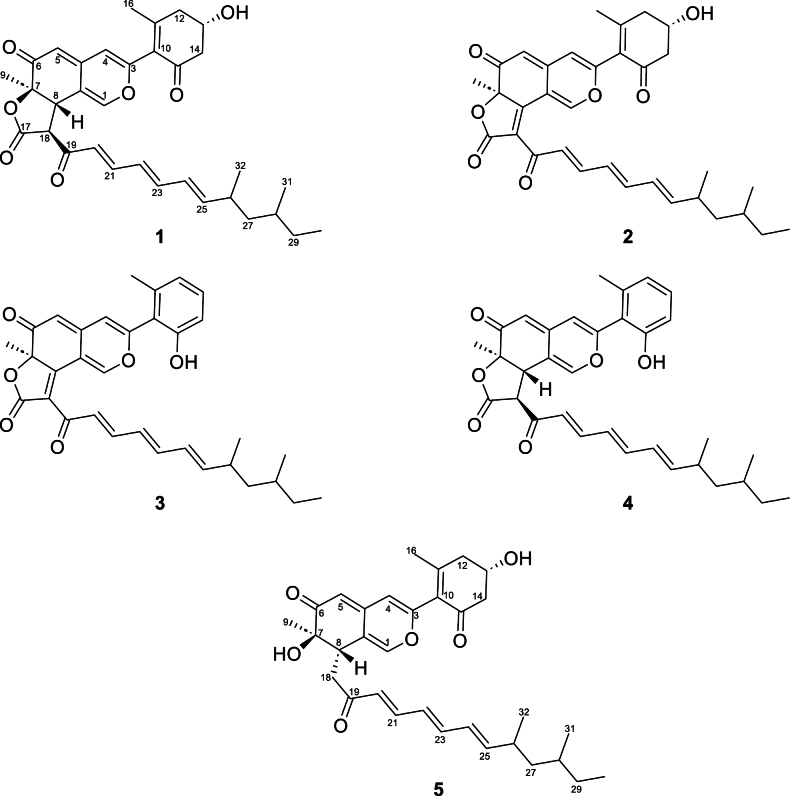
Compounds isolated from *Tengochaetabulbillosa*: bulbillosin A–E (**1**–**5**).

**Figure 4.**
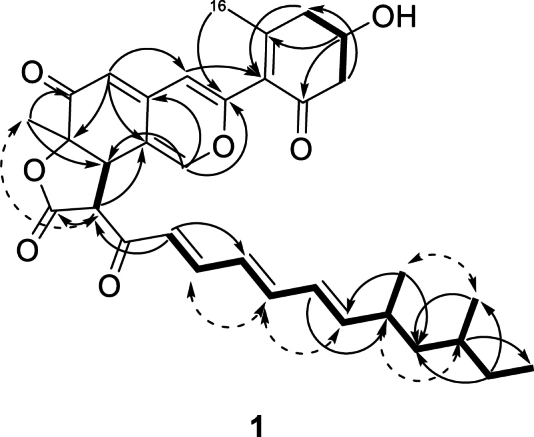
Selected ^1^H-^1^H COSY (bold), HMBC (arrows), and NOESY (dashed arrows) correlations in the planar structure of **1**.

Compound **1** was isolated as yellow amorphous solid. The molecular formula C_33_H_39_O_7_ was calculated for the [M+H]^+^ ion at *m/z* 547.2687 (Δ 0.5 ppm). The ^1^H NMR spectrum of **1** in CD_3_CN (Table 1) displayed the typical pattern of an azaphilone scaffold with three olefinic protons at 7.12, 6.19, and 5.33 ppm (H-1, H-4, and H-5). A methyl group was found to be attached at C-7. The substituents in positions C-8 and C-18 of the lactone were determined to be in *trans* configuration (large coupling constant *J*_8,18_ = 12.8 Hz, absence of a NOESY correlation between H-8 and H_3_-9). Additionally, the NOESY spectra showed correlations between H_3_-9 and H-18 (Fig. 4). Consequently, 6a*R*,9*S*,9a*S* configuration was proposed for **1**. HSQC and HMBC spectra indicated the presence of two fragments connected to the main azaphilone skeleton at C-3 and C-18. Comparison of the ^1^H NMR spectrum with literature data suggested that the first fragment is 4-hydroxy-2-methyl-6-oxocyclohex-1-enyl, also found in cohaerins C, D, and F (Quang et al. 2005b; Quang et al. 2006), and minutellins A and B (Kuhnert et al. 2017). Indeed, correlations between H-4 and C-10 as well as H-16 and C-3 in the HMBC spectrum confirmed this cyclohexanone is attached to C-3. The absolute confi­guration of C-13 was determined to be *S* after derivatization with Mosher’s acid (MTPA, α-methoxy-α-(trifluoromethyl-)phenylacetic acid) (Orlov and Ananikov 2011). Negative Δδ^*SR*^ values for H_a_-12, H_b_-12 and positive Δδ^*SR*^ values for H_a_-14 and H_b_-14 (Suppl. material 1: fig. S11) agree with the findings for the structurally related cohaerins C, D, F, G and I (Surup et al. 2013). The absolute configuration of C-7 was determined to be *R* by CD spectroscopy. The spectrum showed a positive Cotton effect at 377 nm, like the cohaerins C to I and K (Quang et al. 2006; Surup et al. 2013), sassafrins A and B (Quang et al. 2005a) but opposed to chaetoviridin A (Takahashi et al. 1990). The second fragment was identified as 8,10-dimethyldodecyl-2,4,6-trienone from 1D and 2D NMR spectra, connected to C-18 (HMBC correlation between H-20 and C-18). The COSY spectrum showed all required correlations between the aliphatic and olefinic protons of this acyl side chain. The *E*-geometry of the conjugated double bonds in the side chain of **1** was assigned on the basis of the respective coupling constants (*J*_20,21_ = 15.3 Hz, *J*_21,22_ = 11.3 Hz, *J*_22,23_ = 14.7 Hz, *J*_23,24_ = 10.7 Hz, *J*_24,25_ = 15.2 Hz) (Schmidt et al. 2002) and by NOESY correlations between H-21/H-23 and H-23/H-25. The side chain features two methyl groups. HMBC correlations linked H-24 to C-26, H-31 (-CH_3_) and H-32 (-CH_3_) to C-27, and H-31 (-CH_3_) to C-29. The relative configurations of C-26 and C-28 were deduced based on the comparison of experimental and calculated ^13^C NMR shifts for *anti*- and *syn*-isomers of comparable structures in the literature (Suppl. material 1: table S4) (Stahl et al. 1996; Cheng et al. 2006). The chemical shift difference between C-31 and C-32 in **1** is 2.1 ppm, suggesting *syn*-configuration. Additionally, this configuration is supported by NOESY correlations between H-31/32 and H-26/28. Compound **1** was thus deduced to be (6a*R*,9*S*,9a*S*)-9-((2*E*,4*E*,6*E*)-8,10-dimethyldodeca-2,4,6-trienoyl)-3-((*S*)-4-hydroxy-2-methyl-6-oxocyclohex-1-en-1-yl)-6a-methyl-9,9a-dihydro-6*H*-furo[2,3-*h*]isochromene-6,8(6a*H*)-dione which we named bulbillosin A.

**Table 1.** ^1^H NMR data (CD_3_CN, 600 MHz) for bulbillosins A-E (1–5).

Position	1	2	3	4	5
1	7.12 (m)	8.65 (m)	8.75 (m)	7.19 (overlapped)	6.99 (m)
4	6.19 (s)	6.27 (s)	6.48 (d, 2.8)	6.40 (s)	6.11 (s)
5	5.33 (d, 1.2)	5.31 (d, 1.0)	5.33 (d, 2.8)	5.35 (s)	5.38 (s)
8	3.85 (dd, 12.8, 1.9)	-	-	3.89 (dd, 12.8, 1.7)	3.29 (m)
9	1.37 (s)	1.66 (s)	1.69 (d, 2.9)	1.40 (s)	1.08 (s)
12	2.77 (dd, 18.5, 4.2)	2.79 (dd, 18.6, 4.2)	6.83 (overlapped)	6.80 (overlapped)	2.75 (dd, 15.9, 3.8), 2.48 (m)
13	4.22 (ddt, 7.9, 6.3, 4.0)	4.24 (ddt, 7.9, 6.3, 4.0)	7.23 (t, 7.4)	7.19 (overlapped)	4.21 (ddd, 11.3, 7.7, 4.0)
14	2.66 (dd, 16.0, 3.7)	2.67 (dd, 16.0, 3.8)	6.81 (overlapped)	6.80 (overlapped)	2.65 (dd, 15.9, 3.8), 2.45 (m)
16	1.99 (s)	2.04 (s)	2.27 (s)	2.21 (overlapped)	1.96 (overlapped)
17	-	-	-	-	3.18 (dd, 17.4, 2.2)
18	4.58 (d, 12.8)	-	-	4.61 (d, 12.8)	3.01 (dd, 17.4, 9.8)
20	6.50 (d, 15.3)	6.91 (d, 15.1)	6.94 (d, 14.8)	6.52 (d, 15.3)	6.23 (m)
21	7.47 (dd, 15.0, 11.3)	7.38 (dd, 15.1, 11.5)	7.40 (dd, 15.0, 11.6)	7.49 (dd, 15.3, 11.3)	6.70 (dd, 15.1, 10.6)
22	6.44 (dd, 14.7, 11.2)	6.41 (dd, 14.9, 11.4)	6.42 (dd, 14.8, 11.6)	6.44 (dd, 14.9, 11.3)	6.34 (dd, 14.9, 11.1)
23	6.80 (dd, 14.7, 10.7)	6.77 (dd, 14.9, 10.4)	6.76 (overlapped)	6.80 (overlapped)	7.36 (dd, 15.4, 11.2)
24	6.26 (dd, 15.2, 10.8)	6.23 (dd, 15.2, 10.8)	6.24 (dd, 14.9, 11.4)	6.26 (dd, 15.2, 10.8)	6.23 (m)
25	5.93 (dd, 15.2, 8.5)	5.90 (dd, 15.2, 8.5)	5.90 (dd, 14.9, 8.3)	5.93 (dd, 15.3, 8.4)	5.86 (dd, 15.2, 8.6)
26	2.38 (m)	2.36 (overlapped)	2.37 (m)	2.38 (m)	2.36 (m)
27	1.35 (m)	1.33 (m)	1.34 (m)	1.35 (overlapped)	1.33 (m)
	1.10 (m)	1.09 (m)	1.09 (m)	1.11 (overlapped)	1.10 (m)
28	1.31 (m)	1.30 (m)	1.29 (m)	1.31 (overlapped)	1.28 (m)
29	1.28 (m)	1.27 (m)	1.28 (m)	1.27 (overlapped)	1.27 (overlapped)
	1.14 (m)	1.13 (m)	1.13 (m)	1.14 (overlapped)	1.13 (m)
30	0.85 (overlapped)	0.84 (overlapped)	0.84 (overlapped)	0.85 (overlapped)	0.84 (overlapped)
31	0.85 (overlapped)	0.83 (overlapped)	0.84 (overlapped)	0.85 (overlapped)	0.84 (overlapped)
32	1.01 (d, 6.7)	0.99 (d, 6.6, 2H)	1.00 (d, 6.6)	1.01 (d, 6.7)	1.00 (d, 6.6)

Compound **2** was isolated as a yellow amorphous solid. HRMS analysis resulted in a [M+H]^+^ ion at *m/z* 545.2529 (C_33_H_37_O_7_, Δ 0.9 ppm), suggesting this compound has two hydrogens less than **1**. Examination of the ^1^H spectrum showed the absence of H-8 and H-18, and the ^13^C spectrum showed the presence of an *α*, *β*-unsaturated lactone (down-field shift of C-8 and C-18, Table 2) (Quang et al. 2005a). The configuration of C-7 was determined to be *R* by comparison of the CD spectrum of **2** with **1**. The absolute stereochemistry of C-13 is proposed to be *S* as in **1**. Therefore, **2** was determined as (*R*)-9-((2*E*,4*E*,6*E*)-8,10-dimethyldodeca-2,4,6-trienoyl)-3-((*S*)-4-hydroxy-2-methyl-6-oxocyclohex-1-en-1-yl)-6a-methyl-6*H*-furo[2,3-*h*]isochromene-6,8(6a*H*)-dione, which we named bulbillosin B.

Compound **3** was isolated as a yellow amorphous solid. The [M+H]^+^ ion at *m/z* 527.2422 (Δ 1.1 ppm) suggested a molecular formula of C_33_H_35_O_6_, indicating loss of water from **2**. The NMR and CD spectra of **3** resembled those of **2**. The only notable difference in NMR was observed in the signals of the fragment at C-3, which was found to be 2-hydroxy-6-methylphenyl as deduced from its 2D NMR spectra and comparison with the data for cohaerin A, E, H, and K, and minutellins C and D (Quang et al. 2005b; Surup et al. 2013; Kuhnert et al. 2017). **3** was thus assigned as (*R*)-9-((2*E*,4*E*,6*E*)-8,10-dimethyldodeca-2,4,6-trienoyl)-3-(2-hydroxy-6-methylphenyl)-6a-methyl-6*H*-furo[2,3-*h*]isochromene-6,8(6a*H*)-dione, which we named bulbillosin C. Analogously, compound **4** was characterized ([M + H]^+^ at *m/z* 529.2575, Δ 1.9 ppm), a yellow amorphous solid. Compound **4** was identified as (6a*R*,9*S*,9a*S*)-9-((2*E*,4*E*,6*E*)-8,10-dimethyldodeca-2,4,6-trienoyl)-3-(2-hydroxy-6-methylphenyl)-6a-methyl-9,9a-dihydro-6*H*-furo[2,3-*h*]isochromene-6,8(6a*H*)-dione and named bulbillosin D.

**Table 2.** ^13^C NMR data (CD_3_CN, 150 MHz) for bulbillosins A–E (1–5).

Position	1	2	3	4	5
1	145.5	154.3	154.5	145.7	145.6
3	154.9	155.1	156.3	155.9	155.0
4	111.9	113.6	112.9	112.1	111.8
4a	146.1	145.0	145.6	147.2	147.7
5	106.8	106.1	105.9	106.7	105.5
6	192.7	191.3	191.7	192.8	199.4
7	83.6	88.9	88.9	84.1	74.5
8	45.2	166.0	166.3	45.4	41.7
8a	116.7	112.8	112.3	116.9	121.3
9	19.1	26.1	26.2	19.2	21.3
10	131.0	130.2	120.3	120.9	131.5
11	162.4	164.0	139.9	139.9	162.4
12	41.4	41.5	122.6	122.5	41.4
13	65.3	65.5	132.4	132.1	65.6
14	46.6	46.6	114.3	114.3	46.7
15	195.5	194.8	156.3	155.9	194.7
16	22.8	22.9	19.8	19.7	22.8
17	171.5	168.9	166.3	171.8	-
18	51.6	125.9	129.4	51.6	36.8
19	192.3	185.9	185.7	192.2	203.5
20	128.1	127.0	127.0	128.0	129.2
21	147.2	146.5	146.5	147.2	143.4
22	129.4	129.3	129.4	129.2	129.2
23	145.4	145.5	145.4	145.8	144.4
24	129.5	129.3	129.3	129.2	129.2
25	149.3	149.0	149.0	149.0	147.7
26	35.7	35.7	35.6	35.6	35.6
27	44.6	44.5	44.5	44.6	44.7
28	32.9	32.8	32.7	32.8	32.6
29	30.5	30.5	30.4	30.6	30.5
30	11.4	11.4	11.4	11.5	11.4
31	19.2	19.2	19.2	19.2	19.3
32	21.3	21.2	21.2	21.3	21.3

Compound **5** was isolated as a yellow amorphous solid and found to have the molecular formula C_32_H_41_O_6_ ([M + H]^+^ at *m/z* 521.2887, Δ 2.1 ppm). The ^1^H and ^13^C NMR spectra of **5** were similar to those of **1**, except for the lactone ring missing, the appearance of a methylene group C-18 (*δ*_H,C_ 3.01/3.18, 36.8), and a slight difference in the chemical shift of C-8 (*δ*_H,C_ 3.29, 41.7). These chemical shifts were comparable to those of cohaerin F (C-18 *δ*_H,C_ 2.79/3.22, 40.2, C-8 *δ*_H,C_ 3.32, 40.2) and longirostrerone B (C-18 *δ*_H,C_ 2.83/3.40, 36.6, C-8 *δ*_H,C_ 3.34, 41.0) (Quang et al. 2006; Panthama et al. 2011). Overall, the carbon chemical shifts of **5** were more similar to longirostrerone B than to cohaerin F. This suggests the same configuration at C-8 for **5** as found in longirostrerone B (Panthama et al. 2011). The COSY spectrum exhibited correlations between H-18 and H-8. The absolute stereochemistry at C-7 and C-13 was proposed *S* as in **1**. Thus, **5** was identified as (7*R*,8*S*)-8-((3*E*,5*E*,7*E*)-9,11-dimethyl-2-oxotrideca-3,5,7-trien-1-yl)-7-hydroxy-3-((*S*)-4-hydroxy-2-methyl-6-oxocyclohex-1-en-1-yl)-7-methyl-7,8-dihydro-6*H*-isochromen-6-one, which we named bulbillosin E.

### Mass spectrometry imaging

The isolation of natural products produced by endophytes from plant material has been described only in a few reports, examples are the astins from *A.tataricus* / *C.asteris* (Xu et al. 2013; Jahn et al. 2017; Schafhauser et al. 2019), swainsonine from Locoweeds (*Astragalus* and *Oxytropis* species)/ *Undifilumoxytropis*, *Alternariaoxytropis* (Gardner et al. 2003; Yu et al. 2010; Gao et al. 2012; Liang et al. 2021), or the ergot alkaloids from grasses with agronomical interest such as rye, triticale, wheat, oat, and barley/ *Clavicepspurpurea* (Smakosz et al. 2021; Carbonell-Rozas et al. 2023). Using MSI, we were able to detect the presence of the major azaphilones in plant tissues of *A.tataricus*.

**Figure 5.**
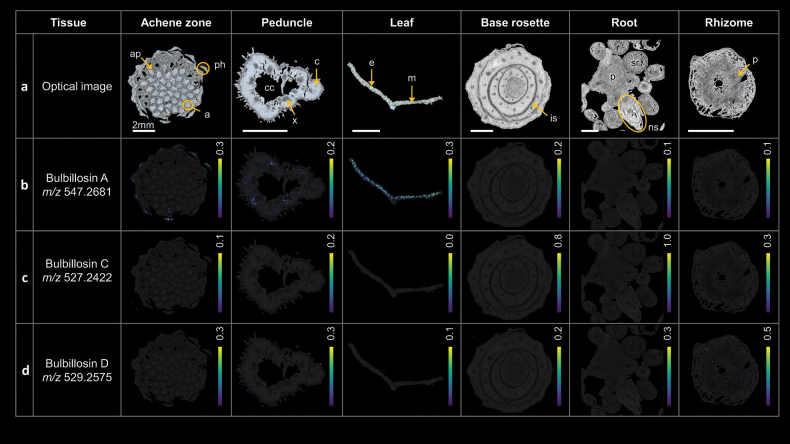
MALDI-MS imaging of different parts of the *A.tataricus* plant. Overviews and descriptions of the tissue sections of the achene zone with achene (a), achene pericarp (ap) and phyllaries (ph), the peduncle with cortex (c), central cavity (cc) and xylem (x), the cross-sectioned leaf with epidermis (e) and mesophyll (m), the base rosette with interleaf space (is), the root with secondary root (sr), new shoots (ns) and pith (p), and the rhizome with pith (p). The relative distributions are displayed as heat maps, with the color code between black (for 0 ions detected) and yellow (for the maximum percentage of ions). The percentages were adjusted by compound normalized ion intensity. The resulting mass spectrometry images were all normalized by TIC. Accurate mass measurements of azaphilones in *A.tataricus* tissues are listed in Suppl. material 1: table S5.

MSI showed that the main compound bulbillosin A (**1**, detected at *m/z* 547.2681, Δ 1.6 ppm) was present in the upper part of the plant (achene zone, peduncle and especially the leaf; Fig. 5). This is interesting because *T.bulbillosa* was isolated from the roots of the plant. This observation suggests that either the fungal endophyte was also present in the aerial part of the plant, or that the azaphilones are transported inside the plant.

Bulbillosin C (*m/z* 527.2422, Δ 1.1 ppm) and bulbillosin D (*m/z* 529.2575, Δ 1.9 ppm) were also detected in the plant. They were found to be much less abundant compared to **1**, similar to cultivation of the fungus on MEA. Bulbillosin C was detected only in the base rosette, where it interestingly could only be detected in the interleaf space (Fig. 5c), where **1** could not be detected. This suggests that the compounds are not randomly distributed in the plant. Bulbillosin D was detected in the lower part of the plant, although present only in very low amounts. Bulbillosins B and E could not be detected in any of the tissues (Suppl. material 1: fig. S34).

### Human tumor cell line screen of bulbillosin A (1)

Bulbillosin A (**1**) was submitted to the NCI-60 human cancer cell line screen (Shoemaker 2006), where it was tested against 60 representative human cancer cell lines including leukemia, lung, colon, central nervous system, melanoma, ovarian, renal, prostate, and breast cancer cell lines. **1** was initially screened at 10 µM concentration (Suppl. material 1: figs S35, S36), and the results are presented in terms of growth percent (GP). GP represents the growth of a treated culture compared to the growth of an untreated culture. GP between 0–99 represents cytostatic properties, GP between -100–0 cytotoxic properties (Cawrse et al. 2019).

Bulbillosin A (**1**) exhibits good cytostatic activity in some of the cell lines like leukemia (CCRF-CEM), colon (HCT-116), renal (UO-31) and prostate (PC-3) cancer, where the growth percentages were less than 3 %, while other cells like CNS (SNB-75) and renal (A498 and TK-10) cancer were less affected, indicating a certain specificity for some cell lines. Additionally, **1** also showed a high lethality for SK-MEL-5 (melanoma) and OVCAR-3 (ovarian cancer) tumor cells, with over 90% cytotoxicity. Due to its interesting profile in the one-dose assay, **1** was selected for further characterization in the NCI-60 five-dose screen (Suppl. material 1: table S6, figs S37–S42). The data has been evaluated for the parameters GI_50_ (concentration causing 50% growth inhibition), TGI (concentration fully inhibiting cell growth), and LC_50_ (concentration killing 50% of the cells). **1** demonstrated GI_50_/TGI/LC_50_ values in the low concentration range against several cell lines (Table 3).

A previous study reported the cytotoxicity of chemically related azaphilones from *S. longirostre*, longirostrerones A–D against MCF7 cells (Panthama et al. 2011). Bulbillosin A (**1**) showed similar activity as longirostrerone B (a C-8/C-18 unsaturated analogue), with LC_50_/IC_50_ values of 32.4 and 38.2 µM, respectively.

**Table 3.** NCI-60 five-dose screen, GI_50_, TGI and LC_50_ values of bulbillosin A (**1**) for the respective three most affected cell lines.

	Cell name	Panel name	Concentration (µM)
GI_50_	OVCAR-3	Ovarian cancer	1.5
	HCT-15	Colon cancer	1.5
	HL-60(TB)	Leukemia	1.6
TGI	OVCAR-3	Ovarian cancer	3.2
	HCT-15	Colon cancer	3.7
	RPMI-8226	Leukemia	6.4
LC_50_	OVCAR-3	Ovarian cancer	6.8
	HCT-15	Colon cancer	11.5
	SK-MEL-28	Melanoma	23.3

## Conclusions

We isolated five new azaphilones, named bulbillosins A to E (**1** to **5**), from the new fungal species *T.bulbillosa*, an endophytic fungus isolated from *A.tataricus*. The bulbillosins feature a new acyl side chain attached to C-18. **1** exhibited moderate but differential anti-cancer activity against several human cell lines. Mass spectrometry imaging analysis showed these compounds to be localized mainly in the aerial tissues of *A.tataricus*, especially **1** in the leaf.

## Supplementary Material

XML Treatment for Tengochaeta bulbillosa

## Additional information

### Adherence to national and international regulations

All the fungal strains used in this study have been legally obtained, respecting the Convention on Biological Diversity (Rio Convention).

### Author ORCIDs

Diana Astrid Barrera-Adame https://orcid.org/0000-0003-2659-8330

Yasmina Marin-Felix https://orcid.org/0000-0001-8045-4798

Ana Kristin Wegener https://orcid.org/0009-0004-0470-9962

Michael Lalk https://orcid.org/0000-0002-9230-0267

Marc Stadler https://orcid.org/0000-0002-7284-8671

Timo H. J. Niedermeyer https://orcid.org/0000-0003-1779-7899

### Data availability

All sequences generated during this study have been submitted to GenBank.

## Supplementary materials

Supplementary material 1 Biological activity results Data type pdf Explanation note Biological activity results, network for sample prioritization, HPLC-MS, UV, and NMR data of isolated compounds, MSI*A.tataricus* tissues, theoretical and observed masses of metabolites in MSI, alignment and additional phylogenetic details of the fungi. https://binary.pensoft.net/file/1253178This dataset is made available under the Open Database License (http://opendatacommons.org/licenses/odbl/1.0/). The Open Database License (ODbL) is a license agreement intended to allow users to freely share, modify, and use this Dataset while maintaining this same freedom for others, provided that the original source and author(s) are credited.Diana Astrid Barrera-Adame, Yasmina Marin-Felix, Ana Kristin Wegener, Michael Lalk, Marc Stadler, Timo H. J. Niedermeyer
