# Supplementary material for: Bulbillosins A - E, azaphilones from Tengochaetabulbillosa sp. nov. (Chaetomiaceae), a root endophyte of the Chinese medicinal plant Astertataricus
Source: IMA Fungus. 2025 Feb 17;16:e141036. doi: 10.3897/imafungus.16.141036 (PMC11882021; doi:10.3897/imafungus.16.141036)
Supplement: Supplementary material 1 — Biological activity results [file imafungus-16-e141036-s001.pdf]

Supporting Information

Bulbillosins A - E, azaphilones from *Tengochaeta bulbillosa* sp. nov. (Chaetomiaceae), a root endophyte of the Chinese medicinal plant *Aster tataricus*

Diana Astrid Barrera-Adame[†], Department of Pharmaceutical Biology, Institute of Pharmacy, Freie Universität Berlin, Königin-Luise-Str. 2+4, 14195 Berlin, Germany, diana.barrera-adame@fu-berlin.de

Yasmina Marin-Felix, Department Microbial Drugs, Helmholtz Centre for Infection Research, Inhoffenstraße 7, 38124 Braunschweig, Germany and Institute of Microbiology, Technische Universität Braunschweig, Spielmannstraße 7, 38106 Braunschweig, Germany, yasmina.marinfelix@helmholtz-hzi.de

Ana Kristin Wegener[‡], Department of Pharmaceutical Biology/Pharmacognosy, Institute of Pharmacy, Martin Luther University Halle-Wittenberg, 06120 Halle (Saale), Germany, anawegener@aol.com

Michael Lalk, Department of Cellular Biochemistry and Metabolomics, Institute of Biochemistry, University of Greifswald, Felix-Hausdorff-Strasse 4, 17487 Greifswald, Germany, lalk@uni-greifswald.de

Marc Stadler, Department Microbial Drugs, Helmholtz Centre for Infection Research, Inhoffenstraße 7, 38124 Braunschweig, Germany and Institute of Microbiology, Technische Universität Braunschweig, Spielmannstraße 7, 38106 Braunschweig, Germany, marc.stadler@helmholtz-hzi.de

Timo H. J. Niedermeyer[†,]*, Department of Pharmaceutical Biology, Institute of Pharmacy, Freie Universität Berlin, Königin-Luise-Str. 2+4, 14195 Berlin, Germany, timo.niedermeyer@fu-berlin.de

[†]part of this work was conducted at the Department of Pharmaceutical Biology/Pharmacognosy, Institute of Pharmacy, Martin Luther University Halle-Wittenberg, Hoher Weg 8, 06120 Halle (Saale), Germany

[‡]current affiliation: Winckelmann Apotheke, 39576 Stendal, Germany

*corresponding author, timo.niedermeyer@fu-berlin.de, +49 30 83875060

Alignment used in the phylogenetic study…………………………………………………...5
Spectroscopic and spectrometric data of the bulbillosins……………………………………...65
References………………………………………………………………………………78

Table S1 Strains of the order Sordariales included in the phylogenetic study. Novel taxon and sequences generated in this study are indicated in **bold**. 4
Table S2 SMART parameters for the MS-Imaging analysis of A. tataricus tissues. 32
Table S3 Initial results of the screening assay for toxicity-/lethality of *T. bulbillosa* EtAcO extract. HeLa cell viability assay n = 2, *C. elegans* viability assay n = 7. 33
Table S4 Comparison of experimental ^{13}C NMR shifts of the side chain of bulbillosins A-E (**1-5**) with calculated ^{13}C NMR shifts of sambutoxin analogues (**6-9**) and experimental ^{13}C NMR shifts (+)-N-deoxymilitarinone A (**10**). 66
Table S5 Accurate mass measurement of azaphilones in A. tataricus tissues using MALDI-MSI. nd: not detected. 68
Table S6 NCI-60 five-dose screen, *in-vitro* testing results for bulbillosin A (**1**). 71

Fig. S1. GNPS network of the T. bulbillosa EtAcO extracts (MEB and MEA), compound classes annotated by GNPS databases and SIRIUS. The azaphilone cluster has shared nodes (m/z 547.2589 and 547.2590), however, the ion intensity of the compounds in the MEB extract is lower than in the MEA extract. 34
Fig. S2. HPLC-MS base peak chromatogram (pos. mode) of *T. bulbillosa* EtAcO extract (MEA medium cultivation). 35
Fig. S3. HPLC-MS base peak chromatogram (positive mode) of *T. bulbillosa* EtAcO extract (MEB medium cultivation). 35
Fig. S4. Venn diagram with detected ions from T. bulbillosa extracts cultivated in MEA and MEB medium. MEA extract used as blank. 35
Fig. S5. HPLC data of bulbillosin A (**1**). a. UV-vis spectrum. b. ESI Full MS in pos. ion mode (BPC). c. ESI Full MS in neg. ion mode (BPC). d. MS/MS in pos. ion mode. 36
Fig. S6. Proton NMR spectrum (600 MHz, CD_3CN) of bulbillosin A (**1**). 37
Fig. S7. COSY NMR spectrum (600 MHz, CD_3CN) of bulbillosin A (**1**). 38
Fig. S8. DEPT-HSQC NMR spectrum (600 MHz, CD_3CN) of bulbillosin A (**1**). 39
Fig. S9. HMBC NMR spectrum (600 MHz, CD_3CN) of bulbillosin A (**1**). 40
Fig. S10. NOESY NMR spectrum (600 MHz, CD_3CN) of bulbillosin A (**1**). 41
Fig. S11. Δd^{SR} values after comparison of the ^{1}H NMR spectroscopic data of the MTPA esters of bulbillosin A (**1**). 42
Fig. S12. HPLC-HRESI data of bulbillosin B (**2**) a. a. UV-vis spectrum. b. ESI Full MS in pos. ion mode (BPC). c. ESI Full MS in neg. ion mode (BPC). d. MS/MS in pos. ion mode. 43
Fig. S13. Proton NMR spectrum (600 MHz, CD_3CN) of bulbillosin B (**2**)............................ 44
Fig. S14. COSY NMR spectrum (600 MHz, CD_3CN) of bulbillosin B (**2**)............................ 45
Fig. S15. HSQC NMR spectrum (600 MHz, CD_3CN) of bulbillosin B (**2**)............................ 46
Fig. S16. HMBC NMR spectrum (600 MHz, CD_3CN) of bulbillosin B (**2**)........................... 47
Fig. S17. HPLC-HRESI data of bulbillosin C (**3**). a. UV-vis spectrum. b. ESI Full MS in pos. ion mode (BPC). c. ESI Full MS in neg. ion mode (BPC). d. MS/MS in pos. ion mode. 48
Fig. S18. Proton NMR spectrum (600 MHz, CD_3CN) of bulbillosin C (**3**)............................ 49
Fig. S19. COSY NMR spectrum (600 MHz, CD_3CN) of bulbillosin C (**3**)............................ 50
Fig. S20. HSQC NMR spectrum (600 MHz, CD_3CN) of bulbillosin C (**3**)............................ 51
Fig. S21. HMBC NMR spectrum (600 MHz, CD_3CN) of bulbillosin C (**3**)........................... 52
Fig. S22. HPLC-HRESI data of bulbillosin D (**4**). a. UV-vis spectrum. b. ESI Full MS in pos. ion mode (BPC). c. ESI Full MS in neg. ion mode (BPC). d. MS/MS in pos. ion mode. 53
Fig. S23. Proton NMR spectrum (600 MHz, CD_3CN) of bulbillosin D (**4**). 54

Fig. S24. COSY NMR spectrum (600 MHz, CD_3CN) of bulbillosin D (**4**). 55
Fig. S25. HSQC NMR spectrum (600 MHz, CD_3CN) of bulbillosin D (**4**). 56
Fig. S26. HMBC NMR spectrum (600 MHz, CD_3CN) of bulbillosin D (**4**). 57
Fig. S27. NOESY NMR spectrum (600 MHz, CD_3CN) of bulbillosin D (**4**).......................... 58
Fig. S28. HPLC-HRESI data of bulbillosin E (**5**). a. UV-vis spectrum. b. ESI Full MS in pos. ion mode (BPC). c. ESI Full MS in neg. ion mode (BPC). d. MS/MS in pos. ion mode. 59
Fig. S29. Proton NMR spectrum (600 MHz, CD_3CN) of bulbillosin E (**5**)............................ 60
Fig. S30. COSY NMR spectrum (600 MHz, CD_3CN) of bulbillosin E (**5**)............................ 61
Fig. S31. HSQC NMR spectrum (600 MHz, CD_3CN) of bulbillosin E (**5**)............................ 62
Fig. S32. HMBC NMR spectrum (600 MHz, CD_3CN) of bulbillosin E (**5**)............................ 63
Fig. S33. NOESY NMR spectrum (600 MHz, CD_3CN) of bulbillosin E (**5**). 64
Fig. S34. Mass spectrometry imaging of A. tataricus tissues without detectable azaphilones. The relative distributions are displayed as heat maps, with the color code between black (for 0 ions detected) and yellow (for the maximum percentage of ions). The percentages were adjusted by compound normalized ion intensity. The resulting mass spectrometry images were all normalized by TIC.. 67
Fig. S35. NCI-60 one-dose screen (10 µM concentration), one dose bar graph for bulbillosin A (**1**). 69
Fig. S36. NCI-60 one-dose screen (10 µM concentration), mean growth chart for bulbillosin A (**1**). 70
Fig. S37. NCI-60 five-dose screen, waterfull grapf GI_{50} for bulbillosin A (**1**)....................... 72
Fig. S38. NCI-60 five-dose screen, waterfull grapf TGI_{50} for bulbillosin A (**1**). 73
Fig. S39. NCI-60 five-dose screen, waterfull grapf LC_{50} for bulbillosin A (**1**)...................... 74
Fig. S40. NCI-60 five-dose screen, mean graphs GI_{50}, TGI_{50} and LC_{50} for bulbillosin A (**1**). 75
Fig. S41. NCI-60 five-dose screen, dose response curves by panel cancer cell lines for bulbillosin A (**1**). 76
Fig. S42. NCI-60 five-dose screen, dose response curves all cancer cell lines for bulbillosin A (**1**). 77

Table S1 Strains of the order Sordariales included in the phylogenetic study. Novel taxon and sequences generated in this study are indicated in **bold**.

**Taxa**	**Strain**	**GenBank Accession Number**				**References**
		**LSU**	**ITS**	***rpb2***	***tub2***	
Amesia atrobrunnea	CBS 379.66[T]	JX280666	JX280771	KX976798	KX976916	(Hoog et al. 2013), (Wang et al. 2016a)
A. nigricolor	CBS 600.66[T]	MH870559	KX976578	KX976802	KX976920	(Wang et al. 2016a), (Vu et al. 2019)
A. hispanica	CBS 149852[T]	OQ100832	OQ100090	OQ108867	OQ108868	(Charria-Girón et al. 2023)
Botryotrichum piluliferum	CBS 654.79[T]	KX976722	KX976597	KX976821	KX976939	(Wang et al. 2016a)
B. vitellinum	CBS 180.84[T]	MZ351421	MZ334725	MZ342979	MZ343018	(Wang et al. 2022)
Chaetomium globosum	CBS 160.62[T]	MH869713	KT214565	KT214666	KT214742	(Wang et al. 2016b), (Vu et al. 2019)
C. cochliodes	CBS 155.52[T]	MH868494	MH856972	KF001811	KC109772	(Wang et al. 2016b), (Vu et al. 2019)
Corynascella humicola	CBS 337.72[T]	MH872209	KX976656	KX976850	KX976998	(Wang et al. 2016a), (Vu et al. 2019)
Humicola fuscoatra	CBS 118.14[T]	MH866152	KX976675	KX976882	KX977017	(Wang et al. 2016a), (Vu et al. 2019)
H. olivacea	CBS 142031[T]	KX976770	KX976676	KX976883	KX977018	(Wang et al. 2016a)
Hyalosphaerella fragilis	CBS 456.73[T]	KX976791	KX976693	KX976907	KX977042	(Wang et al. 2016a)
Jugulospora vestita	CBS 135.91[T]	MT785872	MT784135	MT783824	-	(Marin-Felix et al. 2020)
Parachaetomium	CBS 234.82[T]	MK919304	MK919304	MK919361	MK919418	(Wang et al. 2022)
Pc. perlucidum	CBS:141.58[T]	MH869266	MH857726	MK919365	MK919422	(Vu et al. 2019), (Wang et al. 2022)
Parathielavia hyrcaniae	CBS 353.62[T]	KM655368	KM655329	KX976908	KX977043	(van den Brink et al. 2015), (Wang et al. 2016a)
Pt. kuwaitensis	CBS 945.72[T]	MH872326	KM655332	KX976909	KX977044	(van den Brink et al. 2015), (Wang et al. 2016a), (Vu et al. 2019)
Pseudorhypophila	CBS 149852[T]	OQ100832	OQ100090	OQ108867	-	(Charria-Girón et al. 2023)
Subramaniula	CBS 137114[T]	KP970641	KP862598	KP900667	KP900704	(Ahmed et al. 2016)
S. cristata	CBS 156.52[T]	KX976788	KX976690	KX976903	KX977038	(Wang et al. 2016a)
S. thielavioides	CBS 122.78[T]	KP970654	KP862597	KP900670	KP900708	(Ahmed et al. 2016)
Tengochaeta nigropilosa	CBS 639.83[T]	-	MZ334730	MZ342990	MZ343029	(Wang et al. 2022)
Tengochaeta bulbillosa	**CBS 151409[T]**	**PQ580716**	**PQ580714**	**PQ566605**	**PQ566606**	**Present study**

CBS: Westerdijk Fungal Biodiversity Institute, Utrecht, the Netherlands. [T] indicates type material

Alignment used in the phylogenetic study

>Tengochaeta_bulbillosa_CBS_151409

????????????????AACTCCC-AAACCATTGTGAACGTTACC-
TTTAACCGTTGCTTCGGCGGGCGGCCCC---------------------------
GGGACCACTCCCGGGGCCCCTGGGCCCC-----------
CTAGCGGGGCGCCCGCCGGAGGTCACC-TAACTCTTGATAATTT--
AAGGCCTCTCTGAGTCTTCTGTACTGAA-
TAAGTCAAAACTTTCAACAACGGATCTCTTGGTTCTGGCATCGATGAAGAACGCA
GCGAAATGCGATAAGTAATGTGAATTGCAGAATTCAGTGAATCATCGAATCTTTG
AACGCACATTGCGCCCGCCAGTATTCTGGCGGGCATGCCTGTCCGAGCGTCATTT
CAACCATCAAGCCCC-GGGCTTGTGTTGGGGACCTGCG----------
GCTGTCCGCAGGCCCTGAAAACCAGTGGCGGGCTCGCT-
GTCACCCCGAGCGTAGTAG---TATTATCTCGCTAAGGGCG-TGCTGCGGGTT--
CCGGCCGTTAAA-
CAGCCT??
??ttgaaatctggcctcggcccgagttgtaatttgc
ag-
aggaagctttaggcgcggtaccttctgagtcccctggaacggggcgccacagagggtgagagccccgtatagttggataccaagcct
gtgtaaagctcc-
ttcgacgagtcgagtagtttgggaatgctgctcaaaatgggaggtaaatttcttctaaagctaaataccggccagagaccgatagcgcac
aagtagagtgatcgaaagatgaaaagcactttgaaaagagggttaaatagcacgtgaaattgttgaaagggaagcgcttgtgaccaga
cttgcgccgggcggatcatccggtgttctcaccggtgcactccgcccggctcaggccagcatcggttctcgcgggggggataaaggtc
ctgggaacgtagctcctccgggagtgttatagcccagggcgcaatgcccccgcggggaccgaggttcgcgcatctgcaaggatgct
ggcgtaatggtcatcagcgacccgtcttgaaacacggaccaaggagtcaaggttttgcgcgagtgtttgggtgtaaaacccgcacgcg
taatgaaagtgaacgtaggtgagagcttcggcgcatcatcgaccgatcctgatgttttcggatggatttgagtaggagcgttaagccttg
gacccgaaagatggtgaactatgcttggatagggtgaagccagaggaaactctggtggaggctcgcagcgg-
ttctgacgtgcaaatcgatcgtcaaatctgagcatCTCGTGACGTTGGTGCAAGGGTTGAGGAGGAAGA
ACGTCATCTCGTTTGAGGTGTCCCTCGTCCGGGACATCCGCGACCGCGAGTTCAA
GATTTTCTCTGACGCGGGTCGCGTTATGAGGCCGCTGTTCACAGTGGAGCAAGAG
CATAACTCAGAGAGCGGTGCCGAGGTGGGCCAGTTGATTCTCAACAAGGACCAC
ATTAACCGGTTAGAGGCGGACAAGGAGTTGGGCAAATACCACCCCGACTACTGG
GGTTGGCAGGGATTGCTGAAGTCGGGCGCCATCGAGTACCTTGACGCCGAGGAA
GAGGAGACTGTCATGATTTCCATGACACCCGAGGACCTCGACAAGTTCCGGTACC
GTAAGATGGGCTTCATCGTGGAAGACAACTCGGGGC---
AAGGAAACAACAGGATCAAGACCAAACCGAACCCGACCACTCACATGTATACGC
ATTGTGAGATCCACCCCAGCATGCTGCTCGGTATCTGCGCCAGCATCATTCCCTTC
CCCGACCACAACCAGACGCGTC-CGGGGACC-TTTTTTTCTGGGCCCCTAAA---
CTACCC--CACCCATTGG-GACGTC-GTGG--TTCACCGGCTGGC--GACCCGA---
CCGACC-----TGAGCACCATGACGGCGCGACCATGAT--GGAGAGA-------ACAGG-
ATGCTGACAT--GGTTT--
CTACCACAGGTTCACCTTCAGACCGGCCAATGCGTAAGTTGGATCGATCC-
GAACACGGGGGGCCGT---------TCAGGTTG-TGC--GGGTG--GACTGACA-GCAAGCT-
CCCCAGGGTAACCAAATCGGTGCCGCTTTCTGGTATGCTCAACCTCAA-
CCTCAACCTCAAGTCTGC--AC-AGGCAAT---
CAAAACTAACTTCCCTTTCAGGCAGACCATCTCTGGCGAGCACGGCCTCGACAGC
AATGGCGTGTACGTAGCCGTCGCCGATTCCCGCC-CGATTAACCCC---

TCGCTCACCGCTCC-
GATAGGTACAACGGCACCTCCGAGCTTCAGCTCGAGCGCATGAACGTCTACTTCA
ACGAGGTGAGTTGGGACA-GGCATACTTT-CATGGTGTAGC-----TGAACGGCCAT-
GATCGCTGACAGT-GAAT----
GTCTCGCAGGCGTCCGGCAACAAGTATGTTCCTCGTGCCGTTCTCGTCGACTTGGA
GCCCGGCACCATGGATGCCGTCCGCGCTGGTCCCTTCGGCCAGCTCTTCCGCCCTG
ACAACTT

>Amesia_atrobrunnea_CBS_379_66

ATTACAGAGTTGC-AAAACTCCCTAAACCATTGTGAACGTTACCC-
TTAACCGTTGCTTCGGCGGGCGGCGCCAT---------------------------
GCCGCCCCTCGGCCCTTC---------------------CCGGGCGCCCGCCGGAGGATACCC-
AACTCTTGATATTTT--ATGGCCACTCTGAGTCTTCTGTACTGAA-
TAAGTCAAAACTTTCAACAACGGATCTCTTGGTTCTGGCATCGATGAAGAACGCA
GCGAAATGCGATAAGTAATGTGAATTGCAGAATTCAGTGAATCATCGAATCTTTG
AACGCACATTGCGCCCGCCAGTATTCTGGCGGGCATGCCTGTTCGAGCGTCATTT
CAACCATCAAGCCCCC-GGCTTGTGTTGGGGACCTGCG----------
GCTGCCCGCAGGCCCTGAAAAACAGTGGCGGGCTCGCT-
GTCACACCGAGCGTAGTAG--CATCATTCTCGCTCAGGGCG-TGCTGCGGGTT--
CCGGCCGTTAAA-
CGACCTTCATAACCCAAGGTTGACC???
??????????????????????????CCC-TAGTAACGGCG-
AGTGAAGCGGCAACAGCTCAAATTTGAAATCTGGCTTCGGCCCGAGTTGTAATTT
GTAG-
AGGAAGCTTTAGGCGCGGCACCTTCTGAGTCCCCTGGAACGGGGCGCCATAGAG
GGTGAGAGCCCCGTATAGTTGGATGCCTAGCCTGTGTAAAGCTCC-
TTCGACGAGTCGAGTAGTTTGGGAATGCTGCTCAAAATGGGAGGTAAATTTCTTC
TAAAGCTAAATACCGGCCAGAGACCGATAGCGCACAAGTAGAGTGATCGAAAGA
TGAAAAGCACTTTGAAAAGAGGGTTAAATAGCACGTGAAATTGTTGAAAGGGAA
GCGCTTGTGACCAGACTTGCGCCGGGCTGATCATCCGGTGTTCTCACCGGTGCAC
TCTGCCCGGCTCAGGCCAGCATCGGTTCTCGCGGGGGGATAAAGGCCCAGGGAAT
GTAGCTCCTCCGGGAGTGTTATAGCCCGGGGCGCAATGCCCTCGCGGGGACCGAG
GTTCGCGC-
TCTGCAAGGATGCTGGCGTAATGGTCATCAGCGACCCGTCTTGAAACACGGACCA
AGGAGTCAAGGTTTTGCGCGAGTGTTTGGGTGTAAAACCCGCACGCGTAATGAAA
GTGAACGTAGGTGAGAGCTTCGGCGCATCATCGACCGATCCTGATGTTTTCGGAT
GGATTTGAGTAAGAGCGTTAAGCCTTGGACCCGAAAGATGGTGAACTATGCTTGG
ATAGGGTGAAGCCAGAGGAAACTCTGGTGGAGGCTCGCAGCGG-
TTCTGACGTGCAAATCGATCGTCAAATCTGAGCATCTGGTCACACTGGTGCAGGG
TTTGCGGAGGAAGAACGTCATTTCGTTCGAGGTTTCGCTGGTTCGCGACATCCGC
GACCGCGAGTTCAAGATCTTCTCAGATGCCGGGCGCGTCATGAGGCCGCTGTTTA
CCGTCGAGCAGGAGCATGGTTCAGAGACCGGCGCCGAGATGGGCCAGCTCATCC
TCAACAAAGAGCATATTACACGGTTAGAAGCCGACAAGGAGCTGGGCAAGTACC
ATCCCGACTACTGGGGCTGGCAGGGCCTGCTGAAGTCGGGTGCCATCGAGTATCT
CGATGCCGAGGAGGAGGAGACAGCCATGATTTGCATGACGCCCGAGGATCTCGA
CAAGTTCCGGTACAGAAAGATGGGGTTCATCGTCGAAGACAACTCGGGTC---
AAGGTAACAACAGGATCAAGACGAAGCCGAACCCGGCCACCCACATGTACACGC

ACTGCGAGATCCATCCCAGCATGCTGCTCGGCATCTGCGCAAGCATCATCCCCTT
CCCCGACCACAACCAGACGCGTC--GGGAAGC---TTTTTTGGAACCCCTGAT---
CTACCC--CACACGTTGG-GAGGAC-CTCG-TTTC-CCAGCT------ATTCGA---CCGAC---
---CGAGAGCGATGATGGCACTGCCATGAT--AGAAAGA-------ACACA-
GTGCTGACGT--
TGCTTCTCTACTACAGGTTCACCTCCAGACCGGCCAATGCGTAAGTTGGATCGATT
T-GAGC-CCGACGACCGATGCCGTGTTTGTGATGG-TGC--GGGTGTAGACTGAC----
CAGCT-CTCCAGGGTAACCAAATTGGTGCCGCTTTCTGGTATGTTCGATGCCAA-
CATCAAGCTCTTGCGCGTC-TG-AGGCAAC---
CAAGACTGACTTCCATCACAGGCAGACCATCGCCGGCGAGCACGGCCTCGACAG
CAATGGCGTGTACGTGACTGTCGCCGACCCAGACT-CTGATAATCCC---
TCGCTCACCGCTAC-
GATAGGTACAACGGCACTTCGGAGCTCCAGCTCGAGCGCATGAACGTCTACTTCA
ATGAGGTGAGTCGGGCC--CGTACACCTT-CATCAGATATC-----TTGAATGCTGC-
GGTCGCTGACAAC-ATAT----
TCTTGGCAGGCTTCCGGCAACAAGTATGTCCCTCGTGCGGTCCTTGTCGACTTGGA
GCCCGGCACCATGGATGCCGTCCGCGTTGGTCCCTTCGGCCAGCTCTTCCGCCCTG
ACAACTT

>Amesia_nigricolor_CBS_600_66

ATTACAGAGTTGC-AAAACTCCCTAAACCATTGTGAACGTTACC-
TTTAACCGTTGCTTCGGCGGGCGGCGCTCT----------------------------
GCCGCCCCTCGGCCCTGC----------------------CCGGGCGCCCGCCGGAGGATACCC-
AACTCTTGATACTTT-GATGGCCTCTCTGAGTCTTCTGTACTGAA-
TAAGTCAAAACTTTCAACAACGGATCTCTTGGTTCTGGCATCGATGAAGAACGCA
GCGAAATGCGATAAGTAATGTGAATTGCAGAATTCAGTGAATCATCGAATCTTTG
AACGCACATTGCGCCCGCCAGTATTCTGGCGGGCATGCCTGTTCGAGCGTCATTT
CAACCATCAAGCCCAC-GGCTTGTGTTGGGGACCTGCG----------
GCTGCCCGCAGGCCCTGAAAACCAGTGGCGGGCTCGCT-
GTCACACCGAGCGTAGTAG--CATCATTCTCGCTCAGGGCG-TGCTGCGGGTT--
CCGGCCGTTAAA-
CGACCTTCATAACCCAAGGTTGACCTCGGATCAGGTAGGAAGACCCGCTGAACTT
AAGCATATCAATAAGCGGAGGAAAAGAAACCAACAGGGATTGCCC-
TAGTAACGGCG-
AGTGAAGCGGCAACAGCTCAAATTTGAAATCTGGCTTCGGCCCGAGTTGTAATTT
GCAG-
AGGAAGCTTTAGGCGCGGCACCTTCTGAGTCCCCTGGAACGGGGCGCCATAGAG
GGTGAGAGCCCCGTATAGTTGGATGCCTAGCCTGTGTAAAGCTCC-
TTCGACGAGTCGAGTAGTTTGGGAATGCTGCTCAAAATGGGAGGTAAATTTCTTC
TAAAGCTAAATACCGGCCAGAGACCGATAGCGCACAAGTAGAGTGATCGAAAGA
TGAAAAGCACTTTGAAAAGAGGGTTAAATAGCACGTGAAATTGTTGAAAGGGAA
GCGCTTGTGACCAGACTTGCGCCGGGCTGATCATCCGGTGTTCTCACCGGTGCAC
TCTGCCCGGCTCAGGCCAGCATCGGTTCTCGCGGGGGGATAAAGGTTCCGGGAAT
GTAGCTCCTCCGGGAGTGTTATAGCCCGGGGCGTAATGCCCTCGCGGGGACCGAG
GTTCGCGC-
TCTGCAAGGATGCTGGCGTAATGGTCATCAGCGACCCGTCTTGAAACACGGACCA
AGGAGTCAAGGTTTTGCGCGAGTGTTTGGGTGTAAAACCCGCACGCGTAATGAAA

GTGAACGTAGGTGAGAGCTTCGGCGCATCATCGACCGATCCTGATGTTTTCGGAT
GGATTTGAGTAAGAGCGTTAAGCCTTGGACCCGAAAGATGGTGAACTATGCTTGG
ATAGGGTGAAGCCAGAGGAAACTCTGGTGGAGGCTCGCAGCGG-
TTCTGACGTGCAAATCGATCGTCAAATCTGAGCATCTGGTCACATTGGTGCAAGG
TCTGCGGAGGAAGAACGTCATTTCGTTCGAGGTGTCGTTGGTTCGCGACATCCGC
GACCGCGAGTTCAAGATCTTTTCCGATGCGGGGCGCGTCATGAGGCCGCTATTTA
CCGTCGAGCAGGAGCACGGTTCAGAGACCGGCGCCGAAATGGGGCAGCTCATCC
TCAACAAGGAACATATTACACGGTTAGAAGCGGACAAGGAGCTGGGCAAATACC
ATCCCGACTACTGGGGCTGGCAGGGCCTGCTTAAGTCGGGCGCCATCGAGTATCT
CGATGCCGAGGAGGAGGAGACGGCCATGATCTGCATGACGCCCGAGGACCTCGA
CAAGTTCCGGTACAGAAAGATGGGGTTCATCGTCGAAGACAACTCGGGTC---
AAGGTAACAACAGGATCAAGACGAAGCCGAACCCTGCCACCCACATGTACACGC
ACTGCGAGATCCATCCCAGCATGCTGCTCGGCATCTGCGCCAGCATCATCCCCTTC
CCCGACCACAACCAGACGCGTC--GAGAAGC---TTTTTTTGGGCCCCTGAT---
CTACCC--CACATATTGG-GACGAC-CTCG-TTTGACCAGCT------ACCCGA---CCGAC-
-----CGAGAGCGATGACGGCGCGGCCGCGAT--GCAAAGG-------ACATG-
GTGCTGACAT--
TGCTTCTCTACTATAGGTTCACCTCCAGACCGGCCAATGCGTAAGTTGGATCGATT
C-GAGC-CCGACGACCGATACCA----TGTGCCAG-TGC--AGGTGTAGACTGACA-
ACAAGCT-
CTCCAGGGTAACCAAATCGGTGCTGCTTTCTGGTATGTTCGATGCCAA-
CATCAACCGCGTGCGTGTC-AA-AAGCAAT---
CGAGACTGACTTCCATCACAGGCAGACCATCTCTGGCGAGCACGGCCTCGACAGC
AATGGCGTGTACGTGACTGTCGGCGACCCAAATT-CCGATAATCGC---
TCGCTCACCGGTAC-
GATAGGTACAACGGCACCTCGGAGCTCCAGCTCGAGCGCATGAACGTCTACTTCA
ACGAGGTGAGTTGGGCC--TGTCCACCTT-CATCAGTTATC-----TTGAAGGCTGC-
GGTTGCTGACAAC-ATAT----
TCTTCGCAGGCTTCCGGCAACAAGTATGTCCCTCGTGCCGTCCTCGTCGACTTGGA
GCCCGGCACCATGGATGCCGTCCGCGCTGGTCCCTTCGGCCAGCTCTTTCGCCCTG
ACAACTT

>Botryotrichum_piluliferum_CBS_654_79

ATTACAGAGTTGC-
AAAACTCCCTAAACCATTGTGAACGTTACCTTCAAACCGTTGCTTCGGCGGGCGG-
CCCGGGTC--------------------------CGCCCGGTGCCCCCTGGCCCCCTA--------------
GCGGGGCGCCCGCCGGAGGAAAACCCAACTCTTGATTATT---
ATGGCCTCTCTGAGTCTTCTGTACTGAA-
TAAGTCAAAACTTTCAACAACGGATCTCTTGGTTCTGGCATCGATGAAGAACGCA
GCGAAATGCGATAAGTAATGTGAATTGCAGAATTCAGTGAATCATCGAATCTTTG
AACGCACATTGCGCCCGCCAGTATTCTGGCGGGCATGCCTGTTCGAGCGTCATTT
CAACCATCAAGCCCC-GCGCTTGTGTTGGGGACCTGCG----------GCTG-
CCGCAGGCCCTGAAAACCAGTGGCGGGCTCGCT-GTCACACCGGGCGTAGTAG--
ATTTTATCTCGCTCAGGGCG-TGCTGCGGGTT--CCGGCCGTTAAAAAGCCTTT-
TTTACCCAAGGTTGACCTCGGATCAGGTAGGAATACCCGCTGAACTTAAGCATAT
CAATAAGCGGAGGAAAAGAAACCAACAGGGATTGCCT-CAGTAACGGCG-
AGTGAAGCGGCAACAGCTCAAATTTGAAATCTGGCTTCGGCCCGAGTTGTAATTT

GCAG-
AGGAAGCTTTAGGCGCGGCACCATCTGAGTCCCCTGGAACGGGGCGCCACAGAG
GGTGAGAGCCCCGTATAGATGGACGCCTAGCCTGTGTAAAGCTCC-
TTCGACGAGTCGAGTAGTTTGGGAATGCTGCTCAAAATGGGAGGTAAATTTCTTC
TAAAGCTAAATACCGGCCAGAGACCGATAGCGCACAAGTAGAGTGATCGAAAGA
TGAAAAGCACTTTGAAAAGAGGGTTAAATAGCACGTGAAATTGTTGAAAGGGAA
GCGCTTGTGACCAGACTTGCGCCGGGCGGATCATCCGGTGTTCTCACCGGTGCAC
TCCGCCCGGCTCAGGCCAGCATCGGTTCTCGTGGGGGATAAAGGCACCGGGAA
CGTAGCTCCTCCGGGAGTGTTATAGCCCGGGGCGTAATGCCCTCGCGGGACCGA
GGACCGCGCATCTGCAAGGATG???
???
???
??CTGGTCACCCTG
GTTCAGGGTTTGCGGAGGAAGAACGTCATCTCGTTCGAGGTTTCGCTCGTCCGGG
ACATTCGCGACCGCGAGTTCAAGATTTTCTCCGACGCGGGCCGTGTCATGAGGCC
GTTGTTCACGGTAGAACAAGAGAAGAACGGAGAGAGCGGCGCAGAAATGGGGC
AGCTTATTCTCAACAAGGAGCACATTACGAGATTGGAGGCGGACAAGGAGTTGG
GCAAGTATCACCCCGACTACTGGGGCTGGCAGGGCTTGTTGAAGTCGGGCGCTAT
CGAGTACCTCGATGCCGAAGAAGAGGAGACGGTCATGATCAGCATGACTCCCGA
GGACCTCGACAAGTTCCGTTACCGCAAGATGGGGTTCATCGTTGAAGACAACTCT
GGCC---
AAGGTAATAACAGGATCAAGACAAAGCCGAACCCGGCCACGCACATGTACACCC
ACTGCGAGATCCACCCCAGCATGTTGCTCGGCATCTGCGCCAGCATCATCCCCTTC
CCCGACCACAACCAGACGCGTC-TGGGATGC----TTTTTGGGGACCCTGAT---
CTACCC--CACACACTGG-GACGAC-GTTC-CCCGACCA----------CCCGA---CCGAC-----
GCGAGAGCGGTAGCGGCGCTGCCATGAT--GAAATGT-------ACACG-
ATGCTGACTT--
TATCTTTCTCCTACAGGTCCACCTCCAGACCGGCCAATGCGTAAGTTGGATCGACT
C-GAAC-ACGGCGACCGA---------CCGGATGG-GGC--GGATG--
GACTGACAAGCAACCT-
CTCTAGGGTAACCAAATCGGTGCCGCTTTCTGGTATGTCCGGCCTCAA-
CATCAAGCAATGACGTGTC-AA-GACGGAT---
CAAGACTGACTTCCCCTCCAGGCAGACCATTTCCGGCGAGCACGGCCTCGACGCC
AATGGCGTGTATGTGACCGCCGCCGATTCCCGGC-CGATGAATCCC---
CCGCTCACCGCTTC-
GATAGGTACAACGGCACCTCCGAGCTCCAGCTCGAGCGCATGAACGTCTACTTCA
ACGAGGTGAGTCGGGCC--CGTACGGCCT---TGAGGCAGC-----CAGAC-------
GGTCGCTGACGAC-AGAT----
ATTCTACAGGCGGCAGGCAACAAGTATGTCCCTCGTGCCGTCCTTGTCGACTTGG
AGCCCGGCACCATGGATGCCGTCCGCGCCGGTCCTTTCGGCCAGCTCTTTCGCCCC
GACAACTT

>Botryotrichum_vitellinum_CBS_180_84

?TTACAGAGTTGC-
AAAACTCCCTAAACCATTGTGAACGTTACCTTCAAACCGTTGCTTCGGCGGGCGG
CGCCGGGTCCGCCC-------------------GGCGCCCCTCGGCCCCCCCTCGCG---------------
GGGAGCGCCCGCCGGAGGAAACCC-AACTCTTGATATAT---

ATGGCCTCTCTGAGTCTTCTGTACTGAA-
TAAGTCAAAACTTTCAACAACGGATCTCTTGGTTCTGGCATCGATGAAGAACGCA
GCGAAATGCGATAAGTAATGTGAATTGCAGAATTCAGTGAATCATCGAATCTTTG
AACGCACATTGCGCCCGCCAGTATTCTGGCGGGCATGCCTGTTCGAGCGTCATTT
CAACCATCAAGCCCCCGGGCTTGTGTTGGGGACCTGCG----------GCTG-
CCGCAGGCCCTGAAAACCAGTGGCGGGCTCGCT-GTCACACCGGGCGTAGTAG---
TTTTATCTCGCTCAGGGCG-TGCTGCGGGTT--CCGGCCGTTAAA-AGCCTTT-
TTTACCCAAGGTTGACCTCGGATCAGGTAGGAATACCCGCTGAACTTAAGCATAT
CAATAAGCGGAGGAAAAGAAACCAACAGGGATTGCCC-TAGTAACGGCG-
AGTGAAGCGGCAACAGCTCAAATTTGAAATCTGGCTTCGGCCCGAGTTGTAATTT
GCAG-
AGGAAGCTTTAGGCGCGGCACCATCTGAGTCCCCTGGAACGGGGCGCCATAGAG
GGTGAGAGCCCCGTAGAGATGGACGCCTAGCCTGTGTAAAGCTCC-
TTCGACGAGTCGAGTAGTTTGGGAATGCTGCTCAAAATGGGAGGTAAATTTCTTC
TAAAGCTAAATACCGGCCAGAGACCGATAGCGCACAAGTAGAGTGATCGAAAGA
TGAAAAGCACTTTGAAAAGAGGGTTAAATAGCACGTGAAATTGTTGAAAGGGAA
GCGCTTGTGACCAGACTTGCGCCGGGCGGATCATCCGGTGTTCTCACCGGTGCAC
TCCGCCCGGCTCAGGCCAGCATCGGTTCTCGCGGGGGGATAAAGGCACCGGGAA
CGTAGCTCCTCCGGGAGTGTTATAGCCCGGGGCGTAATGCCCTCGCGGGGACCGA
GGACCGCGCATCTGCAAGGATG??
???
???
???CTAGTCACTCTA
GTGCAAGGGCTGCGGAGGAAGAATGTCATCTCGTTTGAGGTTTCGCTCGTCCGGG
ACATCCGCGACCGCGAGTTCAAGATCTTCTCCGATGCGGGTCGTGTCATGAGGCC
GCTGTTCACCGTGGAGCAGGAAAAGAACGGAGACAGCGGCGCCGAGATGGGCCA
ACTGATCCTCAACAAGGAGCACATTACGAGGCTGGAGGCGGATAAGGAGTTGGG
CAAGTACCACCCCGACTACTGGGGCTGGCAAGGGCTGCTGAAGTCGGGTGCCATC
GAGTATCTCGACGCCGAGGAGGAGGAGACAGTCATGATCAGCATGACTCCCGAG
GATCTCGACAAGTTCCGTTACCGCAAGATGGGGTTCATCGTGGAAGACAACTCTG
GTC---
AAGGAAACAACAGAATCAAGACGAAGCCAAACCCCGCGACGCACATGTACACGC
ACTGCGAGATCCACCCCAGCATGTTGCTCGGCATCTGCGCCAGCATCATCCCGTT
CCCCGACCACAACCAGACGCGTC-TGGGAAGC---TTTTTCTGGGCCCCTGAT---
CTACCC--CACACATTTG-GACGACAGTCG-TTCCACCAGCTCCCCGACCCCGA---
CCGACGGAGGGCGATGTCGATAGCGGCGCTGCCATGAT--GGAAAGG------
AATATG-TCGCTGACAC--
TGTCTCTCTGCTACAGGTTCACCTCCAGACCGGCCAATGCGTAAGTTGGATCGAT
GC-GAAC-ACGGCGACCGA---------TCGGGTGG-TGC--GGGTG--GACTGACA-
GCAACCT-CTCCAGGGTAACCAAATTGGTGCCGCTTTCTGGTATGTTCGACCTCAA-
CATGAAACACTGACATGCCGAA-AAACGAT---
CAAGACTGACTTCCCCTCCAGGCAGACCATTTCCGGCGAGCACGGCCTTGACAGC
AGTGGCGTGTACGTGACCGTCGCCGATCCCGGC--CAACGAATCCC---
TCGCTCACCGCTAC-
GATAGGTACAATGGCACCTCCGAGCTCCAGCTCGAGCGCATGAACGTCTACTTCA
ACGAGGTGAGTCGGGCC--CAGACACCTT-AATCGAGCAAT-----CAAACGGCCGC-
GGTCGCTAACAACCACAC----

TTTCTGCAGGCGTCCGGCAACAAGTATGTCCCTCGTGCCGTCCTGGTCGACTTGGA
GCCCGGCACCATGGATGCCGTCCGCGCCGGTCCCTTCGGCCAGCTCTTCCGCCCC
GACAACTT

>Chaetomium_cochliodes_CBS_155_52

ATTACAGAGTTGC-AAAACTCCCTAAACCATTGTGAACGTTACC--
TAAACCGTTGCTTCGGCGGGCGGCGCCGGGGTTTACCCCCC---------------
GGGCGCCCCTGGGCCCCACC---------------------GCGGGCGCCCGCCGGAGGTCACC-
AAACTCTTGATAATTT--ATGGCCTCTCTGAGTCTTCTGTACTGAA-
TAAGTCAAAACTTTCAACAACGGATCTCTTGGTTCTGGCATCGATGAAGAACGCA
GCGAAATGCGATAAGTAATGTGAATTGCAGAATTCAGTGAATCATCGAATCTTTG
AACGCACATTGCGCCCGCCAGTATTCTGGCGGGCATGCCTGTTCGAGCGTCATTT
CAACCATCAAGCCCC-GGGCTTGTGTTGGGGACCTGCG----------GCTG-
CCGCAGGCCCTGAAAAGCAGTGGCGGGCTCGCT-GTCACACCGAGCGTAGTAG--
CATACATCTCGCTCTGGGCG-TGCTGCGGGTT--CCGGCCGTTAAACCACCTTT--
TAACCCAAGGTTGACCTCGGATCAGGTAGGAAGACCCGCTGAACTTAAGCATATC
AATAAGCGGAGGAAAAGAAACCAACAGGGATTGCCC-TAGTAACGGCG-
AGTGAAGCGGCAACAGCTCAAATTTGAAATCTGGCTTCGGCCCGAGTTGTAATTT
GCAG-
AGGAAGCTTTAGGCGCGGCACTTTCTGAGTCCCCTGGAACGGGGCGCCATAGAGG
GTGAGAGCCCCGTATAGTTAGATGCCTAGCCTGTGTAAAGCTCC-
TTCGACGAGTCGAGTAGTTTGGGAATGCTGCTCAAAATGGGAGGTAAATTTCTTC
TAAAGCTAAATACCGGCCAGAGACCGATAGCGCACAAGTAGAGTGATCGAAAGA
TGAAAAGCACTTTGAAAAGAGGGTTAAATAGCACGTGAAATTGTTGAAAGGGAA
GCGCTTGTGACCAGACTTGCGCCGGGCAGATCATCCGGTGTTCTCACCGGTGCAC
TCTGCCCGGCTCAGGCCAGCATCGGTTCTCGCGGGGGGATAAAGGCCCTGGGAAC
GTAGCTCCTCCGGGAGTGTTATAGCCCGGGGCGTAATGCCCTCGCGGGGACCGAG
GTTCGCGCATCTGCAAGGATGCTGGCGTAATGGTCATCAGCGACCCGTCTTGAAA
CACGGACCAAGGAGTCAAGGTTTTGCGCGAGTGTTTGGGTGTAAAACCCGCACGC
GTAATGAAAGTGAACGTAGGTGAGAGCTTCGGCGCATCATCGACCGATCCTGATG
TTTTCGGATGGATTTGAGTAGGAGCGTTAAGCCTTGGACCCGAAAGATGGTGAAC
TATGCTTGGATAGGGTGAAGCCAGAGGAAACTCTGGTGGAGGCTCGCAGCGG-
TTCTGACGTGCAAATCGATCGTCAAATCTGAGCATCTCGTCACGCTGGTCCAGGG
GCTGCGGAGAAAGAACGTCATCTCGTTCGAGGTGTCGCTCGTTAGAGATATCCGC
GACCGCGAGTTCAAGATCTTTTCAGATGCAGGCCGCGTCATGAGACCGCTTTTCA
CAGTGGAGCAAGAACCAAACGGCGAGAGCGGCGCTGAGATGGGCGCGCTGATTC
TCAACAAGGAGCACGTTGGGAAGTTGAAGTTGGACACCGAGTACGGCAAATACC
ATCCGAACTACTGGGGCTGGCAAGGCTTGTTGAAATCGGGCGCTATTGAATATCT
TGACGCTGAGGAAGAGGAGACGGTGATGATCTGCATGACCCCCCAGGATCTCGA
CCAGTTCCGTGCCCGCAAGCAGGGAAGATTCGAGCAGGATAACTCGGGGT---
TGGGCAATAACCGCATCAAGACGAAGCCGAACCCGACGACTCACATGTACACGC
ATTGCGAGATCCATCCCAGCATGCTCCTCGGCATCTGCGCGAGCATCATCCCCTTC
CCCGACCACAACCAGACGCGTCTTGGGAAGC-TTTTTTTTTGGGCCCCTGAA---
CTACCC--CACCCAT-GG-GACGACCCTCA-TTTCCACAGCT---------------------------
CCGAGCGATGCCAACGCGACGATGAT--GCGAAGA------ATCATG-ATGCTGACTG---
CTTTTCGTACTATAGGTCCACCTCCAGACCGGCCAGTGCGTAAGTTAGAGCGACT
T-GAGT-ATTACGACCGA---------TC-GGCCG-GGC--AGAAT--AACTGACA-

GCAAGCT-
CTTTAGGGTAACCAAATCGGTGCCGCTTTCTGGTATGTCAACCAAGCA-
AGGCAAACACTCGTGGTC--GA-TGGCAAT---CGAGACTAACTT-
CTCTTCAGGCAGACCATCTCTGGCGAGCACGGCCTCGACAGCAATGGCGTGTATG
TCGCCGTAGAATAGTCCCCAG-TAGATATACCC---CCGCTCACCGCTTC-
GATAGGTACAACGGCACCTCTGAGCTCCAGCTCGAGCGCATGAACGTCTACTTCA
ACGAGGTCAGTAGGGTC--AAACACCGT-----TACACAAT-----CAAATGAAGGT---
GTGCTCACAGT---------
GTTTCATAGGCTTCCGGCAACAAGTATGTTCCCCGTGCCGTTCTCGTCGACTTGGA
GCCCGGCACCATGGATGCCGTCCGTGCCGGCCCCTTCGGCCAGCTCTTCCGCCCG
GACAACTT

>Chaetomium_globosum_CBS_160_62

ATTACAGAGTTGC-AAAACTCCCTAAACCATTGTGAACGTTACC--
TATACCGTTGCTTCGGCGGGCGGCCCCGGGGTTTACCCCCC--------------
GGGCGCCCCTGGGCCCCACC--------------------GCGGGCGCCCGCCGGAGGTCACC-
AAACTCTTGATAATTT--ATGGCCTCTCTGAGTCTTCTGTACTGAA-
TAAGTCAAAACTTTCAACAACGGATCTCTTGGTTCTGGCATCGATGAAGAACGCA
GCGAAATGCGATAAGTAATGTGAATTGCAGAATTCAGTGAATCATCGAATCTTTG
AACGCACATTGCGCCCGCCAGCATTCTGGCGGGCATGCCTGTTCGAGCGTCATTT
CAACCATCAAGCCCCCGGGCTTGTGTTGGGGACCTGCG----------GCTG-
CCGCAGGCCCTGAAAAGCAGTGGCGGGCTCGCT-GTCGCACCGAGCGTAGTAG--
CATACATCTCGCTCTGGTCG-CGCCGCGGGTT--CCGGCCGTTAAACCACCTTT--
TAACCCAAGGTTGACCTCGGATCAGGTAGGAAGACCCGCTGAACTTAAGCATATC
AATAAGCGGAGGAAAAGAAACCAACAGGGATTGCCC-TAGTAACGGCG-
AGTGAAGCGGCAACAGCTCAAATTTGAAATCTGGCTTCGGCCCGAGTTGTAATTT
GCAG-
AGGAAGCTTTAGGCGCGGCACCTTCTGAGTCCCCTGGAACGGGGCGCCATAGAG
GGTGAGAGCCCCGTATAGTTGGATGCCTAGCCTGTGTAAAGCTCC-
TTCGACGAGTCGAGTAGTTTGGGAATGCTGCTCAAAATGGGAGGTAAATTTCTTC
TAAAGCTAAATACCGGCCAGAGACCGATAGCGCACAAGTAGAGTGATCGAAAGA
TGAAAAGCACTTTGAAAAGAGGGTTAAATAGCACGTGAAATTGTTGAAAGGGAA
GCGCTTGTGACCAGACTTGCGCCGGGCGGATCATCCGGTGTTCTCACCGGTGCAC
TCCGCCCGGCTCAGGCCAGCATCGGTTCTCGCGGGGGGATAAAGGTCCTGGGAAC
GTAGCTCCTCCGGGAGTGTTATAGCCCGGGGCGTAATGCCCTCGCGGGGACCGAG
GTTCGCGCATCTGCAAGGATGCTGGCGTAATGGTCATCAGCGACCCGTCTTGAAA
CACGGACCAAGGAGTCAAGGTTTTGCGCGAGTGTTTGGGTGTAAAACCCGCACGC
GTAATGAAAGTGAACGTAGGTGAGAGCTTCGGCGCATCATCGACCGATCCTGATG
TTTTCGGATGGATTTGAGTAGGAGCGTTAAGCCTTGGACCCGAAAGATGGTGAAC
TATGCTTGGATAGGGTGAAGCCAGAGGAAACTCTGGTGGAGGCTCGCAGCGG-
TTCTGACGTGCAAATCGATCGTCAAATCTGAGCATCTTGTCACGCTGGTTCAGGG
GCTGCGGAGAAAGAACGTTATCTCGTTTGAGGTTTCGCTCGTTAGAGACATCCGC
GACCGTGAGTTCAAGATCTTTTCAGATGCGGGTCGGGTGATGAGGCCGCTGTTCA
CGGTGGAGCAAGAACCGAATGGCGAGAGCGGCGCTGAGATGGGCGCACTGATCC
TGAACAAGGATCATATTGGGCGCCTGAAGATGGACGCAGAGCTGGGCAAATACC
ACCCGGACTACTGGGGCTGGCAAGGCCTGTTGAAGTCGGGCGCTATTGAGTATCT
TGATGCTGAGGAGGAGGAGACGGTCATGATCTGCATGACCCCCCAGGATCTTGAT

CAGTTCCGTGCCCGCAAGATGGGAAGGATCGAGCCGGACAACTCCGGGT---
TGGGCAATAACCGGATCAAGACGAAACCAAATCCGACAACTCACATGTACACGC
ACTGCGAGATCCATCCGAGCATGCTCCTCGGCATCTGCGCAAGCATCATCCCCTTT
CCTGATCATAACCAACTGCGTC-TGGGAAGC-TTTTTTTTGGGCCTCTGAA---
CTACCC--CACTCATCGG-GACGACCCTCA-TTTCCACAGCT-------------------------
CCTGACGGATAGCAACACGACGATGAT--GCGAAGC------TTGATG-ATGCTGACTG-
-
CTTTTTCGTACTATAGGTTCACCTCCAGACCGGCCAGTGCGTAAGTTGGACCGAAT
T-GAAC-ATTACGACCGA---------CC-GGCCG-CGC--AGGAT--AACTGACA-
TGGAGCT-
CTCTAGGGTAACCAAATCGGTGCCGCTTTCTGGTACGTCCAAGCAAAG----
CAAACACTCTTGGCT--GA-TGACAAT---CGAGACTGACTT-
CTTTTCAGGCAGACCATCTCTGGCGAGCACGGCCTCGACAGCAATGGCGTGTATG
TGGGCATGACAGTTCCCAACC---GATAAATCC---CCGCTCACCGCTTC-
GATAGGTACAACGGCACCTCCGAGCTCCAGCTCGAGCGTATGAACGTGTACTTCA
ACGAGGTCAGTCGGGTC--AAATAATTT-----TACACGAC-----CGAGTGATGGC---
GTGCTCATAGT---------
ATTATACAGGCTTCCGGCAACAAGTATGTTCCTCGCGCTGTCCTCGTCGACTTGGA
GCCCGGCACCATGGATGCCGTCCGTGCCGGCCCCTTCGGCCAGCTCTTCCGCCCG
GACAACTT

>Corynascella_humicola_CBS_337_72

ATTACAGAGTTGA-AAGACTCCCCATACCATCGCGAACGTTACCCTT-
AACCGTTGCTTCGGCGGGCGGTCTCCTCTCCCCCCCCCTTCTGGGGGCGGGTTGGG
AAGCCCCTCGGCCCTCCCTTTCCCTTCGCGGGGTTGGGGCGGGGCGCCCGCCGGA
GGTCACGCAAACTCTTGATACCTGAAACGGCCTCTCTGAGTATTCTGTACTTAATT
AAGTCAAAACTTTCAACAACGGATCTCTTGGTTCTGGCATCGATGAAGAACGCAG
CGAAATGCGATAAGTAATGTGAATTGCAGAATTCAGTGAATCATCGAATCTTTGA
ACGCACATTGCGCCCGCCAGCATTCTGGCGGGCATGCCTGTTCGAGCGTCATTTC
AACCATCAAGCCCCCGGGCTTGTGTTGGGGACCTGCGCGGCTCTTTTCTCGCCCGC
AGGCCCTGAAATGCAGTGGCGGGCTCGCTAGTCACACCGAGCGTAGTAGCATTTT
TGTCTCGCTCAGGGCGTTGCTGCGGGTTTCCCGGCCGTTAAACACCCACCATTT-
CTCAAGGTTGACCTCGGATCAGGTAGGAAGACCCGCTGAACTTAAGCATATCAAT
AAGCGGAGGAAAAGAAACCAACAGGGATTGCCCTTAGTAACGGCGAAGTGAAGC
GGCAACAGCTCAAATTTGAAATCTGGCCTCGGCCCGAGTTGTAATTTGCAGAAGG
AAGCTTTGGGCGCGGCGCCTTCCGAGTCCCCTGGAACGGGGCGCCATAGAGGGTG
AGAGCCCCGTATGGTTGGACGCCTAGCCTGTGTAAAGCTCC-
TTCGACGAGTCGAGTAGTTTGGGAATGCTGCTCAAAATGGGAGGTAAATTTCTTC
TAAAGCTAAATACCGGCCAGAGACCGATAGCGCACAAGTAGAGTGATCGAAAGA
TGAAAAGCACTTTGAAAAGAGGGTTAAATAGCACGTGAAATTGTTGAAAGGGAA
GCGCTTGTGACCAGACTTGCGCCGGGCTGATCATCCGGTGTTCTCACCGGTGCAC
TCTGCCCGGCTCAGGCCAGCATCGGTTCTCGCGCGGGGACAAAGGTCCCGGGAAT
GTAGCTCCTCCGGGAGTGTTATAGCCCGGGGCGCAATGCCCGCGCGGGGACCGA
GGCTCGCGC-
TCTGCAAGGATGCTGGCGTAATGGTCATCAGCGACCCGTCTTGAAACACGGACCA
AGGAGTCAAGGTTTTGCGCGAGTGTTTGGGTGTCAAACCCGCACGCGTAATGAAA
GTGAACGTAGGTGAGAGCTTCGGCGCATCATCGACCGATCCTGATGTATTCGGAT

GGATTTGAGTAGGAGCGTTAAGCCTTGGACCCGAAAGATGGTGAACTATGCTTGG
ATAGGGTGAAGCCAGAGGAAACTCTGGTGGAGGCTCGCAGCGGTTTCTGACGTG
CAAATCGATCGTCAAATCTGAGCATCTGGTCTCACTGGTGCAGGGTTTGCGGAGA
AAGAATGTCATTTCGTTTGAGGTTTCGCTTGTCCGGGACATTCGCGATCGCGAGTT
CAAGATCTTTTCGGATGCTGGCCGCGTCATGAGGCCTCTGTTTACTGTGGAACAA
GAGGACAACAGCGACACCGGCGCCGAGAAAGGCCAGCTGGTCCTAAATAAGGAG
CACATCCAACGGCTAGAGAGGGATAAGGAGCTCGGCAAATACCACCCCGACTAT
TGGGGCTGGCCTGGGCTGTTGAAGTCGGGCGCGATTGAATACCTCGACGCTGAGG
AAGAGGAAACGGCCATGATCAGCATGAGTCCCGAGGATCTCGACATGTTCCGGCT
CCGCAAGCTGGGCTTCACCGTCGAAGACCATTCCGGCG---
AAGGCAATAGCCGAATCAGGACCAAGCCGAACCCGGCGACTCACATGTACACGC
ACTGCGAGATCCATCCCAGCATGCTTCTCGGCATCTGCGCGAGTATTATCCCATTC
CCCGACCACAACCAG??GCGTCTCGGGAAGCTATATTTTTGGGCCCCTGAC--
CTTACCC--CACACTTTTT-GAACAA-TACG--CGCCCAAGCT------CCCAGA---
CCGACT----GGAAAGGCGATGCCGATGCTCTCTTGAT--GGGATCG-------ACACG-
ATACTAACTA--
TGGTTCTCTGCTATAGGTTCACCTTCAGACTGGCCAATGCGTAAGTTCGACCGATT
C-GAGCGTCGGCGGCCTTATCA---GGTGGTGTGG-TGC--AGGTG--GACTGACA-
ACAACCCGATGCAGGGTAACCAAATTGGTGCCGCTTTCTGGTATGGCCATCCTCA
A-GATTCAGCACCATCGCCCA-AA-AGCCACT---
TCTTGCTGACTTATTTGACAGGCAGACTATCTCCGGCGAGCACGGCCTCGACGGC
AATGGCGTGTACGTGGCTGCTGGCGATTCCCGGT-CGGTTAATCCC---
TCGCTCACCGTCCC-
GATAGGTATAATGGCACCTCCGAGCTCCAGCTCGAGCGCATGAACGTCTACTTCA
ACGAGGTAAGTTCGGAT--CCCAGATCTC---CTGAGCGGT-----TGGAGTGGGAT-
GTGCGCTG------AAAT----
CTGGTGCAGGCGTCCGGCAACAAGTATGTCCCTCGTGCCGTTCTGGTCGACTTGG
AGCCCGGCACGATGGATGCCGTCCGCGCTGGTCCCTTCGGCCAGCTCTTCCGCCC
CGACAACTT

>Amesia_hispanica_FMR12004

ATTACAGAGTTGC-AAAACTCCCTAAACCATTGTGAACCTTACC-
TTTAACCGTTGCTTCGGCGGGCGGCGCTCT----------------------------
GCCGCCCCTCGGCCCTGC----------------------CTGGGCGCCCGCCGGAGGATACCC-
AACTCTTGATTCTTG--ATGGCCTCTCTGAGTCTTCTGTACTGAA-
TAAGTCAAAACTTTCAACAACGGATCTCTTGGTTCTGGCATCGATGAAGAACGCA
GCGAAATGCGATAAGTAATGTGAATTGCAGAATTCAGTGAATCATCGAATCTTTG
AACGCACATTGCGCCCGCCAGTATTCTGGCGGGCATGCCTGTTCGAGCGTCATTT
CAACCATCAAGCCCAC-GGCTTGTGTTGGGGACCTGCG----------
GCTGCCCGCAGGCCCTGAAAACCAGTGGCGGGCTCGCT-
GTCACACCGAGCGTAGTAG--CATCATTCTCGCTCAGGGCG-TGCTGCGGGTT--
CCGGCCGTTAAA-
CGACCTTCATAACCCAAGGTTGACCTCGGATCAGGTAGGAAGACCCGCTGAACTT
AA???????????????????AAAGAAACCAACAGGGATTGCCC-TAGTAACGGCG-
AGTGAAGCGGCAACAGCTCAAATTTGAAATCTGGCTTCGGCCCGAGTTGTAATTT
GCAG-
AGGAAGCTTTAGGCGCGGCACCTTCTGAGTCCCCTGGAACGGGGCGCCATAGAG

GGTGAGAGCCCCGTATAGTTGGATGCCTAGCCTGTGTAAAGCTCC-
TTCGACGAGTCGAGTAGTTTGGGAATGCTGCTCAAAATGGGAGGTAAATTTCTTC
TAAAGCTAAATACCGGCCAGAGACCGATAGCGCACAAGTAGAGTGATCGAAAGA
TGAAAAGCACTTTGAAAAGAGGGTTAAATAGCACGTGAAATTGTTGAAAGGGAA
GCGCTTGTGACCAGACTTGCGCCGGGCTGATCATCCGGTGTTCTCACCGGTGCAC
TCTGCCCGGCTCAGGCCAGCATCGGTTCTCGCGGGGGGATAAAGGTTCCGGGAAT
GTAGCTCCTCCGGGAGTGTTATAGCCCGGGGCGTAATGCCCTCGCGGGGACCGAG
GTTCGCGC-
TCTGCAAGGATGCTGGCGTAATGGTCATCAGCGACCCGTCTTGAAACACGGACCA
AGGAGTCAAGGTTTTGCGCGAGTGTTTGGGTGTAAAACCCGCACGCGTAATGAAA
GTGAACGTAGGTGAGAGCTTCGGCGCATCATCGACCGATCCTGATGTTTTCGGAT
GGATTTGAGTAAGAGCGTTAAGCCTTGGACCCGAAAGATGGTGAACTATGCTTGG
ATAGGGTGAAGCCAGAGGAAACTCTGGTGGAGGCTCGCAGCGG-
TTCTGACGTGCAAATCGATCGTCAAATCTGAGCATCTGGTCACATTGGTGCAAGG
TCTGCGGAGGAAGAACGTCATTTCGTTCGAGGTGTCGTTGGTTCGCGACATCCGC
GACCGCGAGTTCAAGATCTTTTCCGATGCTGGGCGCGTCATGAGGCCGCTGTTTA
CCGTCGAGCAGGAGCATGGTTCAGAGACCGGCGCCGAAATGGGGCAACTCATCC
TCAACAAGGAGCATATTACACGGTTAGAAGCGGACAAGGAGCTGGGCAAATACC
ATCCCGATTATTGGGGCTGGCAGGGCCTGCTGAAGTCAGGTGCCATCGAGTACCT
CGATGCCGAGGAGGAGGAGACGGCCATGATCTGCATGACGCCCGAGGATCTCGA
CAAGTTCCGGTACAGAAAGATGGGGTTCATCGTCGAAGACAACTCGGGTC---
AAGGTAACAACAGGATCAAGACGAAGCCGAACCCCGCCACCCACATGTACACGC
ACTGCGAGATCCATCCCAGCATGCTGCTCGGCATCTGCGCCAGCATCATCCCCTTC
CCCGACCACAACCAG??????????????AGC-TTTCTTTTGGCCCCCCTGAT---CTATCC--
CACACATTGG-GATAAC-CTCG-TTCCACCCGCT------ACCCGA---CCGAC------
CGAGAACGATGACGGCGCGGCCATAAT--GGACAGG-------
ACACGCGTGCTGACAT--
GGCTTCTTTACTACAGGTTCACCTCCAGACCGGCCAGTGCGTAAGTTTGATCGATT
C-GAGC-CCGACGACCGATACTG----TGTGGTGG-TGC--GGGTGAAGACTGACT-
ACAAGCT-
CTCCAGGGTAACCAAATTGGTGCTGCTTTCTGGTGTGTTCGACGCCAA-
CATTAACCGCTTGCGTGTC-AA-AGGCAAT---
CAAGACTGACTTCCATTACAGGCAGACCATCTCTGGTGAGCACGGCCTCGACAGC
AATGGCGTGTACGTGACTGTCGCCGACCCAAATC-CCCATAATCTCCC-
TTGCTCACCGCTACAAATAGGTACAACGGCACCTCGGAGCTCCAGCTCGAGCGCA
TGAATGTCTACTTCAACGAGGTGAGTCAGGCC--AGTACACCA----TCAATTTTC-----
TTGAAGGCTGCGGGTCGCTGACAAC-ATAA----
TCTTCGCAGGCTTCCGGCAACAAATATGTCCCTCGTGCCGTCCTCGTCGACTTGGA
GCCCGGCACCATGGATGCTGTTCGCGCTGGTCCCTTCGGCCAGCTCTTCCGCCCTG
ATAACTT

>Humicola_fuscoatra_CBS_118_14

ATTACAGAGTTGC-AAAACT-CCCAAACCATTGTGAACAT-ACC-TA-
CCCCGTTGCTTCGGCGGGCGG-CCCGGGCCC-CGCGCCC---------------
GGCGCCCCCCGGCCCCCC---------------------
GTGGGCGCCCGCCGGAGGTAAAACAAACCCTTGAAT-TGC--
ATGGCCTCTCTGAGTCTTCTGTACTGAA-

TAAGTCAAAACTTTCAACAACGGATCTCTTGGTTCTGGCATCGATGAAGAACGCA
GCGAAATGCGATAAGTAATGTGAATTGCAGAATTCAGTGAATCATCGAATCTTTG
AACGCACATTGCGCCCGCCAGTATTCTGGCGGGCATGCCTGTTCGAGCGTCATTT
CAACCATCAAGCCCCC-GGCTTGTGTTGGGGACCTGCG----------GCTG-
CCGCAGGCCCTGAAAACCAGTGGCGGGCTCGCT-GTCACCCCGAGCGTAGTAG---
TTACATCTCGCTCTGGGAG-TGCTGCGGGTT--CCGGCCGTTAAA-CGCCTTA-
TTTACCCAAGGTTGACCTCGGATCAGGTAGGAAGACCCGCTGAACTTAAGCATAT
CAATAAGCGGAGGAAAAGAAACCAACAGGGATTGCCC-CAGTAACGGCG-
AGTGAAGCGGCAACAGCTCAAATTTGAAATCTGGCTTCGGCCCGAGTTGTAATTT
GCAG-
AGGAAGCTTTAGGCGCGGCACCAACTGAGTCCCCTGGAACGGGGCGCCACAGAG
GGTGAGAGCCCCGTATAGTTGGACGCCTAGCCTGTGTAAAGCTCC-
TTCGACGAGTCGAGTAGTTTGGGAATGCTGCTCAAAATGGGAGGTAAATTTCTTC
TAAAGCTAAATACCGGCCAGAGACCGATAGCGCACAAGTAGAGTGATCGAAAGA
TGAAAAGCACTTTGAAAAGAGGGTTAAATAGCACGTGAAATTGTTGAAAGGGAA
GCGCTTGTGACCAGACTTGCGCCCGGCTGATCATCCGGTGTTCTCACCGGTGCACT
CTGCCGGGCTCAGGCCAGCATCGGTTCTCGCGGGGGGATAAAGGTCCTGGGAAC
GTAGCTCCTCCGGGAGTGTTATAGCCCAGGGCGTCATGCCCTCGCGGGGACCGAG
GTTCGCGCATCTGCAAGGATGCTGGCGTAATGGTCATCAGCGACCCGTCTTGAAA
CACGGACCAAGGAGTCAAGGTTTTGCGCGAGTGTTTGGGTGTAAAACCCGCACGC
GTAATGAAAGTGAACGTAGGTGAGAGCTTCGGCGCATCATCGACCGATCCTGATG
TTTTCGGATGGATTTGAGTAGGAGCGTTAAGCCTTGGACCCGAAAGATGGTGAAC
TATGCTTGGATAGGGTGAAGCCAGAGGAAACTCTGGTGGAGGCTCGCAGCGG-
TTCTGACGTGCAAATCGATCGTCAAATCTGAGCATCTGGTCACGCTGGTCCAGGG
GCTGCGGAGAAAGAACGTCATTTCGTTCGAGGTGTCGCTCGTCAGAGACATCCGC
GACCGCGAGTTCAAGATTTTCTCGGATGCCGGCCGTGTCATGAGGCCGCTCTACA
CGGTAGAGCAAGAGCCAAACAGCGACAGCGGCGCCGAAATCGGGCAGCTGATCC
TGAACAAGGAGCACGTTACGCGGCTCGAGGCCGACAAAGAGCTGGGCAAGTACC
ATCCCGACTACTGGGGCTGGCAAGGCCTCTTGAAGTCGGGTGCCATCGAGTACCT
CGACGCCGAGGAGGAGGAGACGGTCATGATCTGCATGACCCCCGAAGACCTCGA
CAAGTTCCGGTACCGCAAGCTGGGGTTCATCGTGGAGGACAACTCTGGCC---
AAGGCAACAACAGAATCAAGACGAGGCCGAACCCGACGACGCACATGTACACGC
ATTGCGAGATCCACCCCAGCATGCTGCTCGGCATCTGCGCGAGCATCATCCCGTT
CCCCGACCACAACCAG??GCGTC-CGAGAAGC---TTTTTTTGGACCCCTGAT---
CTACCC----CACATCGG-GGCGAC-GTCG-TCCCACCAGCT------
TCCCGAGGCCCAACT-----CGCAGGCGTTGGCGACGCGGCCACGAC--
AACAAGAGACCCGGGCGCG-
ATGCTGACATTATGTTTTTCTGCTACAGGTTCACCTCCAGACCGGCCAGTGCGTAA
GTTGCACCGACTC-GAACGCTGGCGACCGA-------TATACCGTGG-TGC--GGGTG--
GACTGACG-TTGAAG--
CTCTAGGGTAACCAAATTGGTGCCGCTTTCTGGTATGATCCGACCCAA-
CCTCAAGAGCCACGCTATC-CA-TGGCAAT---
CAATACTGACTTCCGCTCCAGGCAGACCATCTCCGGCGAGCACGGCCTCGATGGC
AGTGGCGTGTACGTGCCTGTCGCCGCGTCCTCTCTCGATTAATCCCTCGTCGCTCA
CCGCTCC-
CATAGGTACAATGGCACCTCCGAGCTCCAGCTCGAGCGCATGAACGTCTACTTCA
ACGAGGTCAGTCGGACT--CATCTCCCTGTCGTGGGAATGT-----CGAGCGACTCT-

GGTTTCTGACAAT-
GCATGTCAATTCTACAGGCTTCCGGCAACAAGTATGTTCCTCGTGCCGTCCTGGTC
GACTTGGAGCCCGGCACCATGGATGCCGTCCGCGCTGGTCCCTTCGGCCAGCTCT
TCCGCCCTGACAACTT

>Humicola_olivacea_DTO_319_C7

ATTACAGAGTTGC-AAAACT-CCCAAACCATTGTGAACGTTACC-TTTA-
CCGTTGCTTCGGCGGGCGG-CCCGGGTCC-TGCCC------------------
GGCGCCCCTCGGCCCTCG----------------------
CGGGCGCCCGCCGGAGGTAAACCAAACTATTGCAT-TGT--
ATGGCCTCTCTGAGTCTTCTGTACTGAA-
TAAGTCAAAACTTTCAACAACGGATCTCTTGGTTCTGGCATCGATGAAGAACGCA
GCGAAATGCGATAAGTAATGTGAATTGCAGAATTCAGTGAATCATCGAATCTTTG
AACGCACATTGCGCCCGCCAGTATTCTGGCGGGCATGCCTGTTCGAGCGTCATTT
CAACCATCAAGCCCCC-GGCTTGTGTTGGGGACCTGCG----------GCTG-
CCGCAGGCCCTGAAAACCAGTGGCGGGCTCGCTAGTCACTCCGAGCGTAGTAG--
TTTACATCTCGCTCAGGGCG-TGCTGCGGGTT--CCGGCCGTTAAA-
AGCCTTATTTTACCCAAGGTTGACCTCGGATCAGGTAGGAAGACCCGCTGAACTT
AAGCATATCAATAAGCGGAGGAAAAGAAACCAACAGGGATTGCCC-
TAGTAACGGCG-
AGTGAAGCGGCAACAGCTCAAATTTGAAATCTGGCTTCGGCCCGAGTTGTAATTT
GCAG-
AGGAAGCTTTAGGCGCGGCACCAACTGAGTCCCCTGGAACGGGGCGCCACAGAG
GGTGAGAGCCCCGTATAGTTGGACGCCTAGCCTGTGTAAAGCTCC-
TTCGACGAGTCGAGTAGTTTGGGAATGCTGCTCAAAATGGGAGGTAAATTTCTTC
TAAAGCTAAATACCGGCCAGAGACCGATAGCGCACAAGTAGAGTGATCGAAAGA
TGAAAAGCACTTTGAAAAGAGGGTTAAATAGCACGTGAAATTGTTGAAAGGGAA
GCGCTTGTGACCAGACTTGCGCCCGGCTGATCATCCGGTGTTCTCACCGGTGCACT
CTGCCGGGCTCAGGCCAGCATCGGTTCTCGCGGGGGGGTAAAGGCCCTGGGAAC
GTAGCTCCTCCGGGAGTGTTATAGCCCAGGGCGTAATGCCCTCGCGGGGACCGAG
GTTCGCGCATCTGCAAGGATG??
??
??
???CTGGTGACGCTGG
TCCAAGGGTTGCGGCGAAAGAACGTCATCTCCTTCGAGGTCTCGCTTGTCCGCGA
CATCCGCGATCGCGAGTTCAAGATCTTCTCCGACGCCGGCCGTGTGATGAGGCCA
CTGTACACGGTCGAGCAAGAGCAGAACAGCGACAGCGGTGCGGAAGTCGGACAG
CTGATTCTGAACAAGGAGCATGTTACGCGCCTGGAGGCGGACAAGGAGCTGGGC
AAGTACCATCCCGACTACTGGGGCTGGCAGGGCCTGCTCAAGTCGGGTGCCATCG
AGTACCTCGACGCCGAGGAGGAGGAAACCGTCATGATTTGCATGACGCCCGAGG
ACCTCGATAAATTTCGGTACCGCAAGATGGGCTTTATTGTCGAGGACAACTCTGG
TC---
AAGGCAACAACAGGATCAAGACGAGGCCGAATCCGACAACTCACATGTACACGC
ATTGCGAGATCCACCCCAGCATGCTGCTCGGTATCTGCGCGAGCATTATTCCCTTC
CCCGACCACAACCAG??GCGTC-CGAGAAGC--TTTTTCTAGGACCCCTGAT---
CTACCC--CACATCGGGA-AGACAG-GTCG-TTCCACCACCAGCT-TGTCCCGA---
CCAGCTCC--GGGCGGGCGATGGCGATGCCGCCGTGAATCACAAGCG-------

ACGCG-ATACTGACAT--
TGTTCTCTCACTACAGGTTCACCTCCAGACCGGCCAGTGCGTAAGTTGAGCCGAC
GC-GAA-GACGACTCTCGATCGA-----TCAGGTGGTTGC--AGGTG--GACTGACG-GT-
GGCC-
TTCCAGGGTAACCAAATCGGTGCCGCTTTCTGGTATGTTCAGCCTCGAGCATCAA
GACGTGTGTTTTGTATCGACGGCATCAGAATACTGACTTTCTATCCAGGCAGACC
ATCTCCGGCGAGCACGGCCTCGACGGCAATGGCGTGTACGTGACCGTCGCCGCCC
CGCGTCTCGATTAATCAC---TCGCTCACCGCCAC-
CACAGGTACAATGGCAGCTCCGAGCTCCAGCTCGAGCGCATGAACGTCTACTTCA
ACGAGGTCAGTCGTGCTC-GCCAGAACGT---TAATGTGGT------------CCGG-
GGTTGCTGACAAG-TGATGT-
TTTGCTGCAGGCCTCGGGCAACAAGTATGTCCCCCGTGCCGTCCTCGTCGACTTGG
AGCCCGGCACCATGGATGCCGTCCGCGCCGGTCCCTTCGGCCAGCTCTTCCGCCC
CGACAACTT

>Parachaetomium_hispanicum_CBS_234_82

ATTACAGAGTTGC-AAAACTCCCTAAACCATTGTGAACGTTACCC--
AAACCGTTGCTTCGGCGGGCGG-CGCCCA--------------------------
GCGCGCCCCCCCGGCCCCTC--------------------
GCGGGGCGCCCGCCGGAGGTACCCCAAACCATTGATACTTT--
ATGGCCTCTCTGAGTCTTCTGTACTGAA-
TAAGTCAAAACTTTCAACAACGGATCTCTTGGTTCTGGCATCGATGAAGAACGCA
GCGAAATGCGATAAGTAATGTGAATTGCAGAATTCAGTGAATCATCGAATCTTTG
AACGCACATTGCGCCCGCCAGTATTCTGGCGGGCATGCCTGTTCGAGCGTCATTT
CAACCATCAAGCCCCCCGGCTTGTGTTGGGGACCTGCG----------GCTG-
CCGCAGGCCCTGAAAAGCAGTGGCGGGCTCGCT-GTCACACCGAGCGTAGTAG--
ATTTCATCTCGCTCTGGGCG-TGCTGCGGGTT--
CCGGCCGTTAAACCACCTTTCATAACCCAAGGTTGACCTCGGATCAGGTAGGAAG
ACCCGCTGAACTTAAGCATATCAATAAGCGGAGGAAAAGAAACCAACAGGGATT
GCCC-TAGTAACGGCG-
AGTGAAGCGGCAACAGCTCAAATTTGAAATCTGGCCTCGGCCCGAGTTGTAATTT
GCAG-
AGGAAGCTTTAGGCGCGGCACCAACTGAGTCCCCTGGAACGGGGCGCCATAGAG
GGTGAGAGCCCCGTATAGTTGGACGCCTAGCCTGTGTAAAGCTCC-
TTCGACGAGTCGAGTAGTTTGGGAATGCTGCTCAAAATGGGAGGTAAATTTCTTC
TAAAGCTAAATACCGGCCAGAGACCGATAGCGCACAAGTAGAGTGATCGAAAGA
TGAAAAGCACTTTGAAAAGAGGGTTAAATAGCACGTGAAATTGTTGAAAGGGAA
GCGCTTGTGACCAGACTTGCGCCGGGCGGATCATCCGGTGTTCTCACCGGTGCAC
TCCGCCCGGCTCAGGCCAGCATCGGTTCTCGCGGGGGGATAAAGGCCCTGGGAAC
GTAGCTCCTCCGGGAGTGTTATAGCCCAGGGCGCAATGCCCTCGCGGGGACCGAG
GTTCGCGCATCTGCAAGGATG??
??
??
??CTAGTCACCCTGG
TCCAGGGGCTGCGGAGAAAGAACGTCATCTCGTTCGAGGTCTCACTCGTCCGCGA
TATCCGCGACCGCGAGTTCAAGATCTTCTCGGATGCGGGCCGTGTCATGAGGCCT
CTATACACGGTGGAGCAAGATCCAAACGGCGAGAGCGGCGCCGAGATGGGCGCG

CTGATCCTCAACAAGGAGCACGTCGCGCGGCTGGATACCGACAAGGACCTTGGG
AGGTACCATCCGGACTACTGGGGCTGGCAAGGGCTGCTGAAGTCGGGTGCCATCG
AGTACCTCGATGCCGAGGAGGAGGAGACGGTCATGATCTGCATGACGCCCGAGG
ATCTCGACAACTTCCGTGCGCGCAAGAATGGGCTAAACCCCGAGGACACGTCGG
GCC---
TAGGCAACAATCGGATCAAGACGAAGCCGAACCCGACGACTCACATGTACACCC
ATTGCGAGATCCATCCCAGCATGCTGCTCGGCATCTGCGCGAGCATCATCCCCTTC
CCCGACCACAACCAGACGCGTC-TAGGAAGC---TTTTTTGGTGCCCCTGAT---
CTACCC--CACACGGCGA-GACGAC-CTTG-TTCCACCGGCT-------CTCGA---CCGAC--
----CGACGGCGATGGCGGCGCTGCCATGAC--GGGAGGA-------ACACG-
ATGCTGACTG--
CTTCTCCGTGCTACAGGTTCACCTTCAGACCGGCCAGTGCGTAAGTTGGACCGATT
C-GAAC-CCCGCGACCGA---------TC-GGTGG-TGC--GGGTT--GACTGACA-
TCAAGCT-
CTCCAGGGTAACCAAATCGGTGCCGCTTTCTGGTACGTCCAAGCTCGA-
AACCGAGCACTCCCATCGC-GG-ATGACAT---
CGAGACTGACTTCTTCTCCAGGCAGACCATCTCTGGCGAGCACGGCCTCGACAGC
AATGGCGTGTACGTAGCCATCGACGGTGCCGAT--CGATTAACCCC---
TCGCTCACCGCTTC-
CATAGGTACAACGGCACCTCCGAGCTCCAGCTCGAGCGCATGAACGTCTACTTTA
ACGAGGTGAGTCGTGT---TATACGCCT----TCAAACCGT-----CGAGCGACGGC---
GTGCTGACAAT-ACAT----
CTTCGACAGGCTTCCGGCAACAAGTATGTCCCCCGTGCCGTCCTGGTCGACTTGG
AGCCCGGCACCATGGACGCCGTCCGCGCCGGCCCCTTCGGCCAGCTCTTCCGCCA
GGACAACTT

>Parachaetomium_perlucidum_CBS_141_58

ATTACAGAGTTGC-AAAACTCCCTAAACCATTGTGAACGTTACCC--
AAACCGTTGCTTCGGCGGGCGGGCGCCCA-------------------------
GCGCGCCCCCCGGGCCCCTC-------------------
GCGGGGCGCCCGCCGGAGGTACCCCAAACCATTTGATATTT--
ATGGCCTCTCTGAGTCTTCTGTACTGAA-
TAAGTCAAAACTTTCAACAACGGATCTCTTGGTTCTGGCATCGATGAAGAACGCA
GCGAAATGCGATAAGTAATGTGAATTGCAGAATTCAGTGAATCATCGAATCTTTG
AACGCACATTGCGCCCGCCAGTATTCTGGCGGGCATGCCTGTTCGAGCGTCATTT
CAACCATCAAGCCCCCGGGCTTGTGTTGGGGACCTGCG----------GCTG-
CCGCAGGCCCTGAAAAGCAGTGGCGGGCTCGCT-GTCACACCGAGCGTAGTAG--
ATTTTATCTCGCTCTGGGCG-TGCTGCGGGTT--CCGGCCGTTAAACCACCCTT-
TTAACCCAAGGTTGACCTCGGATCAGGTAG???
???????????????ATTGCCC-TAGTAACGGCG-
AGTGAAGCGGCAACAGCTCAAATTTGAAATCTGGCCTCGGCCCGAGTTGTAATTT
GCAG-
AGGAAGCTTTAGGCGCGGCACCAACTGAGTCCCCTGGAACGGGGCGCCATAGAG
GGTGAGAGCCCCGTATAGTTGGACGCCTAGCCTGTGTAAAGCTCC-
TTCGACGAGTCGAGTAGTTTGGGAATGCTGCTCAAAATGGGAGGTAAATTTCTTC
TAAAGCTAAATACCGGCCAGAGACCGATAGCGCACAAGTAGAGTGATCGAAAGA
TGAAAAGCACTTTGAAAAGAGGGTTAAATAGCACGTGAAATTGTTGAAAGGGAA

GCGCTTGTGACCAGACTTGCGCCGGGCGGATCATCCGGTGTTCTCACCGGTGCAC
TCCGCCCGGCTCAGGCCAGCATCGGTTCTCGCGGGGGATAAAGGCCTCGGGAAC
GTAGCTCCTCCGGGAGTGTTATAGCCCGGGGCGCAATGCCCTCGCGGGGACCGAG
GTTCGCGCATCTGCAAGGATGCTGGCGTAATGGTCATCAGCGACCCGTCTTGAAA
CACGGACCAAGGAGTCAAGGTTTTGCGCGAGTGTTTGGGTGTCAAACCCGCACGC
GTAATGAAAGTGAACGTAGGTGAGAGCTTCGGCGCATCATCGACCGATCCTGATG
TTTTCGGATGGATTTGAGTAGGAGCGTTAAGCCTTGGACCCGAAAGATGGTGAAC
TATGCTTGGATAGGGTGAAGCCAGAGGAAACTCTGGTGGAGGCTCGCAGCGG-
TTCTGACGTGCAAATCGATCGTCAAATCTGAGCATCTGGTCAACCTGGTCCAGGG
GCTGCGGAGGAAGAACGTCATCTCGTTCGAGGTTTCGCTCGTCCGCGACATCCGC
GACCGCGAGTTCAAGATTTTTTCGGATGCCGGCCGCGTCATGAGGCCTTTGTACA
CGGTGGAGCAAGATCCAAATGGCGAGAGCGGCGCCGAGATGGGCGCGCTGATCC
TGAACAAGGAGCACGTCGCGCGGCTGGATACGGACAAGGACCTTGGGAGGTATC
ATCCGGATTACTGGGGCTGGCAGGGGCTGCTGAAGTCGGGTGCCATCGAGTACCT
CGATGCTGAGGAAGAGGAGACGGTCATGATTTGCATGACGCCCGAGGATCTCGA
CAATTTCCGCGCGCGCAAGAATGGGCTAAACCCCGAGGACACGTCGGGCC---
TGGGCAACAATCGGATCAAGACGAAGCCGAACCCGACGACGCACATGTACACAC
ATTGCGAGATCCACCCCAGCATGCTGCTGGGTATCTGCGCGAGCATCATCCCGTT
CCCCGACCACAACCAGACGCGTC-TGGGCAGC---CTTTTTGTGGCCCCTGAT---
CTACCC--CACACGTCGA-GACGAC-CTCG-TTCCACCAGCT-------CCCGA---CCGAC--
----CGACGGCGATGGCGGCGCGGCCATGAT--AGGAGGG-------ACACG-
ATGCTGACTG--
TTTCTCCCCGCTACAGGTTCACCTCCAGACCGGCCAGTGCGTAAGTTGGACCGATT
T-CAAC-ACCGCGACCCA---------TC-GGTGG-TGC--GGCTT--GACTGACA-TCAAGCT-
CTCCAGGGTAACCAAATCGGTGCCGCTTTCTGGTATGTCCAAGCTCCA-
AACCGAGAACTCCCATCGC-GG-ATGACAT---
CGAGACTGACTTCTTCTCCAGGCAGACCATCTCTGGCGAGCACGGCCTCGACAGC
AATGGCGTGTACGTAGCCATCGACGGCCCCGAT--CGATTAACCCC---
TCGCTCACCGCTTC-
GATAGGTACAACGGCACCTCCGAGCTCCAGCTTGAGCGCATGAACGTCTACTTCA
ACGAGGTGAGTCGTGTTGTTGTATACCT----TCAAGCAAT-----CGTGCGATGGC--
GTTGCTGACAAT-ACAT----
CTTGGACAGGCTTCCGGCAACAAGTATGTTCCCCGTGCCGTCCTGGTCGACTTGG
AGCCCGGCACCATGGATGCCGTCCGCGCCGGCCCCTTCGGCCAGCTCTTCCGCCC
GGACAACTT

>Parathielavia_kuwaitensis_CBS_945_72

???C-
TCAACCGTTGCTTCGGCGGGCGGGCCCGGGCCTCACCGCCC--------------
GGACGCCCCCCCAGGCCCC-TC-----------------GCGGGGCGCCCGCCGGAGGTACCC-
AAACTCTTGAATATAT--ATGGCCTCTCTGAGTCTTCTGTACTGAA-
TCAGTCAAAACTTTCAACAACGGATCTCTTGGTTCTGGCATCGATGAAGAACGCA
GCGAAATGCGATAAGTAATGTGAATTGCAGAATTCAGTGAATCATCGAATCTTTG
AACGCACATTGCGCCCGCCAGTATTCTGGCGGGCATGCCTGTCCGAGCGTCATTT
CAACCATCAAGCCCC-GCGCTTGTGTTGGGGTCCTGCG----------
GCTGCCCGCAGGCCCTGAAAACCAGTGGCGGGCTCGCT-
GTCGCACCGAGCGTAGTAG--CATAA-

CCTCGCTCAG??
??????????????????GCATATCAATAAGCGGAGGAAAAGAAACCAACAGGGATTGCC
C-CAGTAACGGCG-
AGTGAAGCGGCAACAGCTCAAATTTGAAATCTGGCCTCGGCCCGAGTTGTAATTT
GCAG-
AGGAAGCTTTAGGCGCGGCCCCAACTGAGTCCCCTGGAACGGGGCGCCACAGAG
GGTGAGAGCCCCGTATAGTTGGACGCCTAGCCTGTGTAAAGCTCC-
TTCGACGAGTCGAGTAGTTTGGGAATGCTGCTCAAAATGGGAGGTAAATTTCTTC
TAAAGCTAAATACCGGCCAGAGACCGATAGCGCACAAGTAGAGTGATCGAAAGA
TGAAAAGCACTTTGAAAAGAGGGTTAAATAGCACGTGAAATTGTTGAAAGGGAA
GCGCTTGTGACCAGACTTGCGCCGGGCTGATCATCCGGTGTTCTCACCGGTGCAC
TCTGCCCGGCTCAGGCCAGCATCGGTTCTCGCGGGGGGACAAAGGCTCCGGGAAC
GTAGCTCCTCCGGGAGTGTTATAGCCCGGGGCGCAATGCCCCCGCGGGGACCGAG
GACCGCGC-
TCTGCAAGGATGCTGGCGTAATGGTCATCAGCGACCCGTCTTGAAACACGGACCA
AGGAGTCAAGGTTTTGCGCGAGTGTTTGGGTGTAAAACCCGCACGCGTAATGAAA
GTGAACGTAGGTGAGAGCTTCGGCGCATCATCGACCGATCCTGATGTTCTCGGAT
GGATTTGAGTAGGAGCGTTAAGCCTTGGACCCGAAAGATGGTGAACTATGCTTGG
ATAGGGTGAAGCCAGAGGAAACTCTGGTGGAGGCTCGCAGCGG-
TTCTGACGTGCAAATCGATCGTCAAATCTGAGCATCTGGTCAGCTTGGTTCAGGG
ATTGCGGCGAAAGAACGTCATCTCGTTCGAGGTTTCCCTTGTCAGGGACATCCGC
GACCGCGAGTTCAAGATCTTCTCGGATGCCGGCCGTGTCATGAGGCCGCTCTACA
CCGTCGAGCAAGAGCCAAACAGCGAGACCGGGGCCGAGGTGGGGCAGCTGATTC
TCAACAAGGAACACATCAGCCGGTTGGAAGCGGACAAGGAGCTGGGCAAGTACC
ATCCCGATTACTGGGGTTGGCAGGGGCTGCTCAAGTCGGGTGCCATCGAGTACCT
GGATGCCGAGGAGGAAGAGACAGTCATGATCTGCATGACGCCCGAAGACCTCGA
CAAGTTCCGTTACCGCAAGATGGGCTTCATCATCGAAGACAATTCTGGTC---
AGGGTAACAACAGGATCAAGACGAAGCCGAACCCGACGACCCACATGTATACCC
ACTGCGAGATCCATCCCAGCATGCTGCTCGGCATCTGCGCGAGCATCATCCCCTT
CCCCGACCACAACCAG??GCGTC-TGGGTAGC--ATTTTTTTGGGCCCCTGAT----
TACCC--CACACATTGA-GACGAC-GTCG-TCCCACCCGTT-------CCCGA---CCGAC----
--CGCTGGCGATGGCGGCGCTGCCGTGAT--GGAAAGC-------ACGCG-
ATGCTGACTT--
TGTTTCTTTGCTACAGGTTCACCTCCAGACCGGCCAGTGCGTAAGTAGAATCGACT
C-GGAC-CCGACCGACAACCCA-----TCAGATGG-TGCTTGGGTG--GACTGACA-
GCGAGCT--
CCCAGGGTAACCAAATCGGTGCCGCCTTCTGGTAGGTCAACCTCGAAAACAGCCA
ATCCTTCATAACTCA-AGGTGAT---
TGATGCTGACGTCCTCGATAGGCAGACCATCTCTGGCGAGCACGGCCTCGACAGC
AATGGCGTGTACGTGACTACCACC-ATCCCGATC-GATGTACTCCC---
TCGCTCACCGCTAC-
AATAGGTACAACGGAACCTCCGAGCTCCAGCTCGAGCGCATGAACGTCTACTTCA
ACGAGGTTAGTCGACGT--TATATACCTG-
CGCGATAATACAGCGAGGAGCAGCCGT-GTTTGCTGACGA----GC----
GATCCTCAGGCTTCCGGCAACAAGTATGTCCCTCGTGCCGTCCTCGTCGACTTGGA
GCCCGGCACCATGGACGCCGTCCGCGCTGGCCCCTTCGGCCAGCTCTTCCGCCCT
GACAACTT

>Subramaniula_anamorphosa_CBS_137114

ATTACAGAGTTAC-CCAACT-CCCAAACCATTGTGAACGTTACC--
TCTCCCGTTGCTTCGGCGGGCGGG-CCGGCCCC-AGGGC------------------
CGCGCCCCCCCGGCCCCC-TC------------------GCGGGGCGCCCGCCGGAGGAAACCC-
AACTCTTGATTATC---ATGGCCTCTCTGAGTCTTCTGTACTGAA-
TAAGTCAAAACTTTCAACAACGGATCTCTTGGTTCTGGCATCGATGAAGAACGCA
GCGAAATGCGATAAGTAATGTGAATTGCAGAATTCAGTGAATCATCGAATCTTTG
AACGCACATTGCGCCCGCCAGTATTCTGGCGGGCATGCCTGTTCGAGCGTCATTT
CAACCATCAAGCCCC-GTGCTTGTGTTGGGGACCTGCG----------GCTG-
CCGCAGGCCCTGAAAACCAGTGGCGGGCTCGCT-GTCACACCGGGCGTAGTAA---
TTACATCTCGCTCAGGGCG-TGCTGCGGGTT--CCGGCCGTAAAA-
AGCCTTATTTTACCCAAGGTTGACC??
??GTGAAGCGGCAACAGCTCAAATTTGAA
ATCTGGCTTCGGCCCGAGTTGTAATTTGCAG-
AGGAAGCTTTAGGCGCGGCACCATCTGAGTCCCCTGGAACGGGGCGCCACAGAG
GGTGAGAGCCCCGTATAGATGGACGCCTAGCCTGTGTAAAGCTCC-
TTCGACGAGTCGAGTAGTTTGGGAATGCTGCTCAAAATGGGAGGTAAATTTCTTC
TAAAGCTAAATATTGGCCAGAGACCGATAGCGCACAAGTAGAGTGATCGAAAGA
TGAAAAGCACTTTGAAAAGAGGGTTAAATAGCACGTGAAATTGTTGAAAGGGAA
GCGCTTGTGACCAGACTTGCGCCGGGCGGATCATCCGGTGTTCTCACCGGTGCAC
TCCGCCCGGCTCAGGCCAGCATCGGTTCTCGCGGGGGGATAAAGGCCCTGGGAAC
GTAGCTCCTCCGGGAGTGTTATAGCCCAGGGTGTAATGCCCTCGCGGGGACCGAG
GTTCGCGCATCTGCAAGGATGCTGGCGTAATGGTCATCAGCGACCCGTCTTGAAA
CACGGACCAAGGAGTCAAGGTTTTGCGCGAGTGTTTGGGTGTAAAACCCGCACGC
GTAATGAAAGTGAACGTAGGTGAGAGCTTCGGCGCATCATCGACCGATCCTGATG
TTTTCGGATGGATTTGAGTAGGAGCGTTAAGCCTTGGACCCGAAAGATGGTGAAC
TATGCTTGGATAGGGTGAAGCCAGAGGAAACTCTGGTGGAGGCTCGCAGCGG-
TTCTGACGTGCAAATCGATCGTCAAATCTGAGCATCTGGTCACGTTGGTGCAAGG
GTTGCGCCGGAAGAACGTCATCTCGTTTGAGGTTTCGCTTGTCCGCGACATTCGCG
ACCGCGAGTTCAAGATCTTCTCCGATGCGGGCCGCGTCATGAGGCCGCTGTTTAC
GGTGGAGCAGGAGCAGAACGCGGAGAGCGGGGTTGAGGTCGGGCAGCTGATTCT
CAACAAAGAGCACATCACGCGGCTAGAGGCGGACAAGGAGATAGGCCGATACCA
CCCCGACTACTGGGGTTGGCAGGGCTTGCTCAAGTCTGGTGCCATCGAGTATCTC
GATGCCGAAGAGGAAGAGACCGTCATGATTTCCATGACGCCCGAGGATCTCGAC
AAGTTCCGGTACCGCAAGATGGGATTCGTCGTTGAGGACAACTCCGGCC---
AGGGTAACAACAGGATCAAGACAAGACCCAATCCGGCGACGCACATGTACAC???
??A
GC--TTTTTCCTGGGCCCCTGAT---CTACCC--CACACACTGG-GACAAC-
CTCATTCCCACGAGCT-------ACCGA---CCGGT-------
CCGAGCGCGACAGGTGTAGGCATGAT--GAAAAGA-------GCACC-ATGCTAACAA--
TGTTCCCTATCTACAGGTCCACCTCCAGACCGGCCAGTGCGTAAGTTGAACCGAT
TT-GATACCGGGCGACCGA---------TCAGGCGG-TGC--GGGTG--AACTGACA-
GCGTGCC-TCTCAGGGTAACCAAATTGGTGCTGCTTTCTGGTATGTTCAGCCTCA--
CGACAGACGTCGACGTGCAAAA-GGGCGAC---TGATACTGACT-
CCTCTCCAGGCAGACCATCTCTGGCGAGCACGGCCTCGACAGCAATGGCGTGTAC
GTGACCGTCGCCGATACCCGCC-CGACTGACCTC---TTACTCACCGCTTC-
GACAGGTACAACGGCACCTCGGAGCTGCAGCTCGAGCGCATGAACGTCTACTTCA

ACGAGGTGCGTTGGCTC--TCTCCGCGTC---ATCGGCGAC-----TAAGTGTTCCT-
GGGTGCTGACAAT-CGCT----
ATCATACAGGCGTCCGGCAACAAGTATGTTCCCCGCGCCGTCCTCGTCGACTTGG
AGCCCGGCACCATGGACGCCGTCCGCGCTGGTCCCTTCGGTCAGCTCTTCCGCCC
CGACAACTT

>Subramaniula_cristata_CBS_156_52

ATTACAGAGTTTCTATAACT-CCCAAACCATTGTGAACCTTACC-
TCAAACCGTTGCTTCGGCGGGCGGG-CCGGCCCC-AGGGC------------------
CGCGCCCCCCAGGCCCCTC--------------------GCGGGGCGCCCGCCGGAGGAAACCC-
AACTCTTGATTATT---ATGGCCTCTCTGAGTCTTCTGTACTGAA-
TAAGTCAAAACTTTCAACAACGGATCTCTTGGTTCTGGCATCGATGAAGAACGCA
GCGAAATGCGATAAGTAATGTGAATTGCAGAATTCAGTGAATCATCGAATCTTTG
AACGCACATTGCGCCCGCCAGTATTCTGGCGGGCATGCCTGTTCGAGCGTCATTT
CAACCATCAAGCCCCC-GGCTTGTGTTGGGGACCTGCG----------GCTG-
CCGCAGGCCCTGAAAACCAGTGGCGGGCTCGCT-GTCACACCGAGCGTAGTAG-
ATTTTCATCTCGCTCAGGGCG-TGCTGCGGGTT--CCGGCCGTAAAA-AGCCTTA-
TTTACCCAAGGTTGACCTCGGATCAGGTAGGAAGACCCGCTGAACTTAAGCATAT
CAATAAGCGGAGGAAAAGAAACCAACAGGGATTGCCC-CAGTAACGGCG-
AGTGAAGCGGCAACAGCTCAAATTTGAAATCTGGCTTCGGCCCGAGTTGTAATTT
GCAG-
AGGAAGCTTTAGGCGCGGCACCATCTGAGTCCCCTGGAACGGGGCGCCATAGAG
GGTGAGAGCCCCGTATAGATGGACGCTAAGCCTGTGTAAAGCTCC-
TTCGACGAGTCGAGTAGTTTGGGAATGCTGCTCAAAATGGGAGGTAAATTTCTTC
TAAAGCTAAATATTGGCCAGAGACCGATAGCGCACAAGTAGAGTGATCGAAAGA
TGAAAAGCACTTTGAAAAGAGGGTTAAATAGCACGTGAAATTGTTGAAAGGGAA
GCGCTTGTGACCAGACTTGCGCCTGGCTGATCATCCGGTGTTCTCACCGGTGCACT
CTGCCAGGCTCAGGCCAGCATCGGTTCTCGCGGGGGGATAAAGGCCCTGGGAAC
GTAGCTCCTCCGGGAGTGTTATAGCCCAGGGTGCAATGCCCTCGCGGGGACCGAG
GTTCGCGCATCTGCAAGGATG???
???
???
??CTAGTCACATTAG
TCCAAGGGCTGCGGAGAAAGAATGTCATATCCTTCGAGGTGTCGCTCGTTCGCGA
CATCCGCGACCGCGAGTTCAAGATTTTCTCCGATGCTGGCCGCGTCATGAGGCCG
CTGTTCACTGTAGAGCAAGAGCCCAACGCTGAGAGCGGTGCCGAGGTCGGCCAG
TTGATTCTCAACAAGGACCACATCGCGCGGTTGGAGGCGGATAAAGATCTAGGG
AAGTATCACCCTGACTACTGGGGCTGGCAGGGCCTGCTCAAGTCGGGTGCGATTG
AGTACCTCGACGCTGAGGAGGAGGAAACGGTCATGATCTCCATGACGCCTGAGG
ATCTCGACAAGTTCCGGTACCGCAAGATGGGCTTCGTCGTCGAGGACAACTCTGG
CC---
AGGGCAACAACAGGATCAAGACCAAGCCGAATCCGACCACGCATATGTACACGC
ATTGCGAGATCCACCCCAGCATGCTGCTGGGCATCTGCGCCAGTATCATTCCGTTC
CCCGACCACAACCAGACGCGTC--AGGAAGT---TTTTTCTGGGCCCCTGAT---
TTACCC--CACACGCGAACGTCGAC-CTCATTTCCACGAGCT------ACCCGA---
CCGGT-------CCAAGCGCGATAGACGTCGCCATGAT--GAAGAGA-------GTTTG-
ATGCTGACAT--

CGCTTCTTAACTACAGGTCCACCTCCAGACCGGCCAGTGCGTAAGTTGAATCGAC
TC-GATACCGGACAACCGA---------TCAGGTGG-TGC--AGGTG--GACTGACA-
GCTCACG-CCTTAGGGTAACCAAATCGGTGCTGCTTTCTGGTATGTTCGACTCCAA-
CACCAACCACCGACAGGCA-CA-AGGTGGC---
TGAGACTGACTTCTCCTCCAGGCAGACCATCTCCGGCGAGCACGGCCTTGACAGC
AATGGCGTGTATGTGACCGTTGCCCATCCCCGGC-CGACCGATCTC---
CCGCTCACCGCTAC-
AACAGGTACAACGGCACGTCTGAGCTCCAGCTCGAGCGCATGAACGTCTACTTCA
ACGAGGTAGGTTGGCTT--CACGCCTTCT---TTGGGCACT-----CGGGTG-GCGT-
GGCTGCTGACGAC-AACT----
GTTCTACAGGCTTCCGGCAACAAGTATGTTCCTCGCGCAGTCCTCGTCGACTTGGA
GCCCGGCACCATGGACGCCGTCCGCGCCGGCCCCTTCGGCCAGCTCTTCCGCCCC
GACAACTT

>Parathielavia_hyrcaniae_CBS_353_62

???-
TTCAACCGTTGCTTCGGCGGGCGGGCCCGGGTCTCACCGCCC--------------
GGCCGCCCCACCGGCCCCGCTC-----------------
GCGGGGCGCCCGCCGGAGGTACCCAAAACTCTTGATACTTT--
ATGGCCTCTCTGAGTCTTCTGTACTGAA-
TCAGTCAAAACTTTCAACAACGGATCTCTTGGTTCTGGCATCGATGAAGAACGCA
GCGAAATGCGATAAGTAATGTGAATTGCAGAATTCAGTGAATCATCGAATCTTTG
AACGCACATTGCGCCCGCCAGTATTCTGGCGGGCATGCCTGTTCGAGCGTCATTT
CAACCATCAAGCCCC-GTGCTTGTGTTGGGGACCTGCG----------
GCTGCCCGCAGGCCCTGAAAACCAGTGGCGGGCTCGCT-
GTCACACCGAGCGTAGTAG--
CAACAACCTCGCTCAG???
???GTTCACGG
CGTAGTGAAGCGGCAACAGCTCAAATTTGAAATCTGGCTTCGGCCCGAGTTGTAA
TTTGCAG-
AGGAAGCTTTAGGTGCGGCCCCAACTGAGTCCCCTGGAACGGGGCGCCACAGAG
GGTGAGAGCCCCGTATAGTTGGACGCCTAGCCTGTGTAAAGCTCC-
TTCGACGAGTCGAGTAGTTTGGGAATGCTGCTCAAAATGGGAGGTAAATTTCTTC
TAAAGCTAAATACCGGCCAGAGACCGATAGCGCACAAGTAGAGTGATCGAAAGA
TGAAAAGCACTTTGAAAAGAGGGTTAAATAGCACGTGAAATTGTTGAAAGGGAA
GCGCTTGTGACCAGACTTGCGCCGGGCTGATCATCCGGTGTTCTCACCGGTGCAC
TCTGCCCGGCTCAGGCCAGCATCGGTTCTCGCGGGGGGATAAAGGCTCCGGGAAC
GTAGCTCCTCCGGGAGTGTTATAGCCCGGGGCGTAATGCCCTCGCGGGGACCGAG
GACCGCGC-
TCT???
???
???
??CTGGTCAGTTTGGTTCAAGGATTGCG
GCGAAAGAACGTCATCTCGTTTGAGGTTTCGCTTGTCAGGGACATCCGCGACCGC
GAGTTCAAGATCTTCTCGGATGCTGGCCGTGTCATGAGGCCGCTGTTCACTGTCG
AGCAAGAGCCAAACAGCGAGACCGGGGCTGAGGTGGGCCAGCTGATTCTCAACA
AGGAACACATTAGCCGGTTGGAAGCCGACAAGGAGCTGGGCAAATATCATCCCG

ATTACTGGGGTTGGCAGGGCTTGTTGAAGTCGGGTGCCATCGAGTACCTGGATGC
CGAGGAGGAGGAGACAGTCATGATTTGCATGACACCCGAGGACCTCGACAAGTT
CCGTTACCGCAAGATGGGCTTCATCATCGAAGACAATTCTGGTC---
AGGGTAACAACAGGATCAAGACGAAGCCGAACCCGACTACTCACATGTACACCC
ACTGCGAGATCCATCCCAGCATGCTGCTCGGCGTTTGCGCGAGCATCATCCCGTT
CCCAGACCACAACCAG??GCGTC-TGGCTAGC---
TTTTTTTGGGCCCCTAATTACCTACCC--CACACATCGA-GACGAC-GTCG-
TCCGACCCGCT-------CCCGA---CCGAC------
CGCTGGCGATGGCAGCACTGCCGTGAT--GGAAGGC-------ACACA-ATGCTGACTA--
TGTTTCTTTGCTACAGGTTCACCTCCAGACCGGCCAGTGCGTAAGTAGAATCGAA
TC-GGAC-TCGGCATCCCA---------TCGGATGG-TACTTGGGTG--GACTAACA-
AGAAGCT--
CCCAGGGTAACCAAATCGGGGCCGCCTTCTGGTAAGTTGACCTCGAAAACAACGA
ATCCTTCATAAGTCA-AGGCGAT---
CAATGCTGACATCTTTGATAGGCAGACAATCTCTGGCGAGCACGGTCTCGACAGC
AATGGCGTGTACGTGACTGTCGCCGATCCTGATC-GATTTACCTCC---
TTGCTCACCGCTCC-
GACAGGTACAATGGAACCTCCGAGCTTCAGCTCGAGCGCATGAACGTCTACTTCA
ACGAGGTACGTCGACCT--CATACCACTT-
TACTGTAATACAACGAGGAGCACCCTT-GTTTGCTGACGA----GC----
GAACTTCAGGCTTCTGGCAACAAGTATGTCCCTCGTGCCGTCCTGGTCGACCTGG
AGCCCGGCACCATGGATGCCGTCCGCGCCGGTCCCTTCGGTCAGCTCTTCCGCCCT
GACAACTT

>Hyalosphaerella_fragilis_CBS_456_73

ATTACAGAGTTGC-
AAAACTCCCTAAACCATTGTGAACCTTACCTTTCAACCGTTGCTTCGGCGGGCGG
GCCC-----------------------------CGTGCCCCTGGGCCCCTC----------------------
GCGGGCGCCCGCCGGAGGTCACCCAAACTCTTGATACTTT--
ATGGCCTCTCTGAGTCTTCTGTACCGAA-
TAAGTCAAAACTTTCAACAACGGATCTCTTGGTTCTGGCATCGATGAAGAACGCA
GCGAAATGCGATAAGTAATGTGAATTGCAGAATTCAGTGAATCATCGAATCTTTG
AACGCACATTGCGCCCGCCAGTATTCTGGCGGGCATGCCTGTTCGAGCGTCATTT
CAACCATCAAGCCCC-GGGCTTGTGTTGGGGACCTGCG----------
GCTGCCCGCAGGCCCTGAAAACCAGTGGCGGGCTCGCT-
GTCACACCGAGCGTAGTAG--CATCA-CCTCGCTCAGGGCG-TGCTCCGGGTT--
CCGGCCGTTAAA-
AGCCTTTCCAAACCCAAGGTTGACCTCGGATCAGGTAGGAAGACCCGCTGAACTT
AAGCATATCAATAAGCGGAGGAAAAGAAACCAACAGGGATTGCCT-
TAGTAACGGCGAAGTGAAGCGGCAACAGCTCAAATTTGAAATCTGGCTTCGGCCC
GAGTTGTAATTTGCAG-
AGGAAGCTTTAGGCGCGGCACCTTCTGAGTCCCCTGGAACGGGGCGCCATAGAG
GGTGAGAGCCCCGTATAGTTGGATGCCTAGCCTGTGTAAAGCTCC-
TTCGACGAGTCGAGTAGTTTGGGAATGCTGCTCAAAATGGGAGGTAAATTTCTTC
TAAAGCTAAATACCGGCCAGAGACCGATAGCGCACAAGTAGAGTGATCGAAAGA
TGAAAAGCACTTTGAAAAGAGGGTTAAATAGCACGTGAAATTGTTGAAAGGGAA
GCGCTTGTGACCAGACTTGCGCCGGGCTGATCATCCGGTGTTCTCACCGGTGCAC

TCTGCCCGGCTCAGGCCAGCATCGGTTCTCGCGGGGGATAAAGGCCCTGGGAAC
GTAGCTCCTCCGGGAGTGTTATAGCCCGGGGCGCAATGCCCTCGCGGGACCGAG
GTTCGCGC-
TCTGCAAGGATG???
??
??
???CTGGTCACCCTGGTCCAAGGGT
TGCGGCGGAAGAATGTCATTTCGTTTGAGGTGTCGCTCGTCAGGGACATCCGCGA
CCGCGAGTTCAAGATCTTCTCGGATGCTGGCCGTGTCATGAGGCCACTGTTCACC
GTGGAGCAAGAGCACAACAGCGAGAGCGGCGCCGAGGTGGCCAGCTGATTCTC
AACAAGGAACACATCAACCGACTGGAGACGGACAAGGAGTTGGGCAAATACCAT
CCCGATTACTGGGGTTGGCAGGGCTTGTTAAAGTCGGGTGCCATCGAGTACCTGG
ACGCCGAGGAGGAGGAGACGGTCATGATCTGCATGACTCCCGAGGACCTCGACA
AGTTCCGTTACCGCAAAATGGGCTTCATCATTGAAGACAATTCTGGTC---
AGGGTAACAACAGGATCAAGACGAAGCCGAACCCGACGACTCACATGTACACCC
ACTGCGAGATCCACCCCAGCATGTTGCTCGGCATCTGCGCGAGCATCATTCCGTT
CCCAGACCACAACCAG??GCGTC-TGGGAAGC--TTTTTTTTGGGCCCCTGAT---
CTACCCCACACACATCGA-GACGAC-GTCG-TCCCACCAGCT-------CCCGA---
CCGAC------CGATGGCGATGGTGGCGCTGCCATGAT--GGGAAGT-------ACACG-
ATGCTGACAT--
TGTCTCTTTGCTACAGGTTCACCTTCAGACCGGCCAGTGCGTAAGTTGAACCGATT
C-GAAC-ATGGTGACCGA---------TCACATGG-TGC--GGGTG--GACTGACA-
TGAAGCT-
CCCCAGGGTAACCAAATCGGTGCCGCTTTCTGGTATGTTAATTCGAAA-
ACACCAAGTCCGTTGTAACCCATGGGCCAT---
CAATGCTGACTTCCTTCACAGGCAGACCATCTCTGGCGAGCACGGCCTCGACAGC
AATGGCGTGTACGTGACTGTCGCCGATTCCGAT--CAATTAAGCCC---
TCGCTCACCGCTAC-
AATAGGTACAATGGCACCTCCGAGCTCCAGCTCGAGCGCATGAACGTCTACTTCA
ACGAGGTAAGTCGGCCT--CACACACCTT-TACTGTGTCACGA---CAATCATTTGC-
GTTTGCTGACGAA-TGAA----
TCTCTCCAGGCTTCCGGCAACAAGTATGTCCCTCGTGCCGTCCTGGTCGACCTGGA
GCCCGGCACCATGGATGCCGTCCGCGCTGGTCCCTTCGGTCAGCTCTTCCGCCCTG
ACAACTT

>Subramaniula_thielavioides_CBS_122_78

ATTACAGAGTTTCTATAACTCCCCAAACCATTGTGAACCTTACC-
TTCTACCGTTGCTTCGGCGGGCGGG-CCGGCCCC-AGGGC------------------
CGCGCCCCCCAGGCCCCCTTC------------------
GCGGGGCGCCCGCCGGAGGAAACCCAAACTCTTGATATCC---
ATGGCCTCTCTGAGTCTTCTGTACTGAA-
TAAGTCAAAACTTTCAACAACGGATCTCTTGGTTCTGGCATCGATGAAGAACGCA
GCGAAATGCGATAAGTAATGTGAATTGCAGAATTCAGTGAATCATCGAATCTTTG
AACGCACATTGCGCCCGCCAGTATTCTGGCGGGCATGCCTGTTCGAGCGTCATTT
CAACCATCAAGCCCC-GGGCTTGTGCTGGGGACCTGCG----------GCTG-
CCGCAGGCCCTGAAAACCAGTGGCGGGCTCGCT-GTCACACCGAGCGTAGTAG-
ATTCTCATCTCGCTCAGGGCG-TGCTGCGGGTT--

CCGGCCGTAAAACAGCCTTATTTACCCAAGGTTGACC???????????????????????????
???GTGAAGCGGCAACA
GCTCAAATTTGAAATCTGGCTTCGGCCCGAGTTGTAATTTGCAG-
AGGAAGCTTTAGGCGCGGCACCATCTGAGTCCCCTGGAACGGGGCGCCATAGAG
GGTGAGAGCCCCGTATAGATGGACGCTAAGCCTGTGTAAAGCTCC-
TTCGACGAGTCGAGTAGTTTGGGAATGCTGCTCAAAATGGGAGGTAAATTTCTTC
TAAAGCTAAATACCGGCCAGAGACCGATAGCGCACAAGTAGAGTGATCGAAAGA
TGAAAAGCACTTTGAAAAGAGGGTTAAATAGCACGTGAAATTGTTGAAAGGGAA
GCGCTTGTGACCAGACTTGCGCCGGGCTGATCATCCGGTGTTCTCACCGGTGCAC
TCTGCCCGGCTCAGGCCAGCATCGGTTCTCGCGGGGGGATAAAGGCCCTGGGAAC
GTAGCTCCTCCGGGAGTGTTATAGCCCAGGGTGTAATGCCCTCGCGGGGACCGAG
GTTCGCGCATCTGCAAGGATGCTGGCGTAATGGTCATCAGCGACCCGTCTTGAAA
CACGGACCAAGGAGTCAAGGTTTTGCGCGAGTGTTTGGGTGTAAAACCCGCACGC
GTAATGAAAGTGAACGTAGGTGAGAGCTTCGGCGCATCATCGACCGATCCTGATG
TTTTCGGATGGATTTGAGTAGGAGCGTTAAGCCTTGGACCCGAAAGATGGTGAAC
TATGCTTGGATAGGGTGAAGCCAGAGGAAACTCTGGTGGAGGCTCGCAGCGG-
TTCTGACGTGCAAATCGATCGTCAAATCTGAGCATCTGGTCACGCTGGTCCAGGG
GCTGCGCAGGAAGAACGTCATCTCATTCGAGGTGTCGCTGGTCCGCGACATTCGC
GACCGCGAGTTCAAGATCTTCTCCGATGCGGGCCGTGTCATGAGGCCGCTCTTCA
CTGTGGAGCAAGAGCATAACGCGGAGAGCGGCGTCGAGGTCGGCCAGTTGATCC
TCAACAAAGAGCACATCGCGCGGCTGGAGGCGGACAAGGAGCTGGGCAAGTACC
ACCCGGATTACTGGGGCTGGCAGGGTCTGCTCAAGTCGGGTGCCATTGAGTACCT
CGACGCCGAGGAGGAAGAAACGGTCATGATTTCCATGACGCCCGAGGATCTCGA
CAAGTTCCGGTACCGCAAGATGGGCTTCGTCGTCGAGGACAATTCCGGCC---
A???
???ACGCGTC-AGGGAAGC----
TTTTTTGGGCCCCTGAT---CTACCC--CACGCGCGAA-GACGAC-CTCA-
CCCCATGAGCC------TCCCGA---CCAGC-------
CCAAACGCGACAGATGTCGCCGTTGT---GGAAGA-------GTATG-ATGCTGACGC--
TACTTCTCAACTATAGGTCCACCTCCAGACCGGCCAGTGCGTAAGTAGAATCGGT
TCAGAAACCCGGCGACCGA---------CCAGGTGG-TGC--AGGTG--GACTGACA-
GCAAG----TTCAGGGTAACCAAATCGGTGCTGCTTTCTGGTAAGTTCGGCGTCAA-
CAACAAGCACCGACACGCAGAA-AAACAAT---CGAGACTGACCTTC-
ATCCAGGCAAACCATCTCTGGCGAGCACGGCCTCGACAGCAATGGCGTGTACGTG
ACCAACGCTGACCCTGGC--CGATCGAACCA---CCGCTCACCGCTTG-
AATAGGTACAATGGCACTTCGGAGCTCCAGCTCGAGCGCATGAACGTTTACTTCA
ACGAGGTGGGTTGGCTT--GACGCCCCAT---TCGACCATC-----TGGGTGGGCGT-
GGTTTCTGACAGC-AGCT----
GCTCTACAGGCCTCCGGCAACAAGTATGTTCCTCGCGCCGTCCTCGTCGACTTGG
AGCCCGGCACCATGGATGCCGTCCGTGCCGGTCCTTTCGGCCAGCTCTTCCGCCCC
GACAACTT

>Tengochaeta_nigropilosa_CBS_639_83

ATTACAGAGTTGC-AAAACTCCC-AAACCATTGTGAACGTTACC-
TTCAACCGTTGCTTCGGCGGGCGGCCCC----------------------------
GGGTCACCCCGGGGCCCCTGGGCCCC--------------
CTGGGCGCCCGCCGGAGGTCACC-AAACTCTTGATAATTT--

ATGGCCTCTCTGAGTCTTCTGTACTGAA-
TAAGTCAAAACTTTCAACAACGGATCTCTTGGTTCTGGCATCGATGAAGAACGCA
GCGAAATGCGATAAGTAATGTGAATTGCAGAATTCAGTGAATCATCGAATCTTTG
AACGCACATTGCGCCCGCCAGTATTCTGGCGGGCATGCCTGTCCGAGCGTCATTT
CAACCATCAAGCCCC-AGGCTTGTGTTGGGGACCTGCG----------
GCTGTCCGCAGGCCCTGAAAACCAGTGGCGGGCTCGCT-
GTCACCCCGAGCGTAGTAG---TATTATCTCGCTAAGGGCG-TGCTGCGGGTT--
CCGGCCGTTAAACGACCTT-
CATAACCCAAGGTTGACC???
???
???
???
???
???
???
???
???
???
??CTGGTGACATTGGTC
CAAGGGTTGCGGAGGAGGAACGTCATCTCCTTCGAAGTCTCACTCGTCCGGGACA
TCCGCGACCGCGAGTTCAAGATTTTCTCCGACGCCGGGCGCGTCATGAGGCCGCT
GTACACAGTGGAGCAAGAGCACAATGCAGAGAGCGGTGCCGAGGTGGGCCAGTT
GATTCTCAACAAAGAACACATTACGCGGTTGGAGACAGACAAGGAGTTGGGCAA
ATACCACCCCGACTACTGGGGCTGGCAAGGGCTGTTGAAGTCGGGCGCCATCGAG
TACCTGGATGCCGAGGAAGAGGAGACAGTCATGATTTCTATGACTCCCGAAGACC
TCGACAAGTTCCGGTACCGCAAGATGGGTTTCGCGGTGGAAGATAACTCGGGGC--
-
AAGGAAACAATAGGATCAAGACGAAACCGAACCCGACCACCCACATGTACACGC
ACTGCGAGATCCACCCCAGCATGCTGCTCGGCATCTGCGCGAGCATCATTCCATT
CCCCGACCACAACCAGACGCGTC-TGAGAAGC---TTTTTTTTGGCCCCTAAA---
CTACCC--CACACCTCGG-GACGAC-GTCG-TTCCACCGGCTGGC--GTCCCGA---
CCGTCT-----TGAGGGCGATGACGACGCGACCGTGAT--GGGGAGA-------GCACG-
ATGCTAACAT--
TGTTTCCCTACTACAGGTTCACCTTCAGACCGGCCAATGCGTAAGTCGGATCGATT
C-GAAC-TCGGCGGGCGACCGA-----TCAGGTGG-TGT--CGGTG--TACTGACA-
GCAAGCT-
CTCCAGGGTAACCAAATCGGTGCCGCTTTCTGGTATGTTCAGCCTCAA-
CCTCGAGCACTATCGTGCC-AA-AGTCGAT---
CGAAACTAACTTTCCCTCCAGGCAGACTATCTCTGGCGAGCACGGCCTCGACAGC
AATGGCGTGTACGTGACCGTCGCCGATTCCCGCC-CGCTTAATCCC---
TCGCTCACCGCTCC-
GATAGGTACAATGGCACTTCCGAGCTCCAGCTCGAGCGTATGAACGTCTACTTCA
ACGAGGTGAGTCTGAGCA-TACACACCTT-CGTCGGGTAAT-----GGAACGACCGT-
GGTCGCTAACAAC-AAAT----
ATTTCGCAGGCGTCCGGCAACAAGTATGTCCCTCGTGCCGTCCTTGTCGACTTGGA
GCCCGGCACCATGGATGCCGTCCGAGCCGGTCCCTTCGGCCAGCTCTTCCGCCCC
GACAACTT

>Jugulospora_vestita_CBS_135.91

???????????????????????CACACCATCGTGAACGTCACC---
GCATCGTTTCTTCGGCGG-GGCCCC---------------------------------
CCACCGGGGCCGCGCCTGCCCCCTC------
GCGGGGCGGCAGCCCGCCGGAGGCGTCC-AAACTCTCAGCATCTA--
GTGGCATCTCTGAGTAGCTTACAAAA---
TAAGTCAAAACTTTCAACAACGGATCTCTTGGCTCTGGCATCGATGAAGAACGCA
GCGAAATGCGATAAGTAATGCGAATTGCAGAATCCAGTGAGTCATCGAATCTTTG
AACGCACATTGCGCCCGCCAGTATTCTGGCGGGCATGCCTGTCCGAGCGTCATTT
CCACCATCAAGCCCTGCGGCTTGTGTTGGGGCCCTGCG----------
GCCGCCCGCAGCCCCCGGAATGCAGTGGCGGGCTCGTT-
GTCACCCCGAGTGCAGTAA----TGCTCTTCTCTCGCGGCGTGGCGGCGGGTT--
CCGGCCGTGAAA-
CCAACCAAACTCATCAAGGTTGACCTCGGATCAGGTAGGAATACCCGCTGAACTT
AAGCATATCAATAAGCGGAGGAAAAGAAACCAACAGGGATTGCCC-
CAGTAACGGCG-
AGTGAAGCGGCAACAGCTCAAATTTGAAATCTGGCCTCGGCCCGAGTTGTAATTT
GCAG-
AGGAAGCTTCTGGTGCGGTCTGTCCCGAGTCCCCTGGAACGGGGCGCCGGAGAG
GGTGAGAGCCCCGTACGGACGGATACCAATCCTGTGTGAAGCTCC-
TTCGACGAGTCGAGTAGTTTGGGAATGCTGCTCAAAATGGGAGGTAAATTCCTTC
TAAAGCTAAATACCGGCCAGAGACCGATAGCGCACAAGTAGAGTGATCGAAAGA
TGAAAAGCACTTTGAAAAGAGGGTTAAACAGCACGTGAAATTGTTGAAAGGGAA
GCGCTTGTGACCAGACTTGCGCCAGGTCGATCATCCGGTGTTCTCACCGGTGCAC
TCGGCCTGGCTCAGGCCAGCATCGGTTCCCGCGGGGGGATAAAGGCCCAGGGAA
TGTAGCTCCTCCGGGAGTGTTATAGCCCGGGGCGCAATGCCCCCGTGGGGACCGA
GGTTCGCGCATCTGCAAGGATGCTGGCGTAATGGTCATCAGCGACCCGTCTTGAA
ACACGGACCAAGGAGTCAAGGTTTTGCGCGAGTGTTTGGGTGTCAAACCCGCACG
CGTAATGAAAGTGAACGTAGGTGAGAGCTTCGGCGCATCATCGACCGATCCTGAT
GTCTTCGGATGGATTTGAGTAGGAGCGTTAAGCCTTGGACCCGAAAGATGGTGAA
CTATGCTTGGATAGGGTGAAGCCAGAGGAAACTCTGGTGGAGGCTCGCAGCGG-
TTCTGACGTGCAAATCGATCGTC????????????CTGGTCAGCTTGGTTCAGAGCCTAA
GGAGATCCAATATTATCAGTTTCGAGGTTTCCCTGGTTCGCGACATCCGAGACAG
AGAGTTCAAGATCTTCTCCGATGCAGGGCGTGTCATGAGACCGCTATTCGTCGTT
GAACAGGAGGATGACGGTGAGAGCAAGGTTGAGAAGGGCCAGCTGGTTCTTACC
AAGTCGCAAATCCTCAAACTAGAAAAAGACAAGGAGATTGGCAAATACCATCCG
GATTACTTCGGCTGGAATGGCCTCTTGAGGGAAGGCTGTGTGGAATACCTGGATG
CGGAAGAAGAGGAAACGGCGATGATCTGTATGACGCCCGAGGACCTAGACACGT
ACCGGCTAGCCAAGCTTGGGTTCAACGTCGCCGAGGAGGATCCTAGTGAAGGCA
ACAAGCGCATCAAGACTAGGCTCAACCCGACGACCCATATGTATACTCACTGCGA
GATTCACCCCAGCATGCTGCTTGGTATCTGCGCCAGCATCATTCCCTTCCCCGATC
ACAACCA???
??
??
??
??
??

??
??
??

>Pseudorhypophila_mangenotii_CBS_419.67

???????????????????????CAAACCATCGCGAACGTTACCCAGACGTCGTTGCTTCGGC
GGGC---
TCGCCCGCCGGGGGGACGCTACC-AGACTCTTGCAGTTAT--
TCGGCCTCTCTGAGTACGATTTTTAAA--
TAAGTCAAAACTTTCAACAACGGATCTCTTGGTTCTGGCATCGATGAAGAACGCA
GCGAAATGCGATAAGTAATGTGAATTGCAGAATTCAGTGAATCATCGAATCTTTG
AACGCACATTGCGCCCGCCAGTATTCTGGCGGGCATGCCTGTTCGAGCGTCATTT
CAACCTTCAAGCCC---TGCTTGTGTTGGGGTCCTGCG----------
GCTGCCCGCAGGCCCTGAAAACCAGTGGCGGGCTCGCTAGTCACACCGAGCGCA
GTAG----CACATCTCGCTCAGGGCG-TGCGGCGGGTT--CTTGCCGTTAAA-
CACCCCCCCTTTCCACAGGTTGACCTCGGATCAGGTAGGAATACCCGCTGAACTT
AAGCATATCAATAAGCGGAGGAAAAGAAACCAACAGGGATTGCCC-
TAGTAACGGCG-
AGTGAAGCGGCAACAGCTCAAATTTGAAATCTGGCTTCGGCCCGAGTTGTAATTT
GTAG-
AGGAAGCTTTTGGCGCGGCACCTACTGAGTCCCCTGGAACGGGGCGCCAGAGAG
GGTGAGAGCCCCGTATAGTGGGATGCCTAGCCTCTGTAAAGCTCC-
TTCGACGAGTCGAGTAGTTTGGGAATGCTGCTCAAAATGGGAGGTAAATTTCTTC
TAAAGCTAAATATTGGCCAGAGACCGATAGCGCACAAGTAGAGTGATCGAAAGA
TGAAAAGCACTTTGAAAAGAGGGTTAAACAGCACGTGAAATTGTTGAAAGGGAA
GCGCTTGTGACCAGACTTGCGCCCGGTTGATCATCCGGTGTTCTCACCGGTGCACT
CCTCCGGGCTCAGGCCAGCATCGGTTCTCGCGGGGGGATAAAGGCCCAGGGAAC
GTAGCTCTTCCGGGAGTGTTATAGCCCTGGGTGCAATGCCCTCGCGGGGACCGAG
GTTCGCGCATCTGCAAGGATGCTGGCGTAATGGTCATCAGCGACCCGTCTTGAAA
CACGGACCAAGGAGTCAAGGTTTTGCGCGAGTGTTTGGGTGTCAAACCCGCACGC
GTAATGAAAGTGAACGTAGGTGAGAGCTTCGGCGCATCATCGACCGATCCTGATG
TATTCGGATGGATTTGAGTAGGAGCGTTAAGCCTTGGACCCGAAAGATGGTGAAC
TATGCTTGGATAGGGTGAAGCCAGAGGAAACTCTGGTGGAGGCTCGCAGCGG-
TTCTGACGTGCAAATCGATCGTCAAATCTGAGCATCTGGTCAGCTTGGTTCAGAG
CCTAAGGAGATCCAATATTATCAGTTTCGAGGTTTCCCTGGTTCGCGACATCCGA
GACAGAGAGTTCAAGATCTTCTCCGATGCAGGGCGTGTCATGAGACCGCTATTCG
TCGTTGAACAGGAGGATGACGGTGAGAGCAAGGTTGAGAAGGGCCAGCTGGTTC
TTACCAAGTCGCAAATCCTCAAACTAGAAAAAGACAAGGAGATTGGCAAATACC
ATCCGGATTACTTCGGCTGGAATGGCCTCTTGAGGGAAGGCTGTGTGGAATACCT
GGATGCGGAAGAAGAGGAAACGGCGATGATCTGTATGACGCCCGAGGACCTAGA
CACGTACCGGCTAGCCAAGCTTGGGTTCAACGTCGCCGAGGAGGATCCTAGTGAA
GGCAACAAGCGCATCAAGACTAGGCTCAACCCGACGACCCATATGTATACTCACT
GCGAGATTCACCCCAGCATGCTGCTTGGTATCTGCGCCAGCATCATTCCCTTCCCC
GATCACAACCA???
??
??
??

??
??
??
??
??

Table S2 SMART parameters for the MS-Imaging analysis of *A. tataricus tissues.*

***A. tataricus* sample**	**SMART parameters**	
Achene zone	Step size	30 µm x 30 µm
	Total scans	74760
	Acquisition time (h)	11.51
Peduncle	Step size	20 µm x 20 µm
	Total scans	47000
	Acquisition time (h)	7.1
Leaf	Step size	20 µm x 20 µm
	Total scans	92000
	Acquisition time (h)	14
Base rosette	Step size	30 µm x 30 µm
	Total scans	78400
	Acquisition time (h)	12.1
Root	Step size	30 µm x 30 µm
	Total scans	122211
	Acquisition time (h)	18.8
Rhizome	Step size	20 µm x 20 µm
	Total scans	44100
	Acquisition time (h)	6.7

(**S**): Spot size of the MALDI source: <10 µm. Experiments conducted in Constant Speed Rastering mode, scanning velocity 2.28 mm/min for 20 µm, 3.42 mm/min for 30 µm pixel size. Total scans see table below. (**M**): Molecular identification is based on high accuracy masses as shown in Table S5. (**A**): The five compounds listed in Table S5 have been studied. (**R**): Resolution set to 140,000 at *m/z* 200. (**T**): Acquisition time see table above.

Table S3 Initial results of the screening assay for toxicity-/lethality of *T. bulbillosa* EtAcO extract. HeLa cell viability assay n = 2, *C. elegans* viability assay n = 7.

**Treatment**	**HeLa cell viability (%)**				***C. elegans* viability (%)**
***T. bulbillosa* EtAcO extract conc.**	**0.125 mg/mL**	**0.25 mg/mL**	**0.5 mg/mL**	**1 mg/mL**	**1 mg/mL (0.5 % DMSO, 24h)**
**Assay result**	43 ± 1	44 ± 1	32 ± 2	65 ± 16	73 ± 45

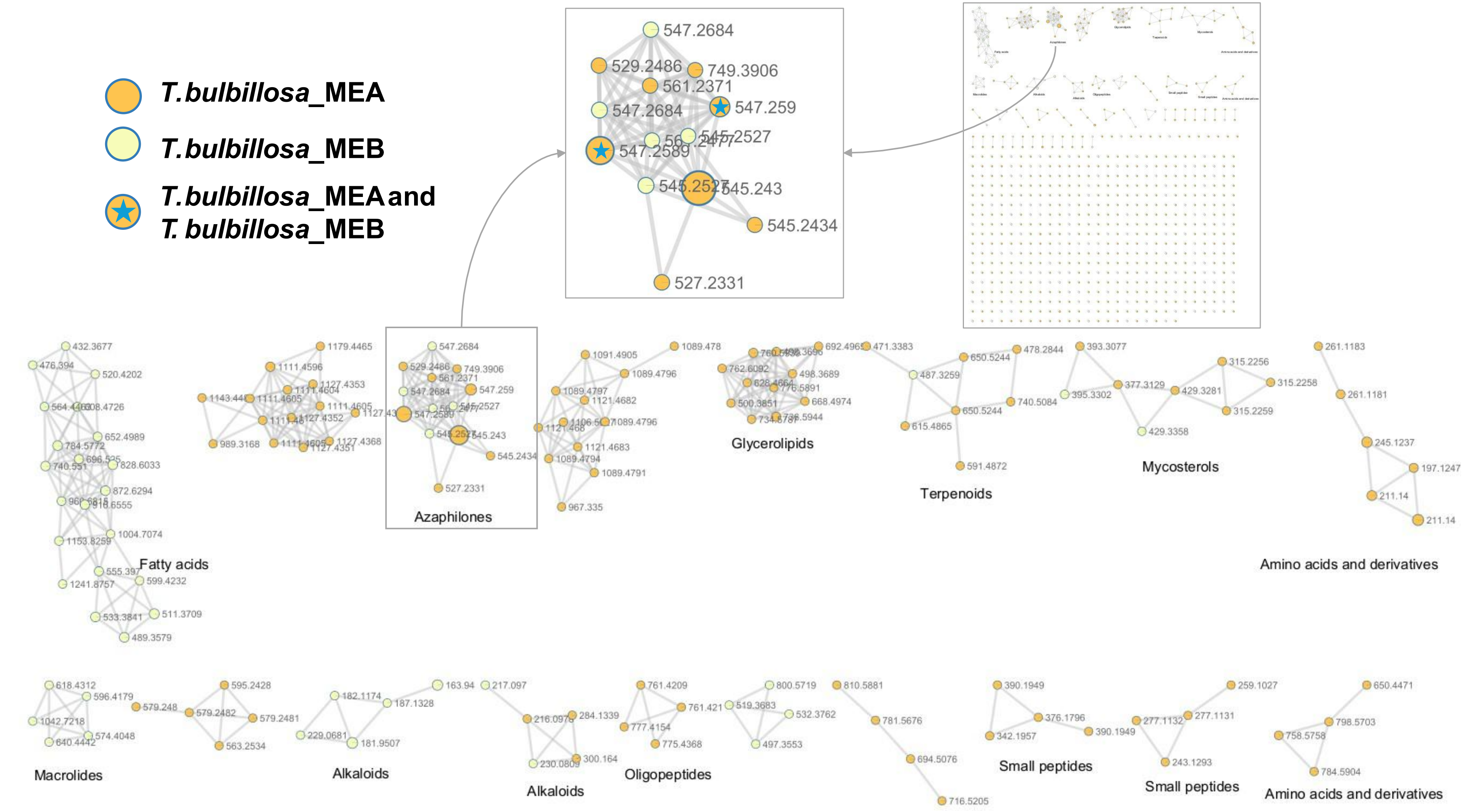

Fig. S1. GNPS network of the *T. bulbillosa* EtAcO extracts (MEB and MEA), compound classes annotated by GNPS databases and SIRIUS. The azaphilone cluster has shared nodes (*m/z* 547.2589 and 547.2590), however, the ion intensity of the compounds in the MEB extract is lower than in the MEA extract.

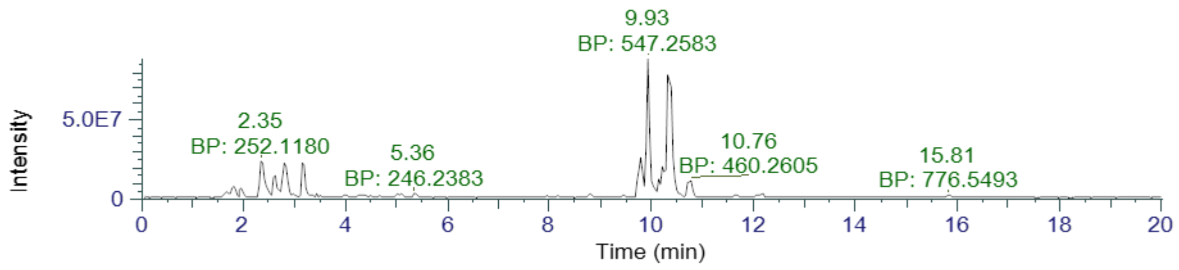

Fig. S2. HPLC-MS base peak chromatogram (pos. mode) of *T. bulbillosa* EtAcO extract (MEA medium cultivation).

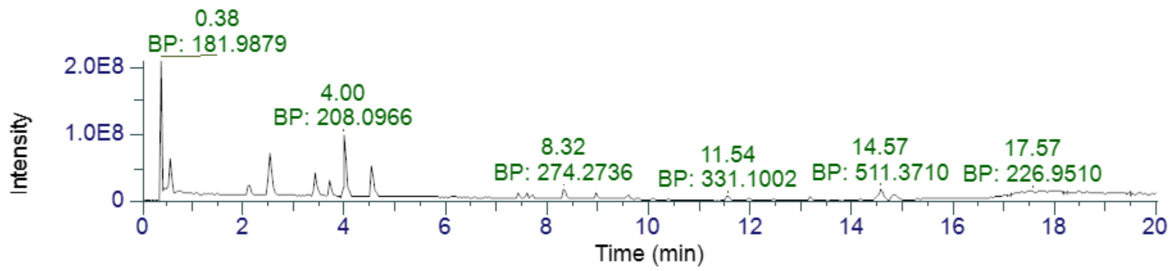

Fig. S3. HPLC-MS base peak chromatogram (positive mode) of *T. bulbillosa* EtAcO extract (MEB medium cultivation).

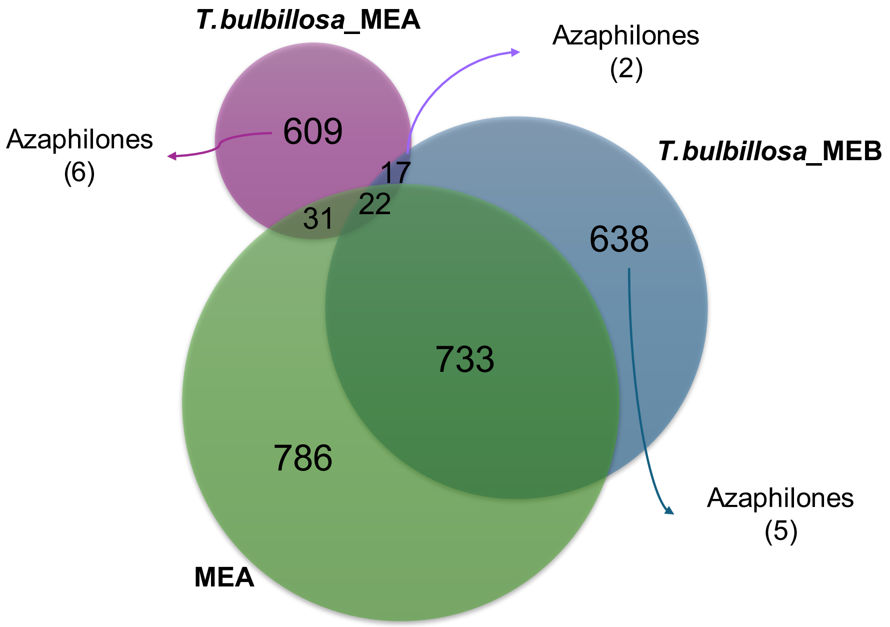

Fig. S4. Venn diagram with detected ions from *T. bulbillosa* extracts cultivated in MEA and MEB medium. MEA extract used as blank.

a

b

c

d

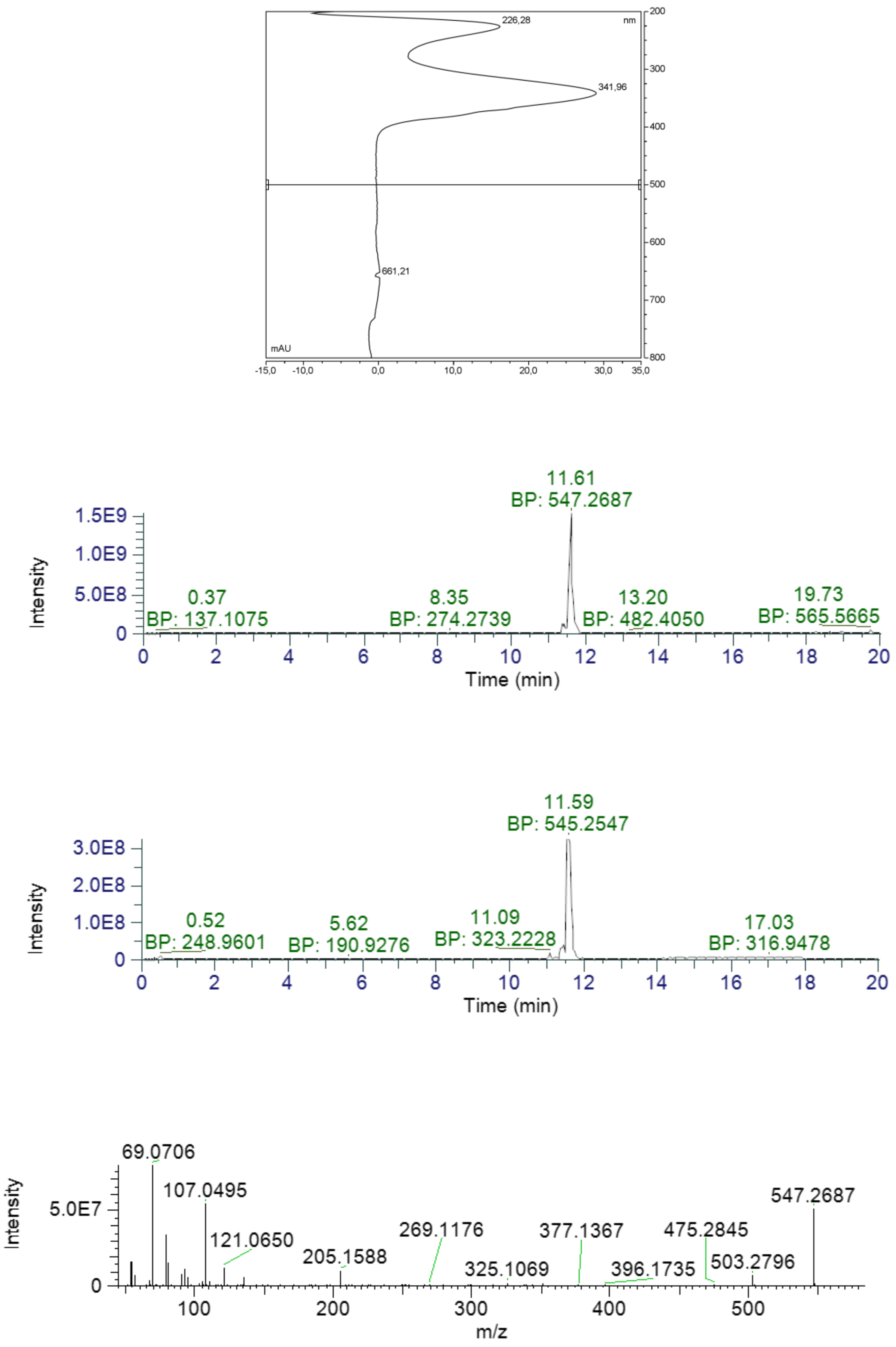

Fig. S5. HPLC data of bulbillosin A (**1**). a. UV-vis spectrum. b. ESI Full MS in pos. ion mode (BPC). c. ESI Full MS in neg. ion mode (BPC). d. MS/MS in pos. ion mode.

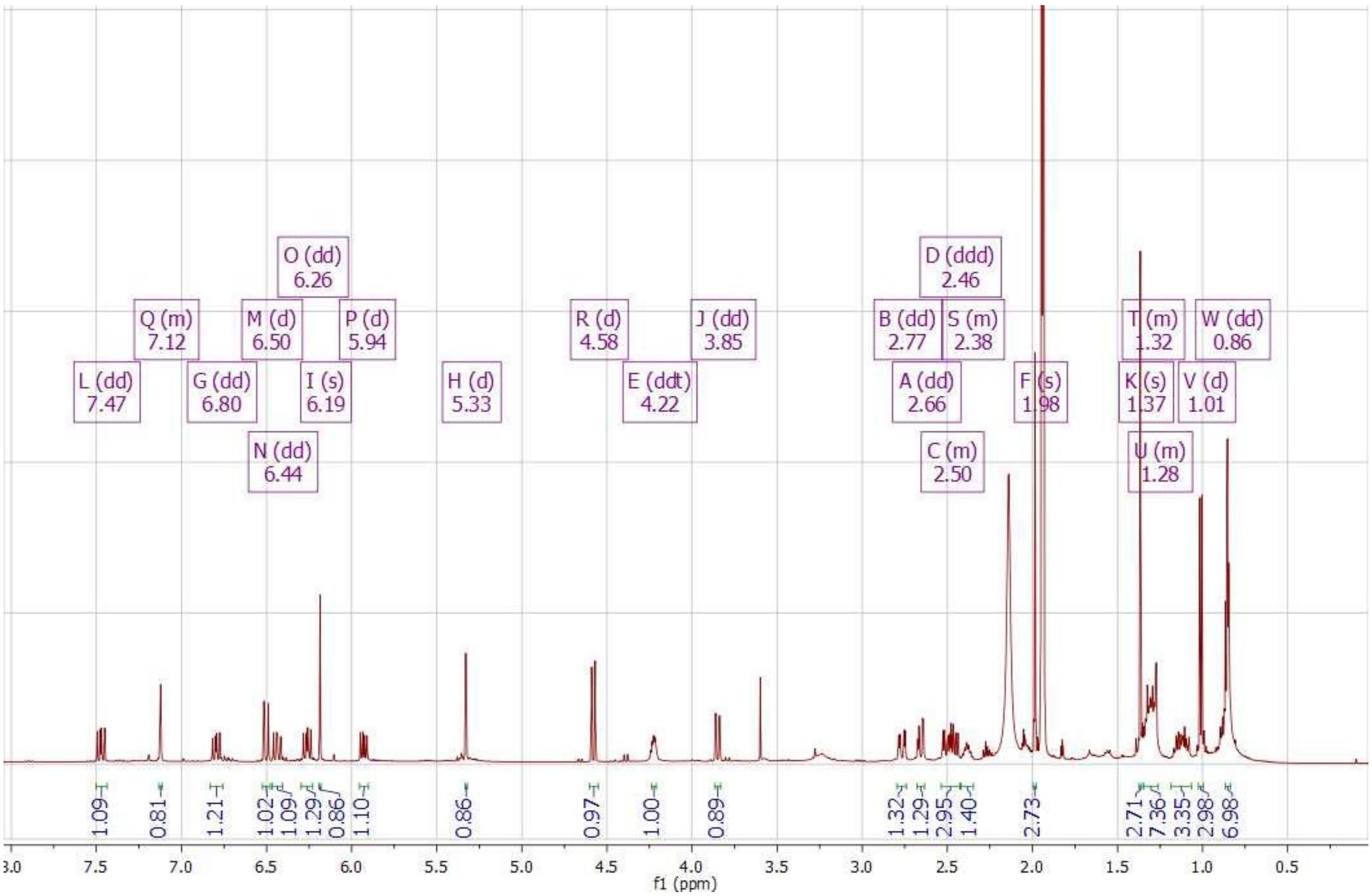

Fig. S6. Proton NMR spectrum (600 MHz, CD_3CN) of bulbillosin A (**1**).

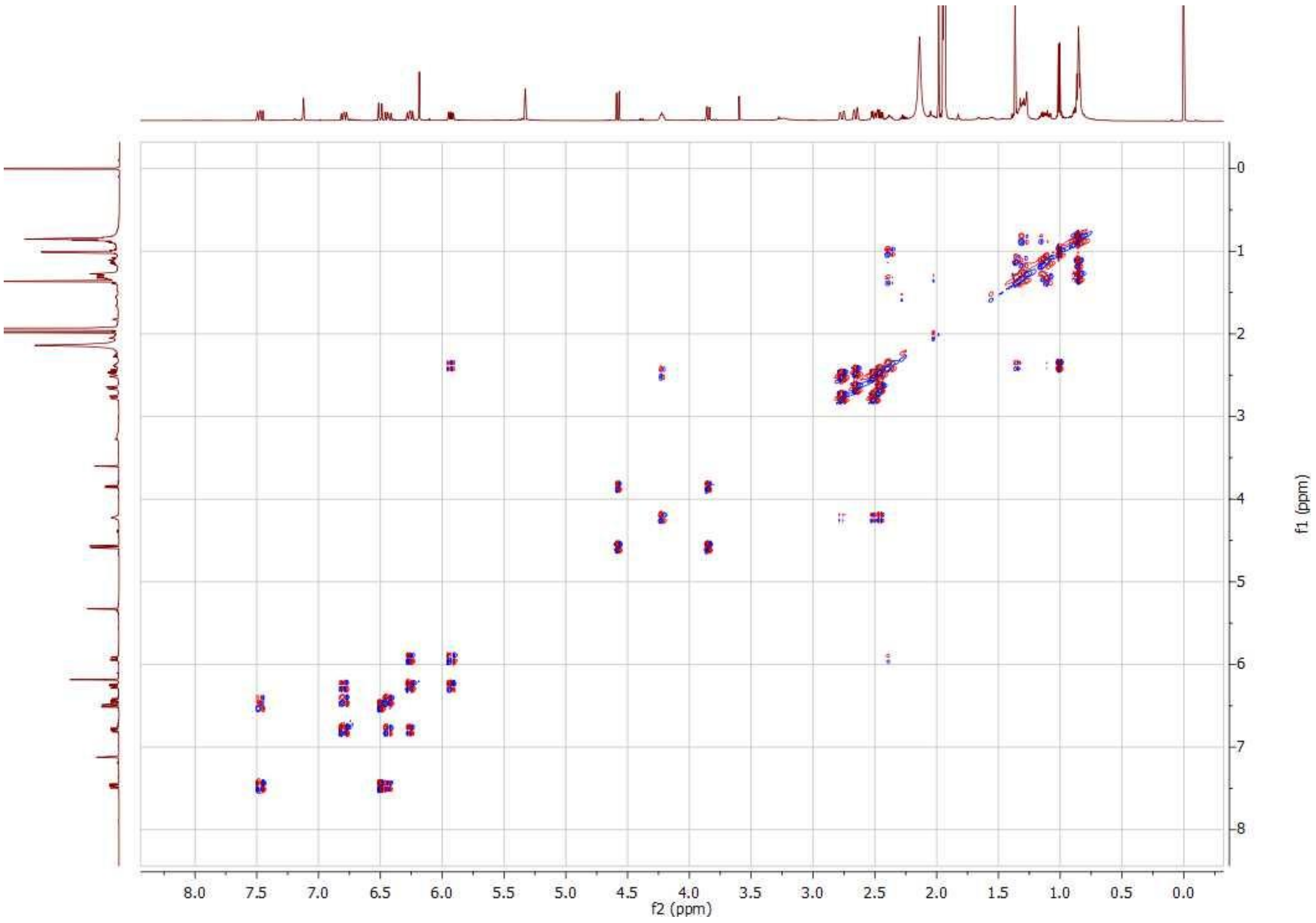

Fig. S7. COSY NMR spectrum (600 MHz, CD_3CN) of bulbillosin A (**1**).

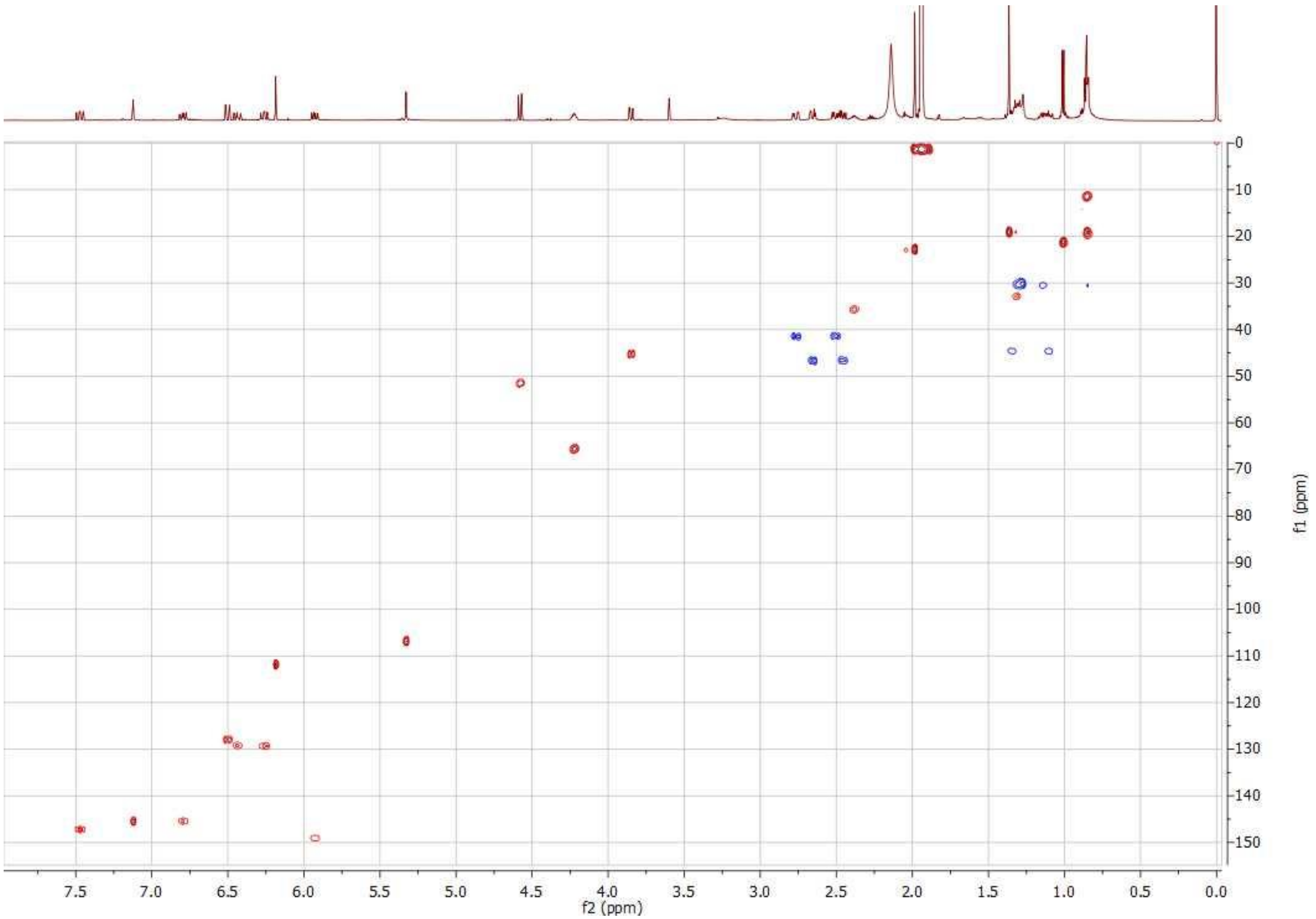

Fig. S8. DEPT-HSQC NMR spectrum (600 MHz, CD_3CN) of bulbillosin A (**1**).

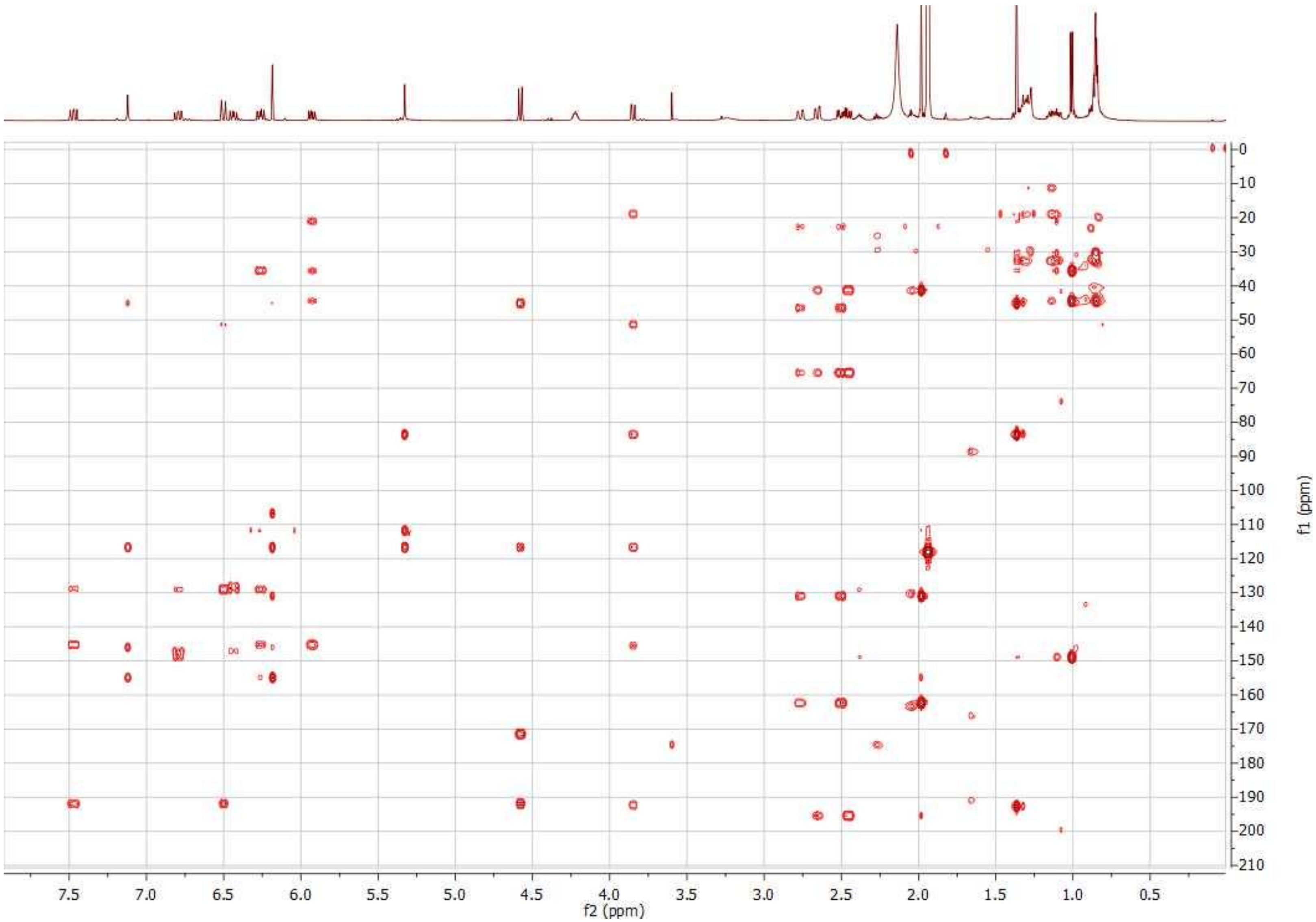

Fig. S9. HMBC NMR spectrum (600 MHz, CD_3CN) of bulbillosin A (**1**).

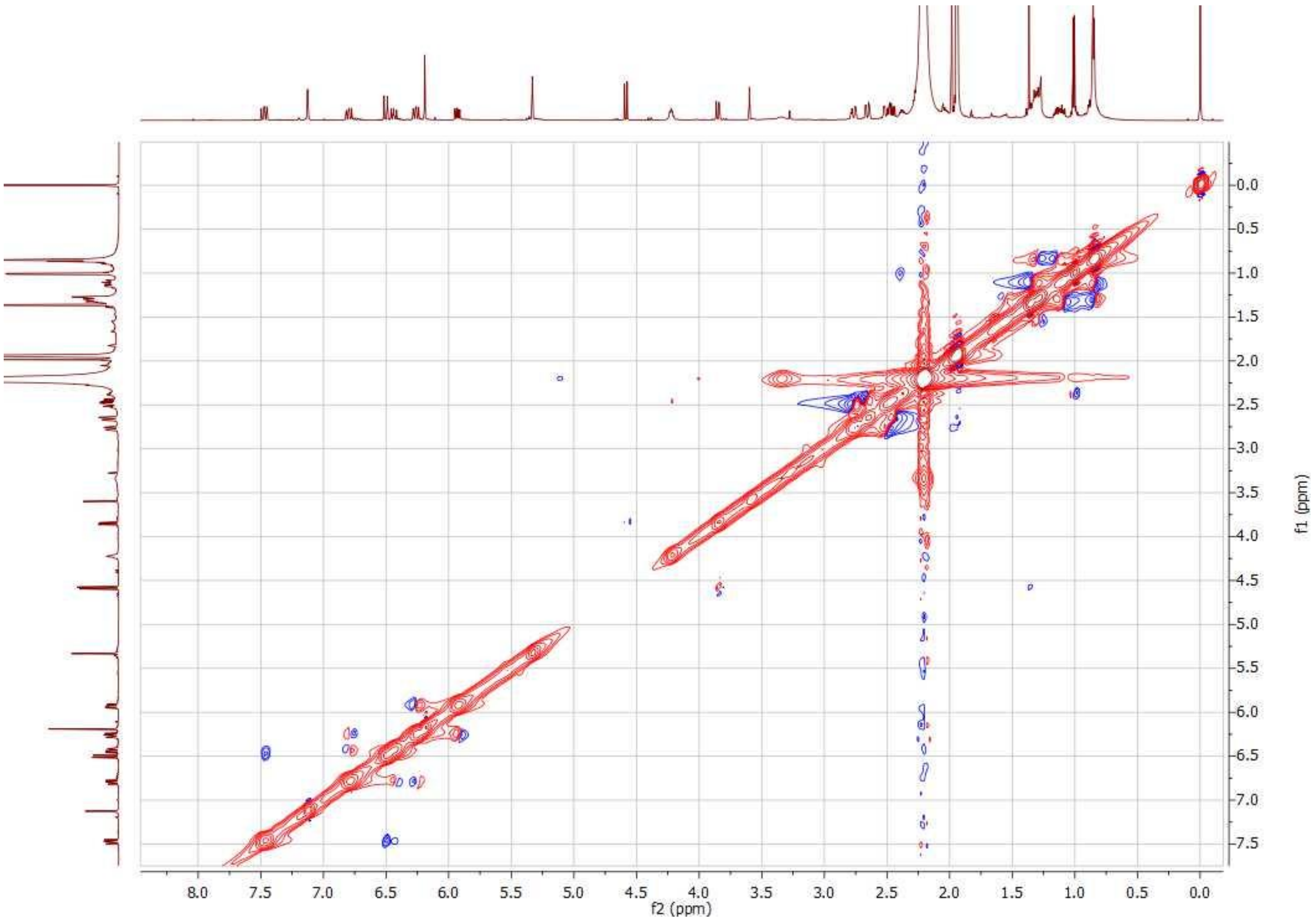

Fig. S10. NOESY NMR spectrum (600 MHz, CD_3CN) of bulbillosin A (**1**).

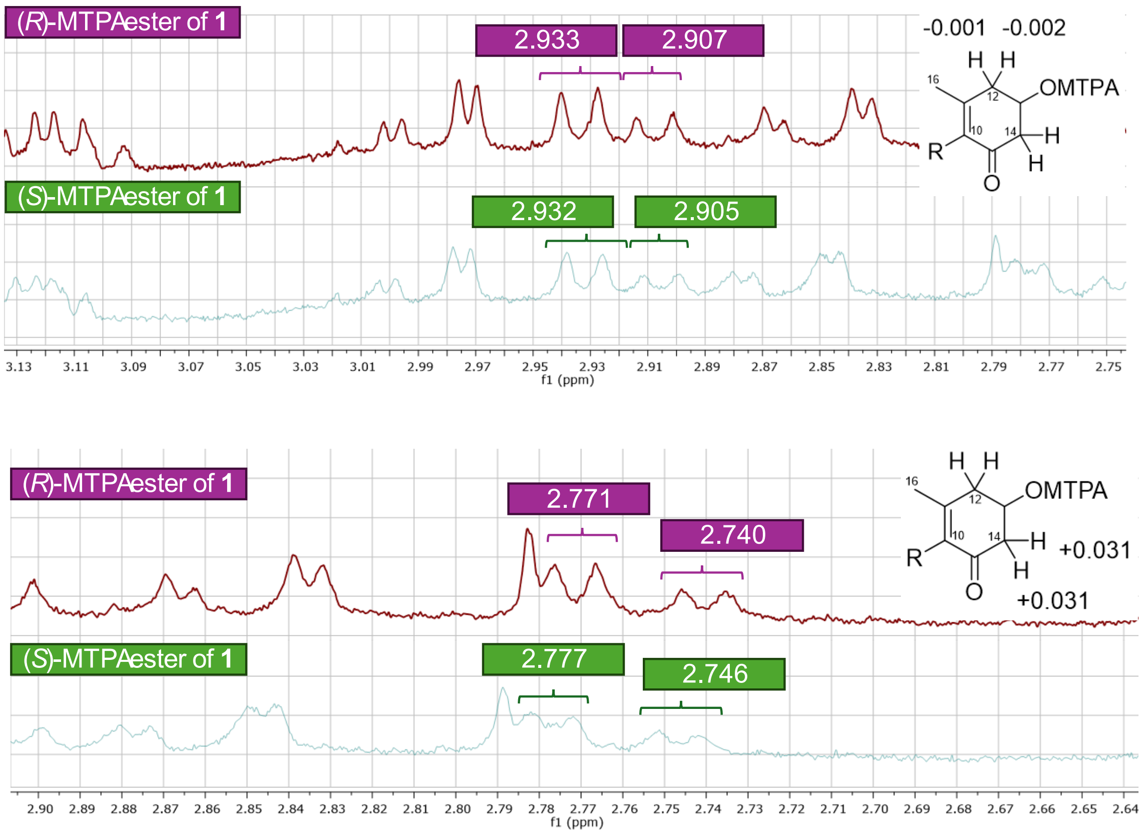

Fig. S11. Δd^{SR} values after comparison of the ^{1}H NMR spectroscopic data of the MTPA esters of bulbillosin A (**1**).

a

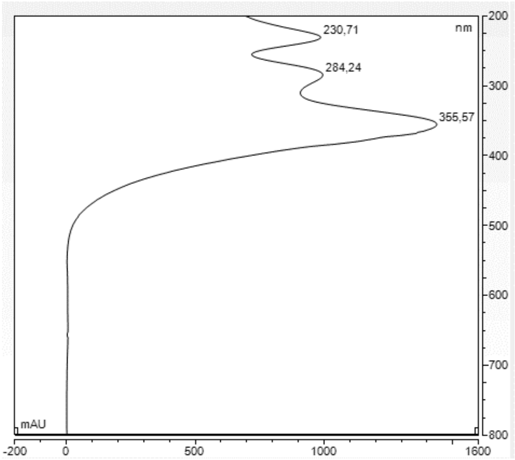

b

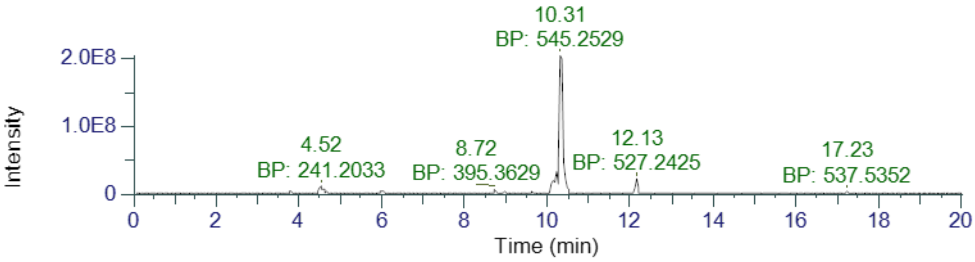

c

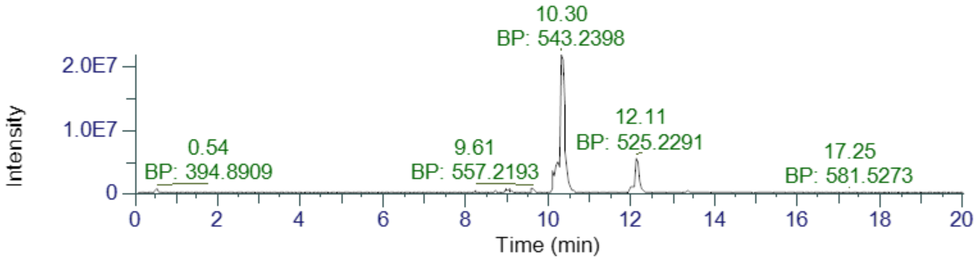

d

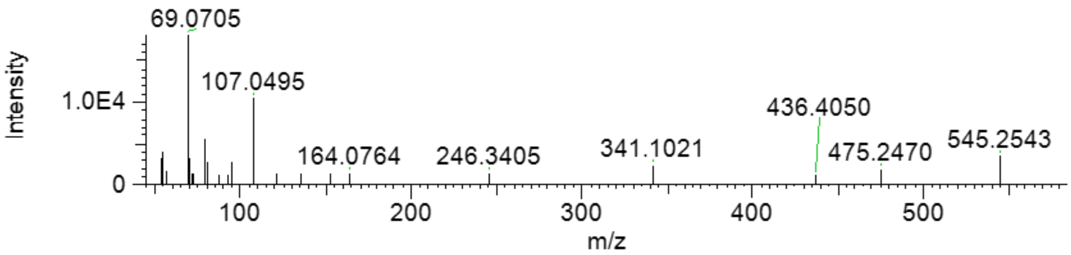

Fig. S12. HPLC-HRESI data of bulbillosin B (**2**) a. a. UV-vis spectrum. b. ESI Full MS in pos. ion mode (BPC). c. ESI Full MS in neg. ion mode (BPC). d. MS/MS in pos. ion mode.

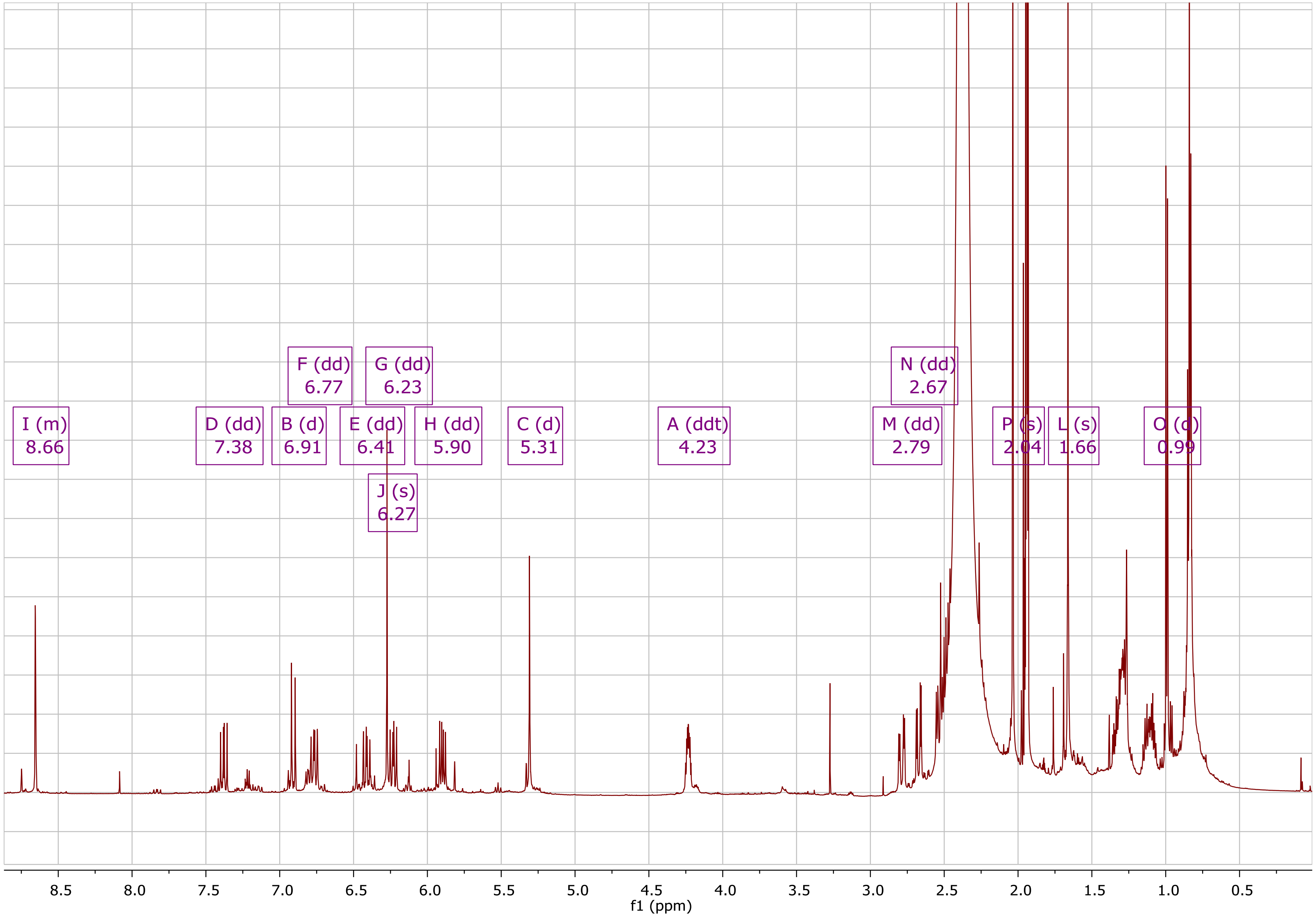

Fig. S13. Proton NMR spectrum (600 MHz, CD_3CN) of bulbillosin B (**2**).

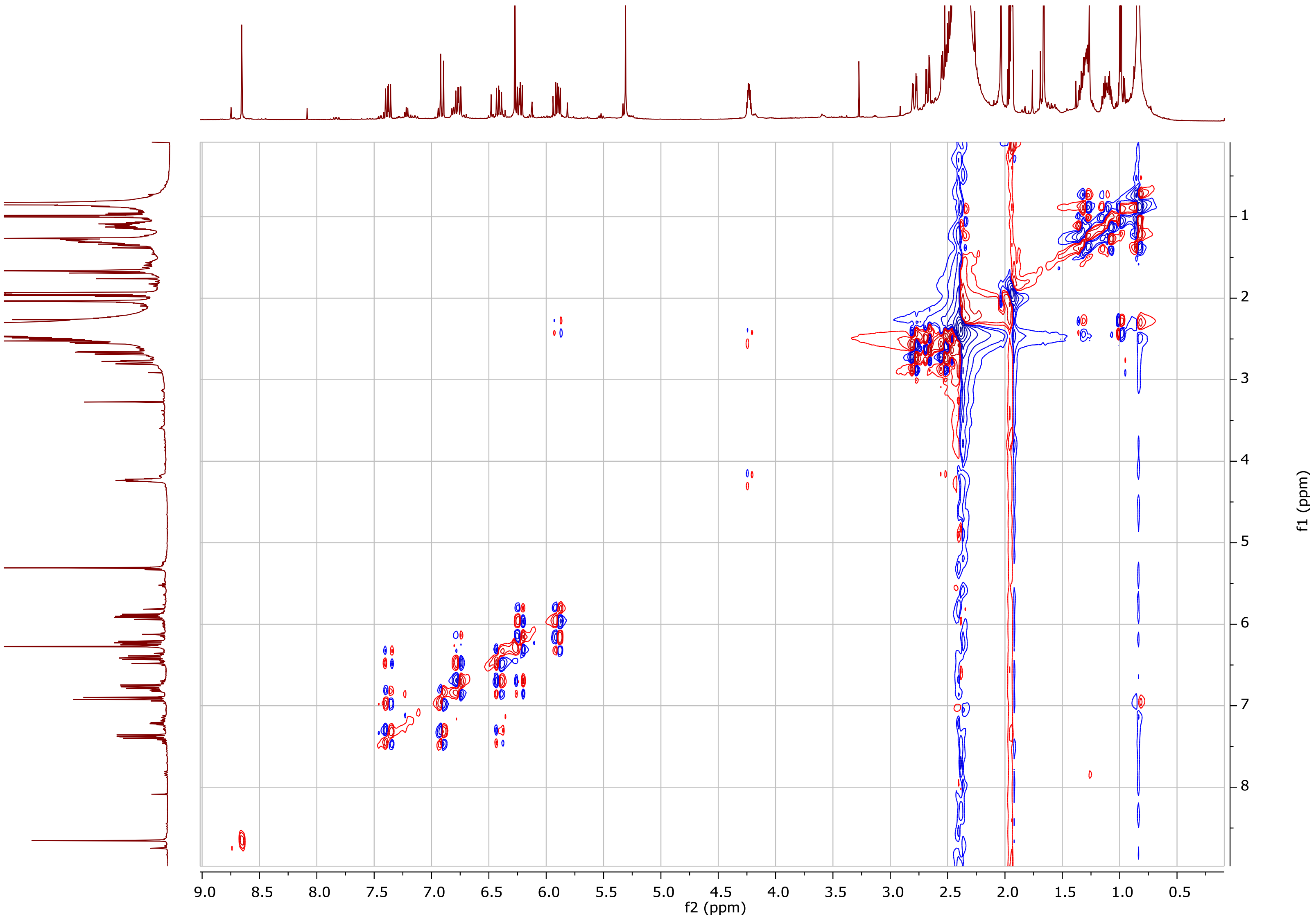

Fig. S14. COSY NMR spectrum (600 MHz, CD_3CN) of bulbillosin B (**2**).

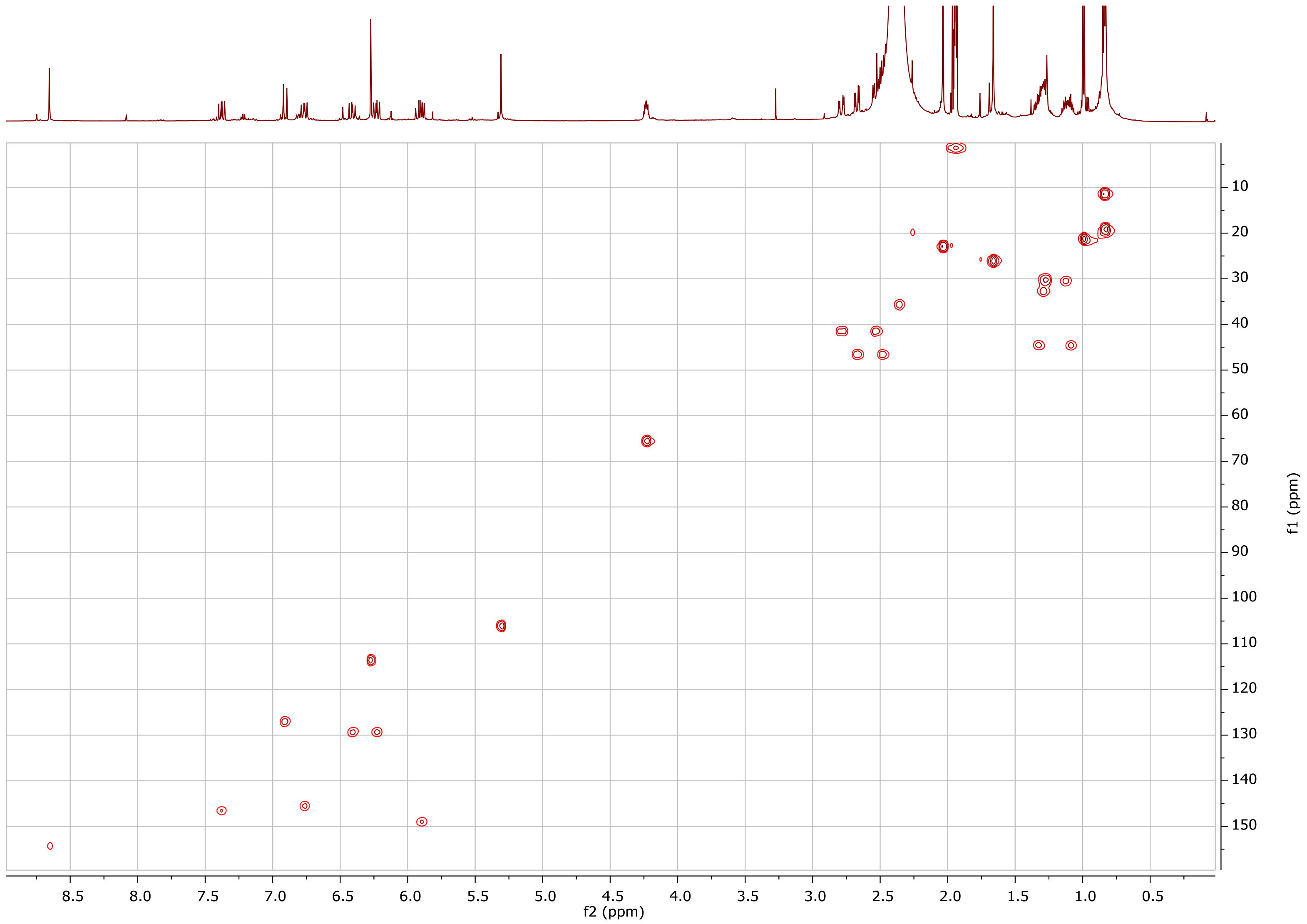

Fig. S15. HSQC NMR spectrum (600 MHz, CD_3CN) of bulbillosin B (**2**).

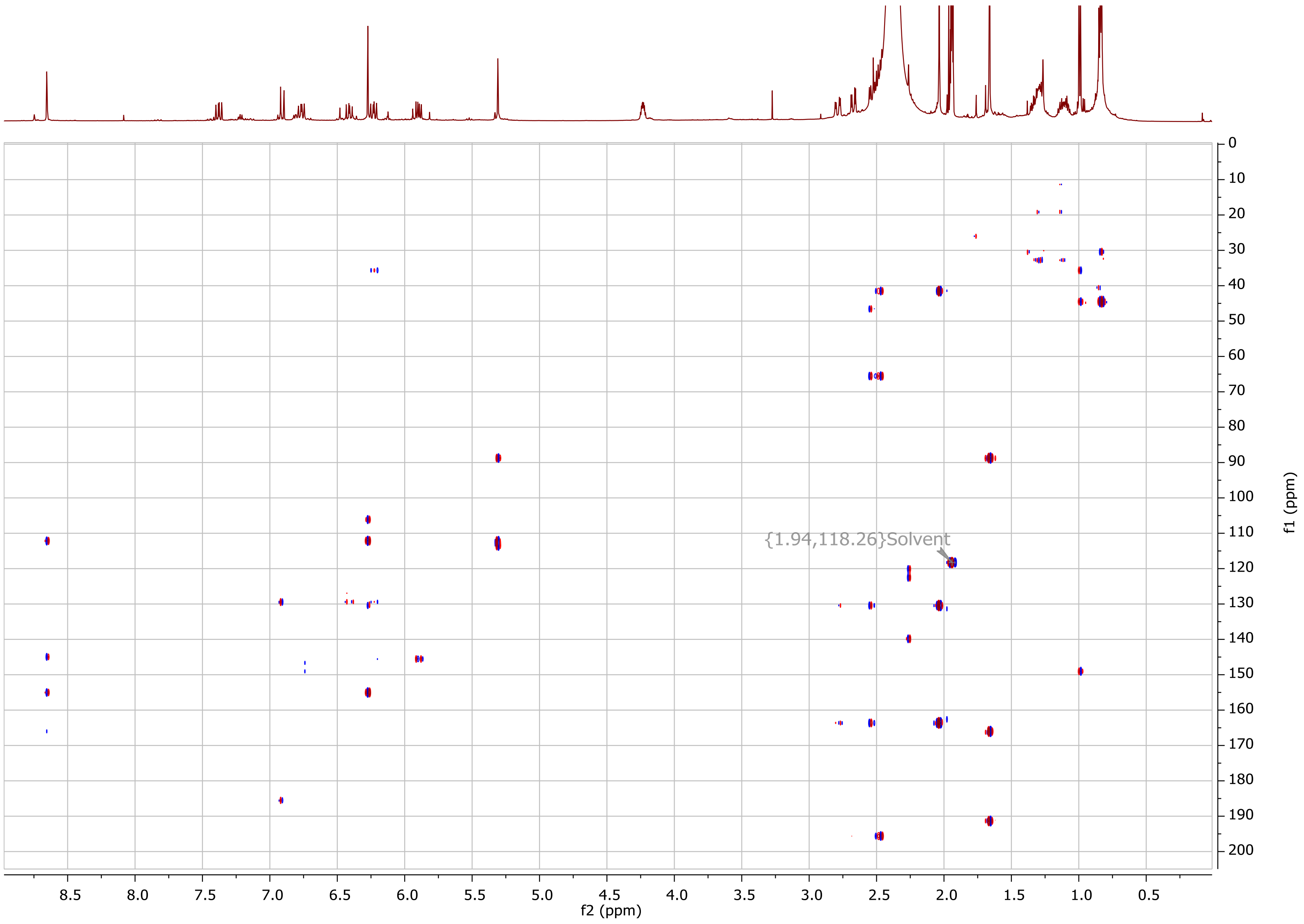

Fig. S16. HMBC NMR spectrum (600 MHz, CD_3CN) of bulbillosin B (**2**).

a

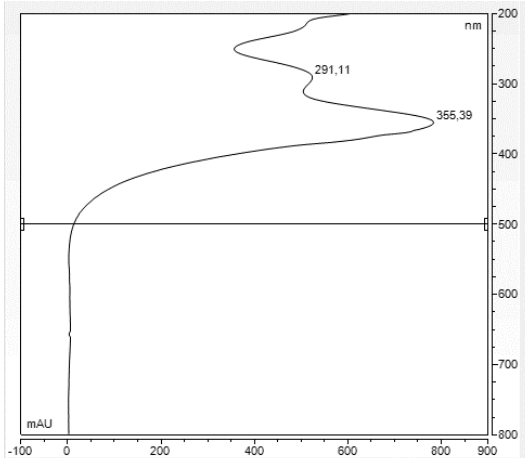

b

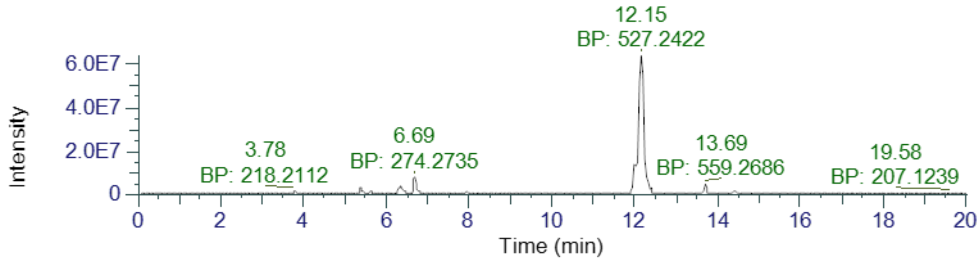

c

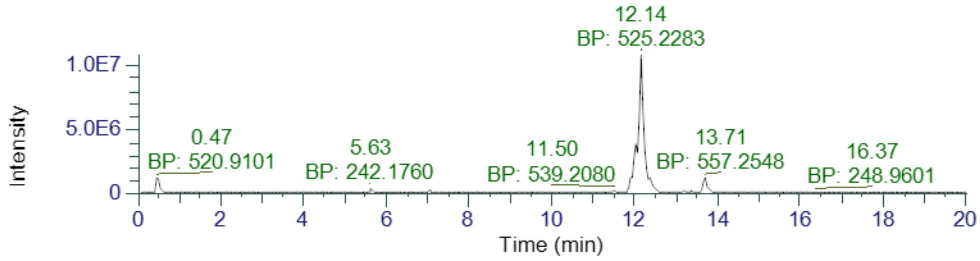

d

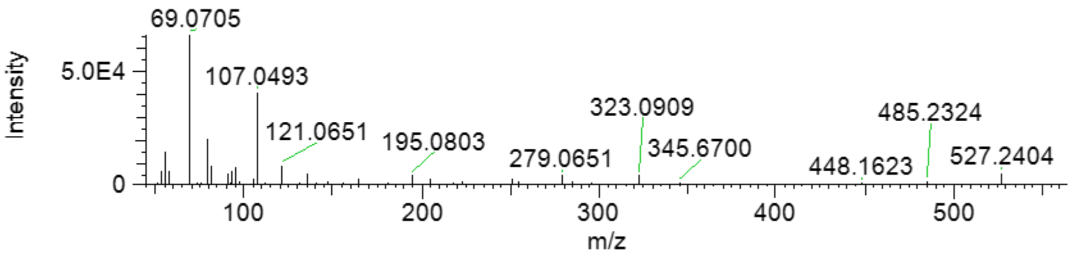

Fig. S17. HPLC-HRESI data of bulbillosin C (**3**). a. UV-vis spectrum. b. ESI Full MS in pos. ion mode (BPC). c. ESI Full MS in neg. ion mode (BPC). d. MS/MS in pos. ion mode.

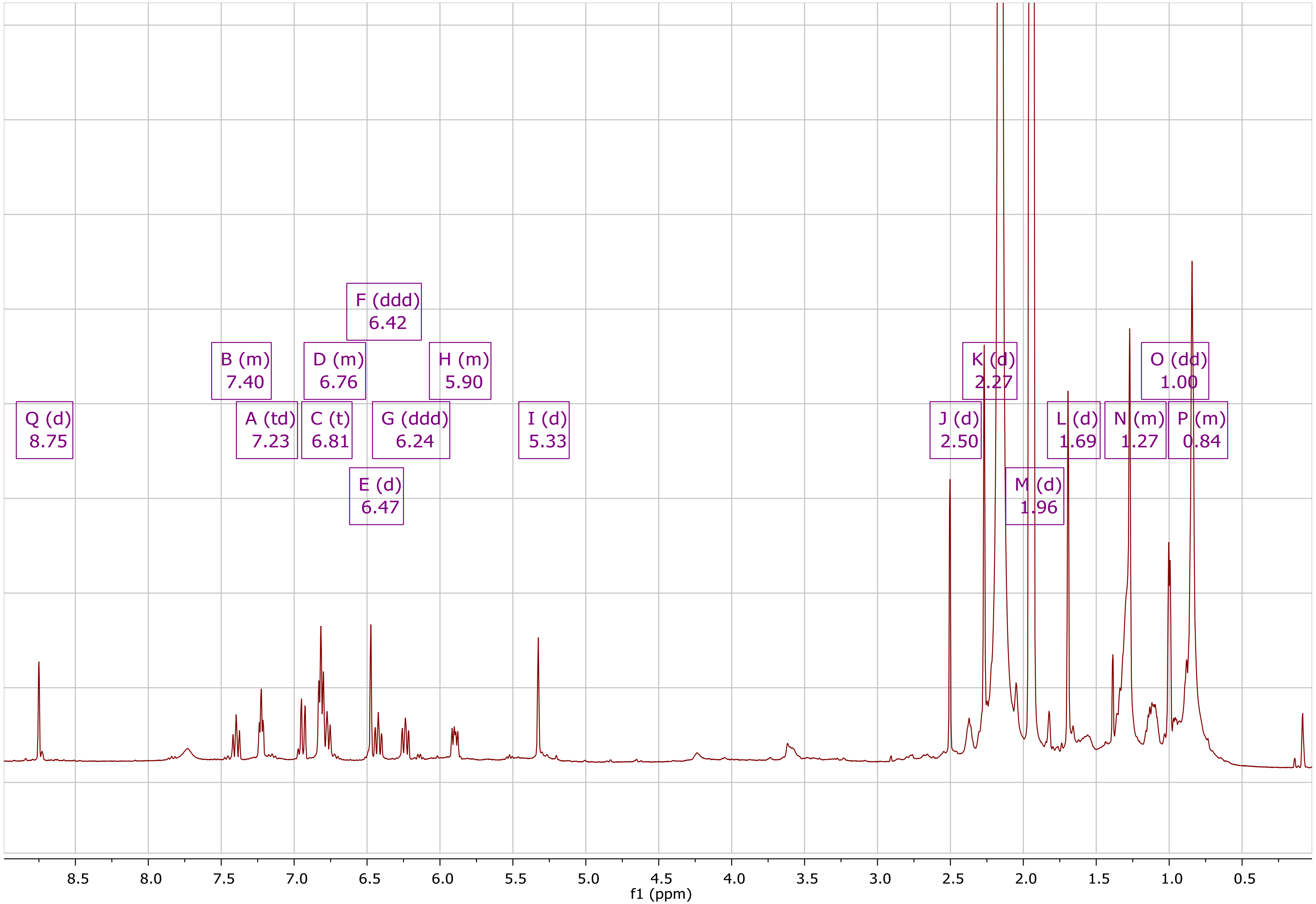

Fig. S18. Proton NMR spectrum (600 MHz, CD_3CN) of bulbillosin C (**3**).

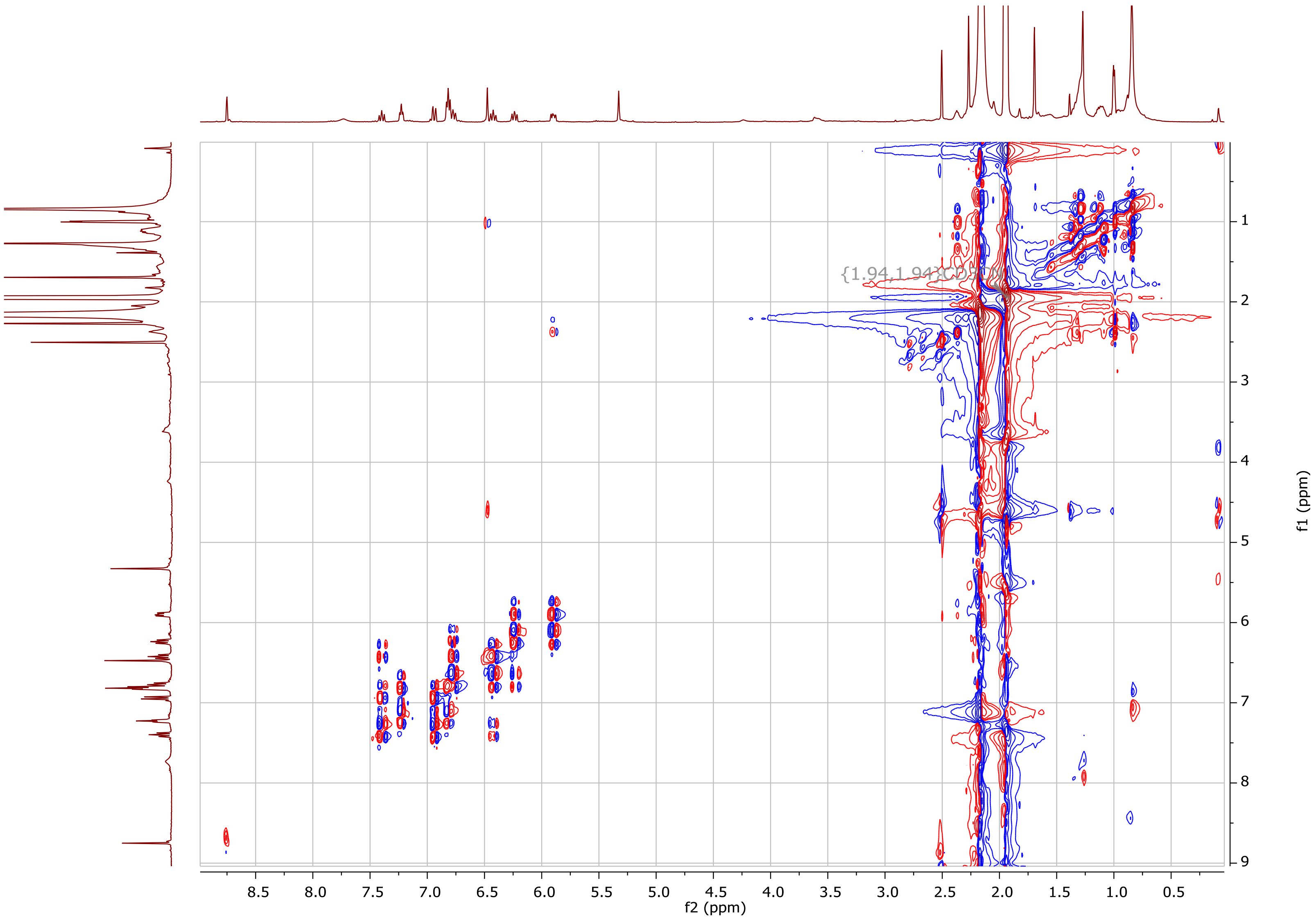

Fig. S19. COSY NMR spectrum (600 MHz, CD_3CN) of bulbillosin C (**3**).

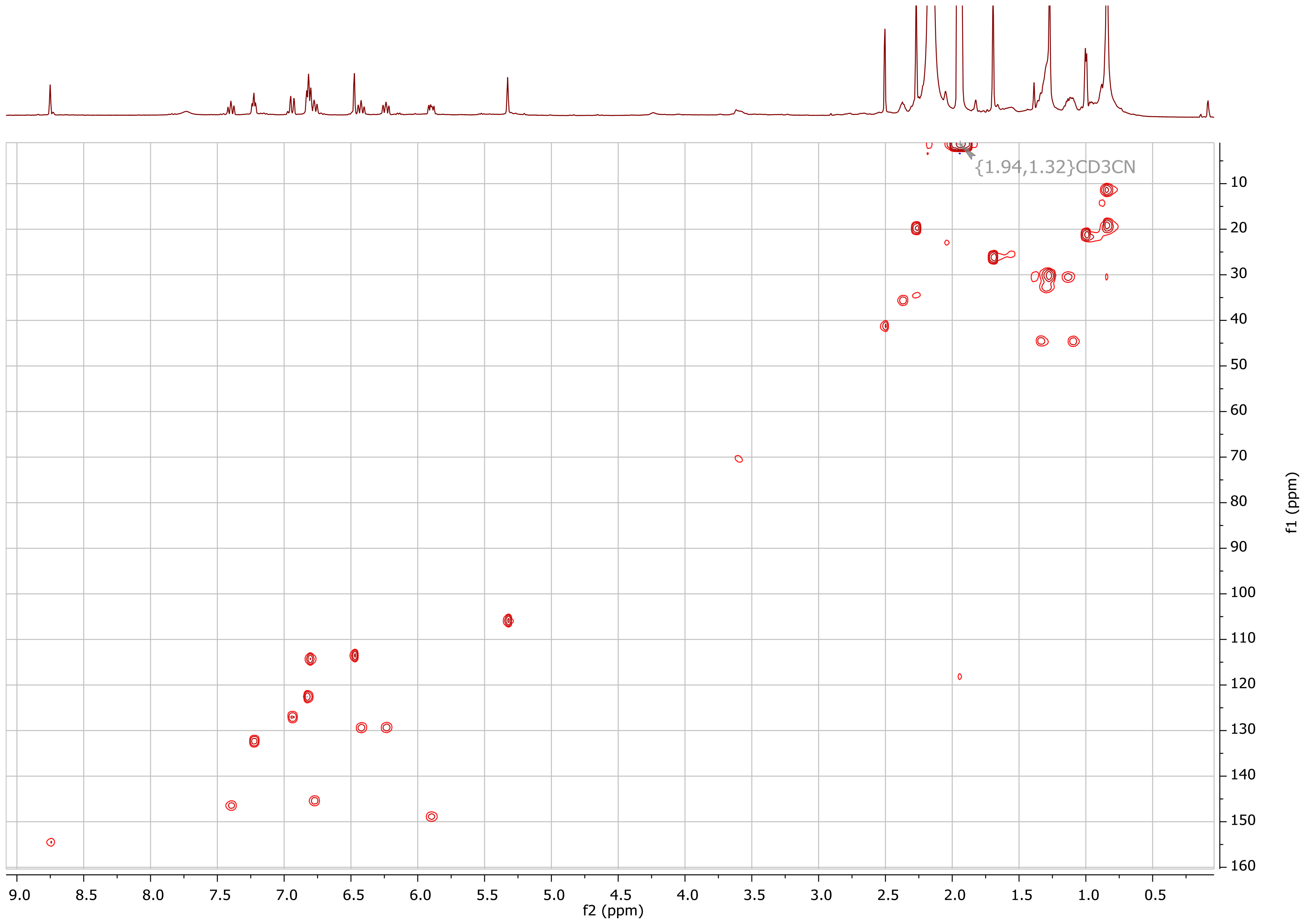

Fig. S20. HSQC NMR spectrum (600 MHz, CD_3CN) of bulbillosin C (**3**).

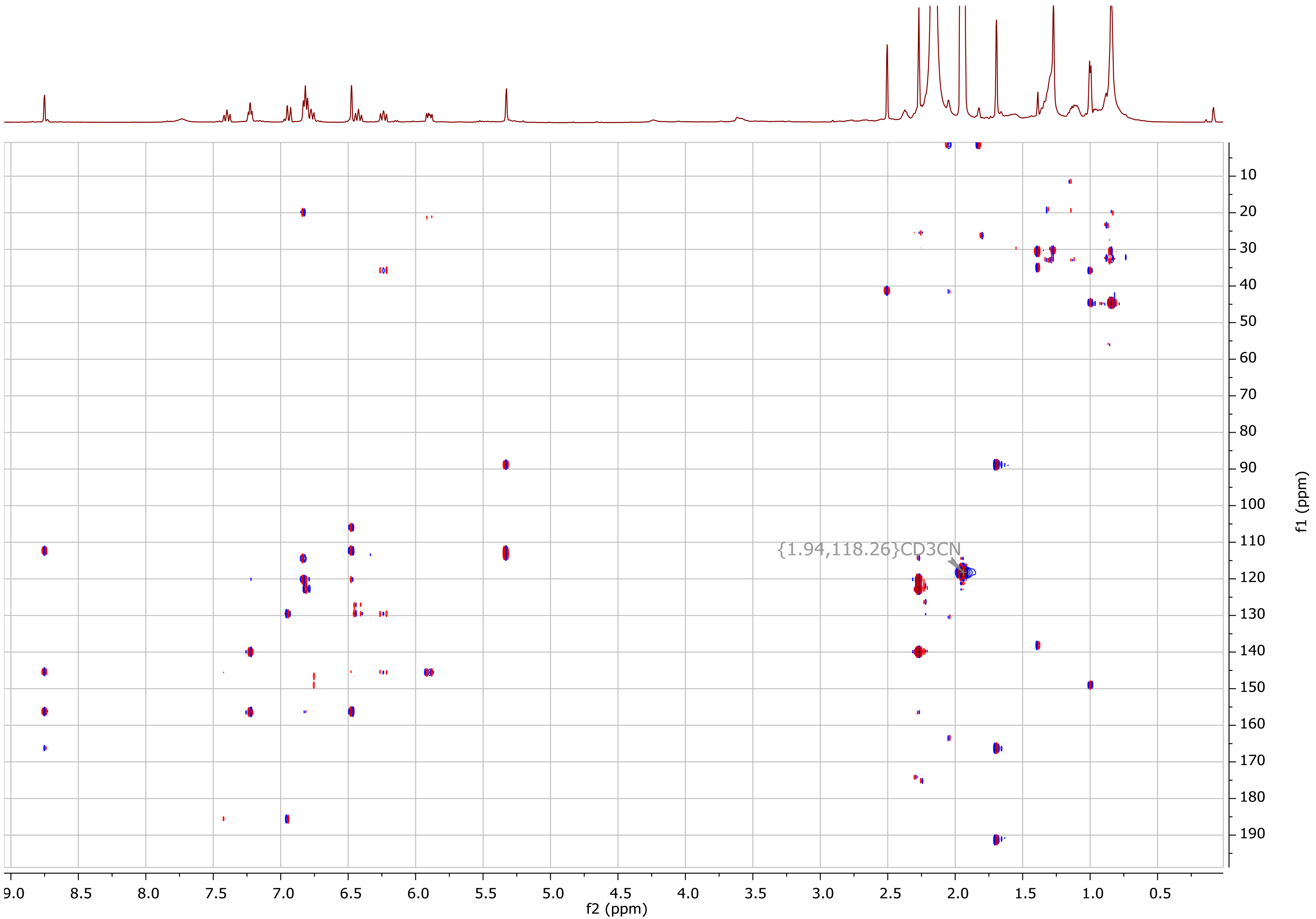

Fig. S21. HMBC NMR spectrum (600 MHz, CD_3CN) of bulbillosin C (**3**).

a

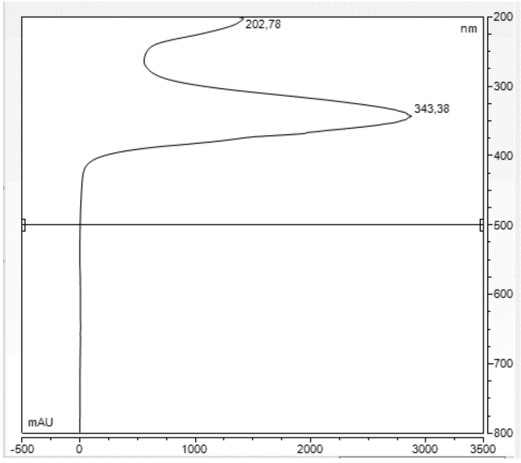

b

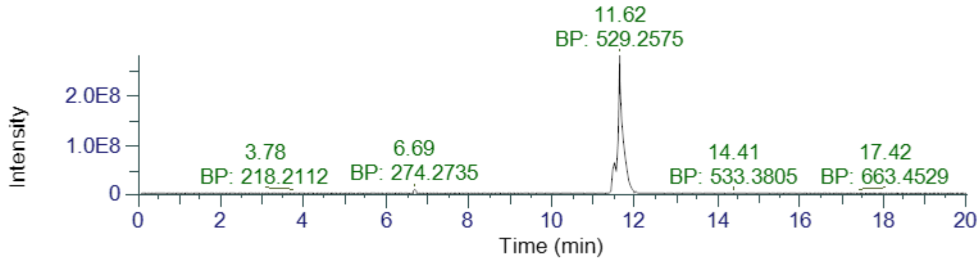

c

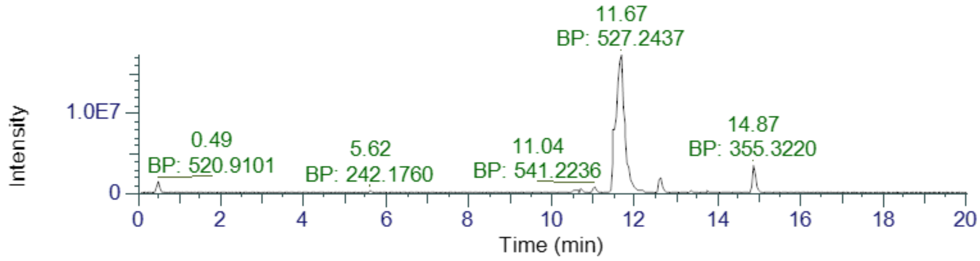

d

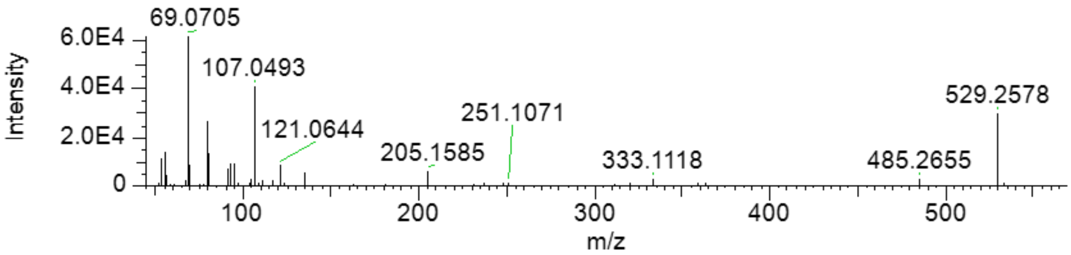

Fig. S22. HPLC-HRESI data of bulbillosin D (**4**). a. UV-vis spectrum. b. ESI Full MS in pos. ion mode (BPC). c. ESI Full MS in neg. ion mode (BPC). d. MS/MS in pos. ion mode.

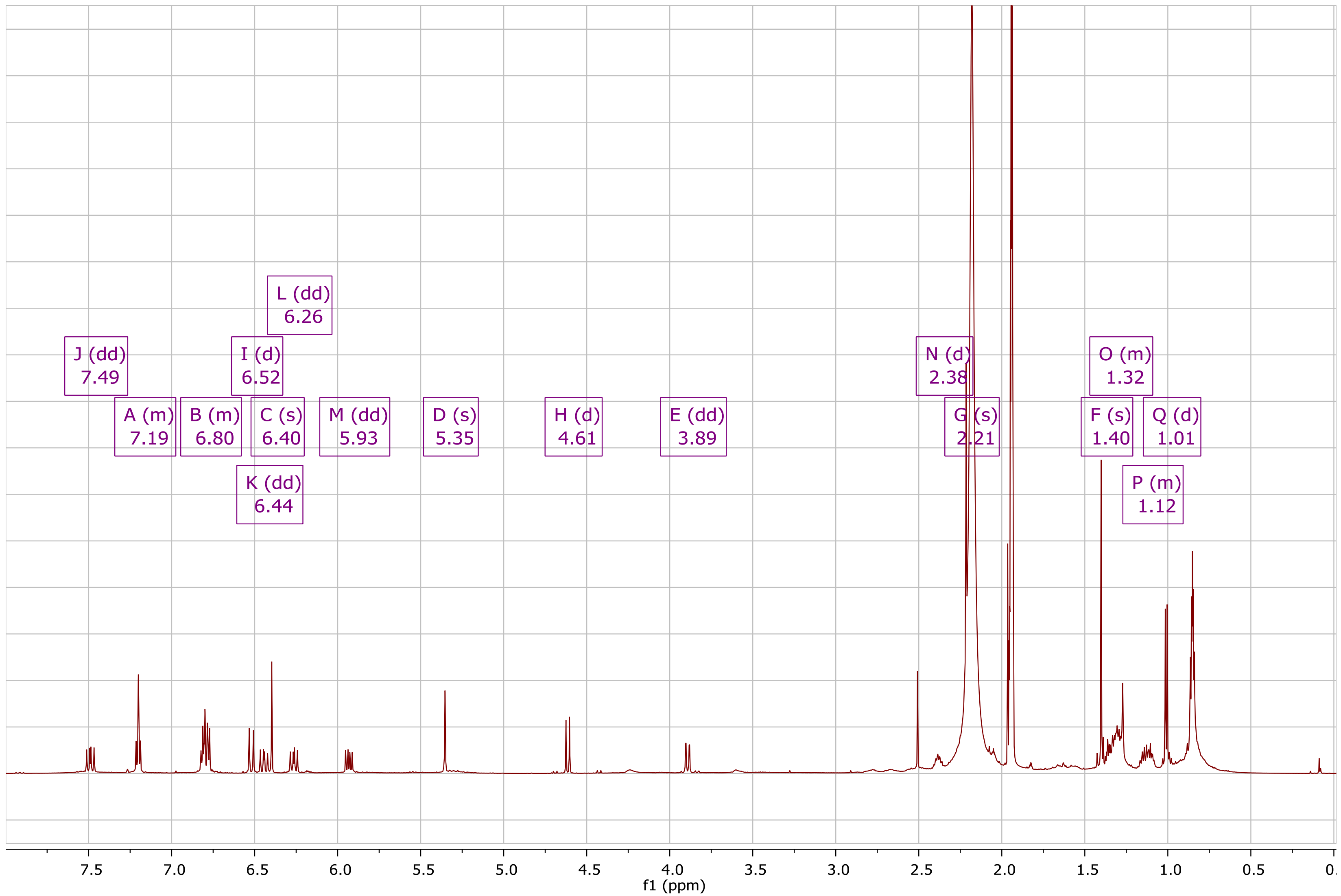

Fig. S23. Proton NMR spectrum (600 MHz, CD_3CN) of bulbillosin D (**4**).

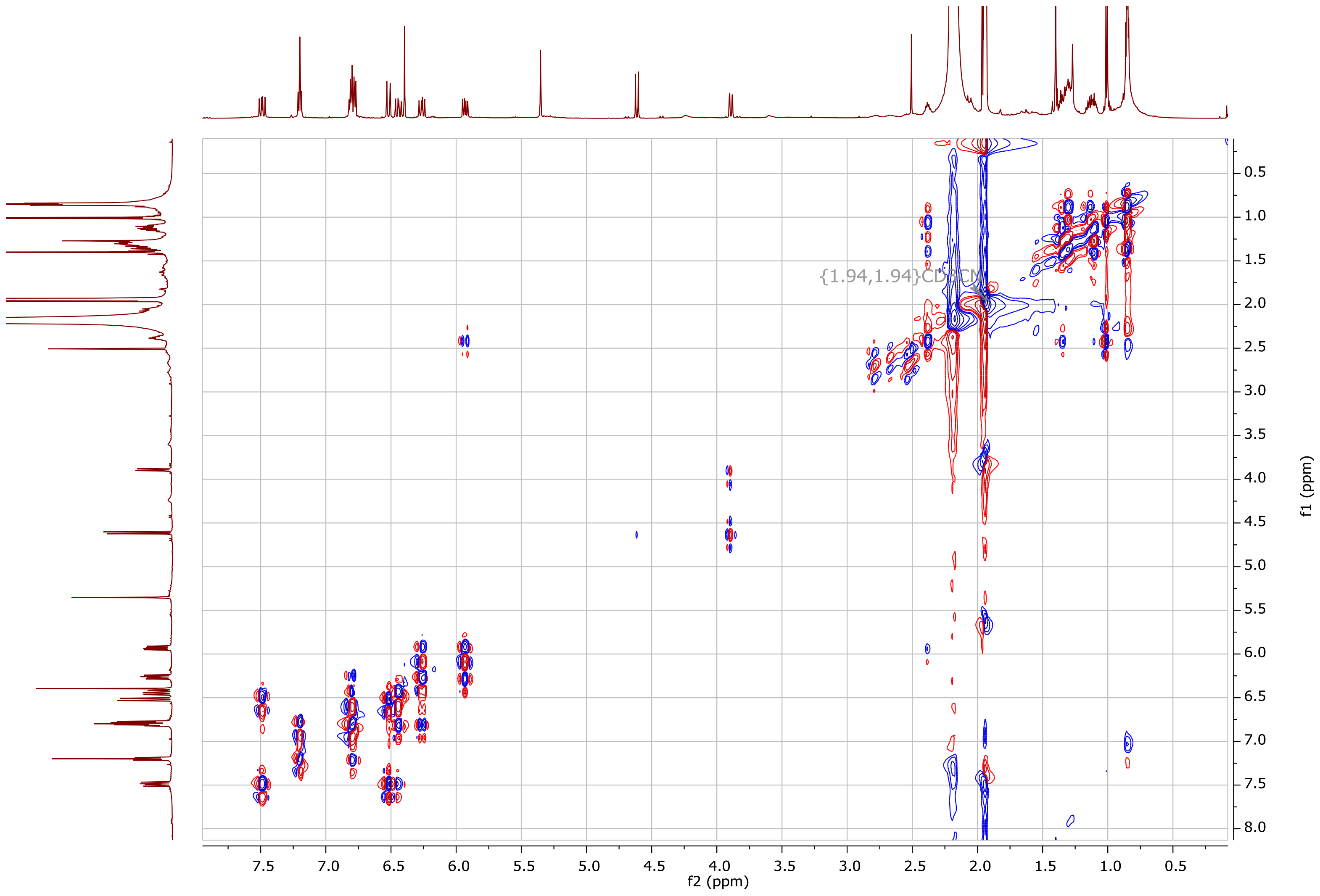

Fig. S24. COSY NMR spectrum (600 MHz, CD_3CN) of bulbillosin D (**4**).

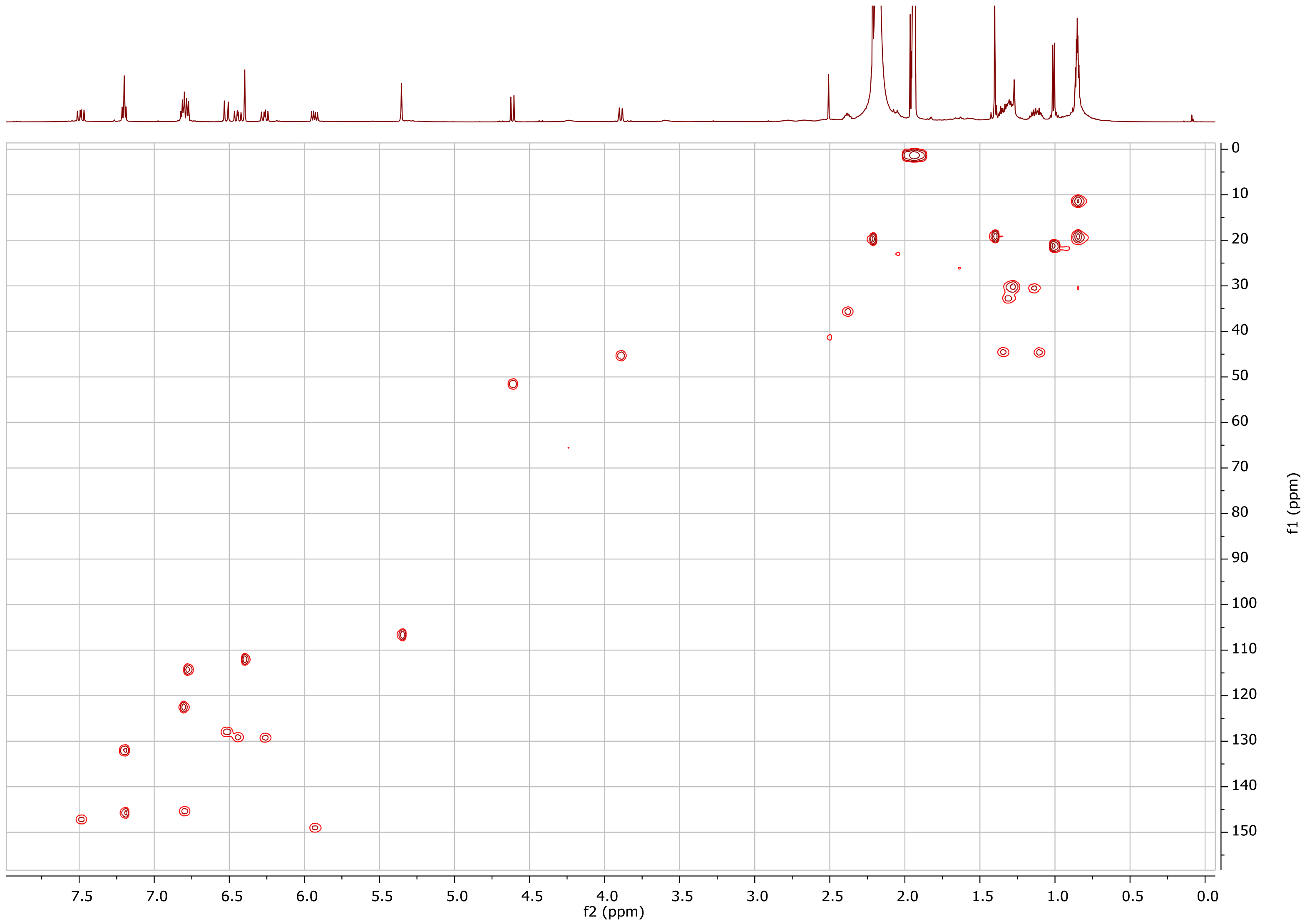

Fig. S25. HSQC NMR spectrum (600 MHz, CD_3CN) of bulbillosin D (**4**).

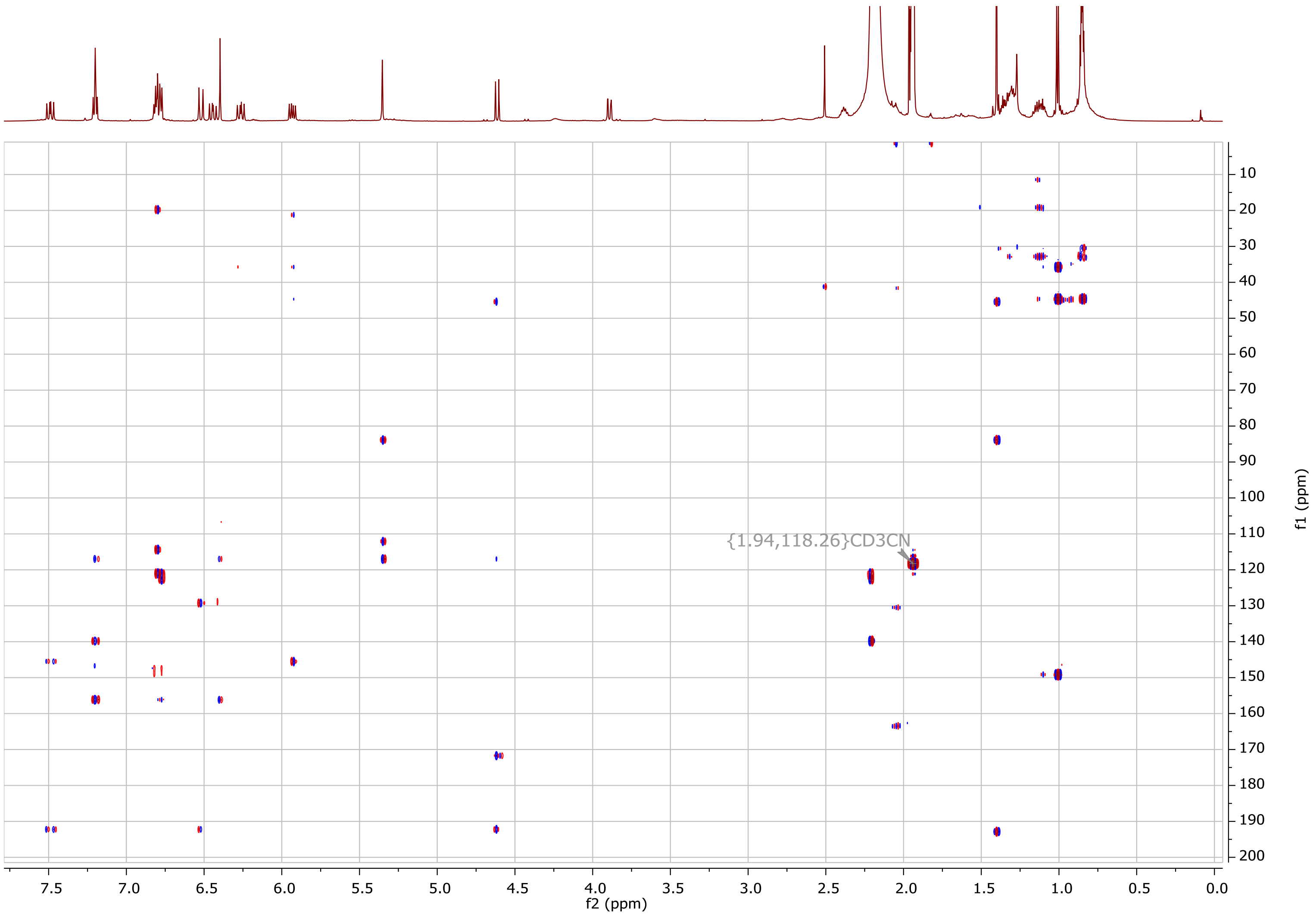

Fig. S26. HMBC NMR spectrum (600 MHz, CD_3CN) of bulbillosin D (**4**).

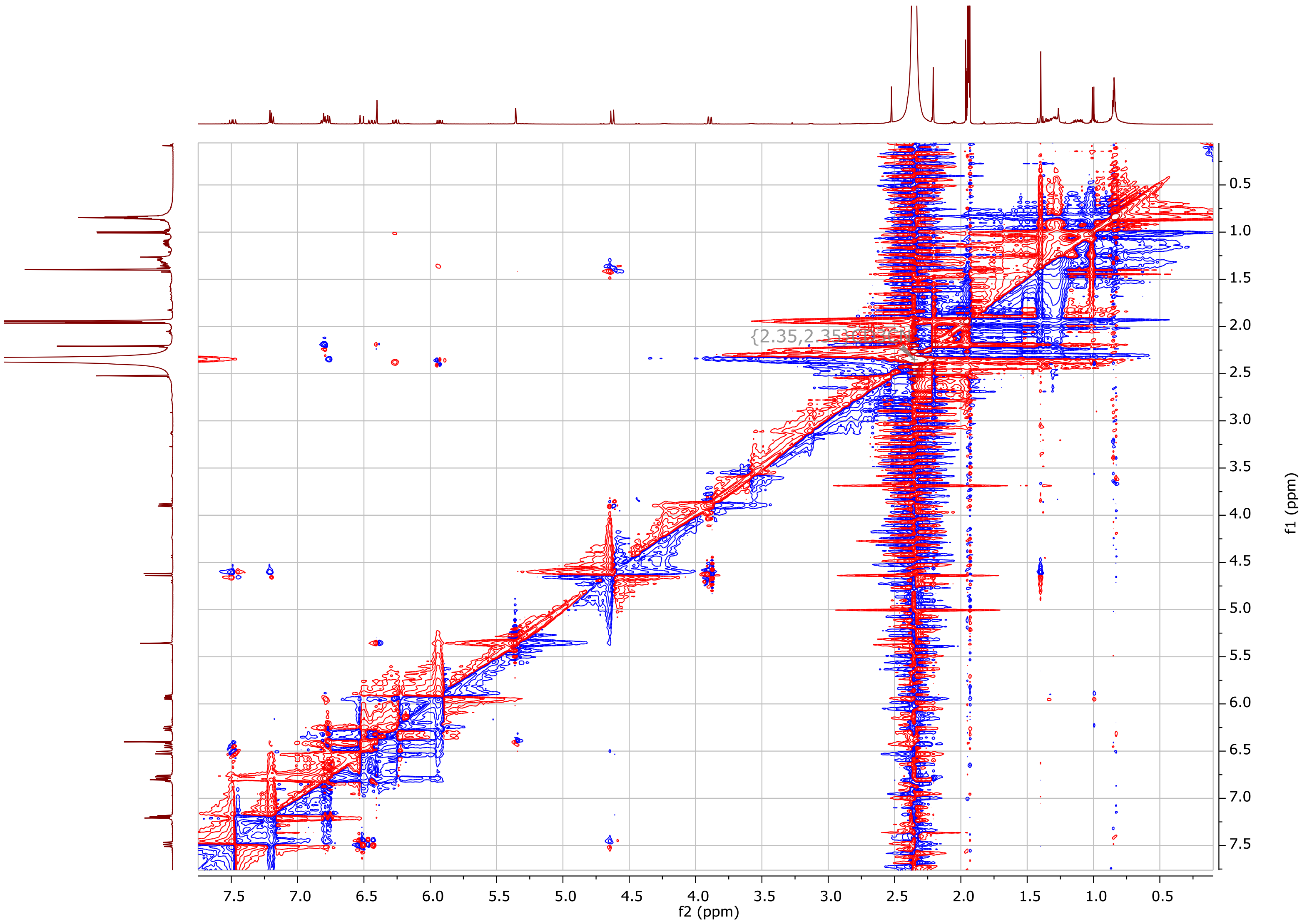

Fig. S27. NOESY NMR spectrum (600 MHz, CD_3CN) of bulbillosin D (**4**).

a

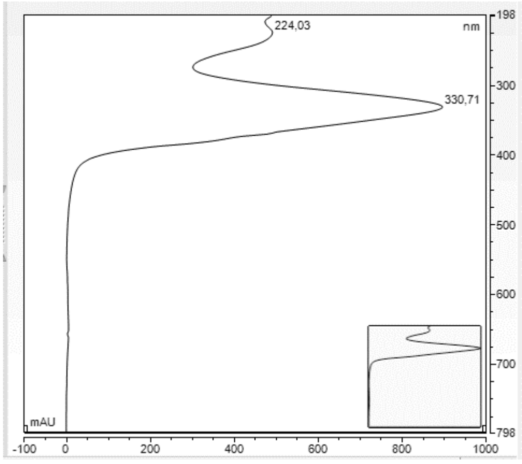

b

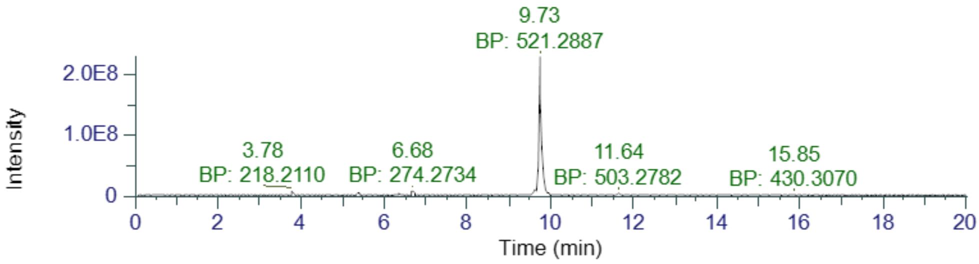

c

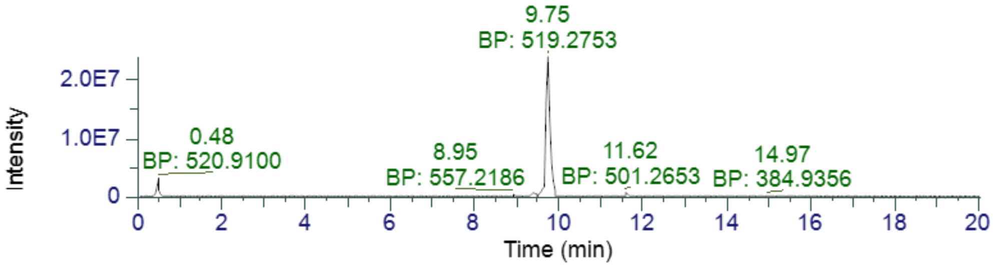

d

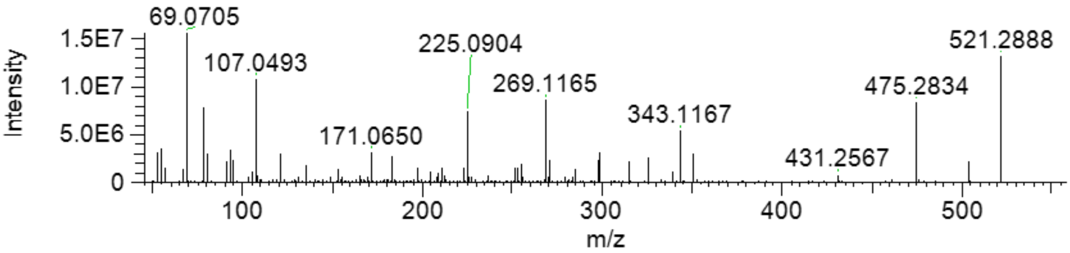

Fig. S28. HPLC-HRESI data of bulbillosin E (**5**). a. UV-vis spectrum. b. ESI Full MS in pos. ion mode (BPC). c. ESI Full MS in neg. ion mode (BPC). d. MS/MS in pos. ion mode.

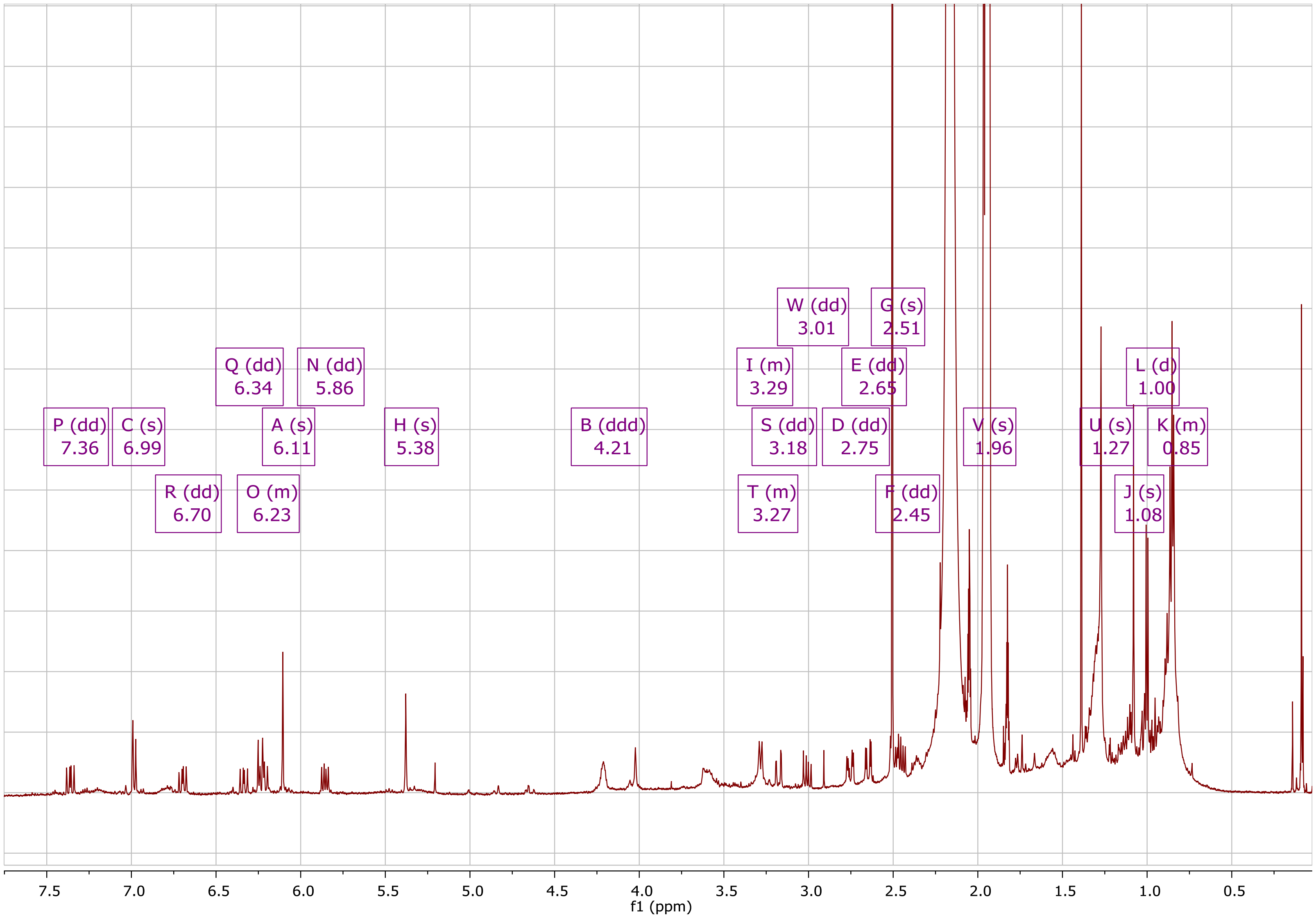

Fig. S29. Proton NMR spectrum (600 MHz, CD_3CN) of bulbillosin E (**5**).

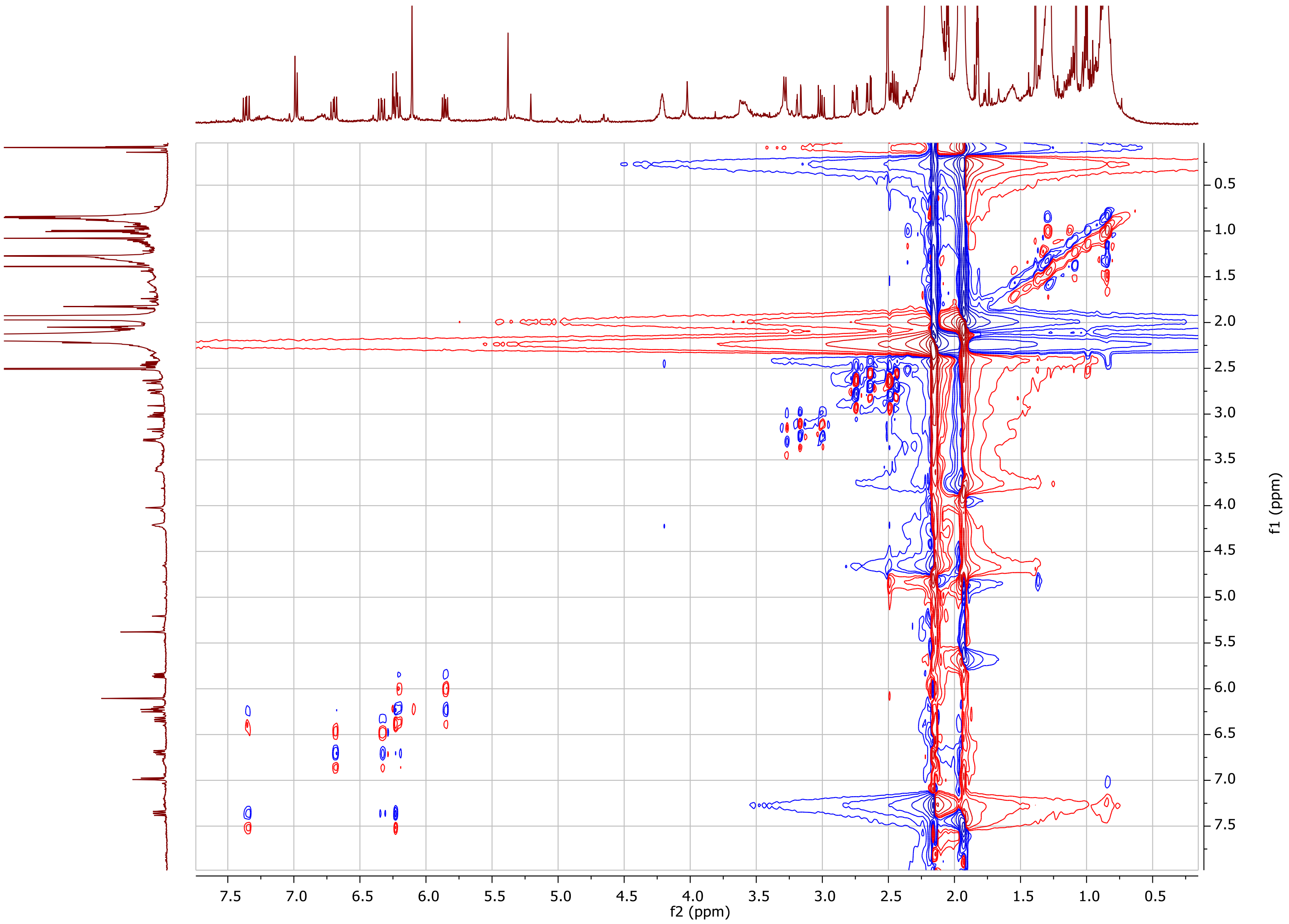

Fig. S30. COSY NMR spectrum (600 MHz, CD_3CN) of bulbillosin E (**5**).

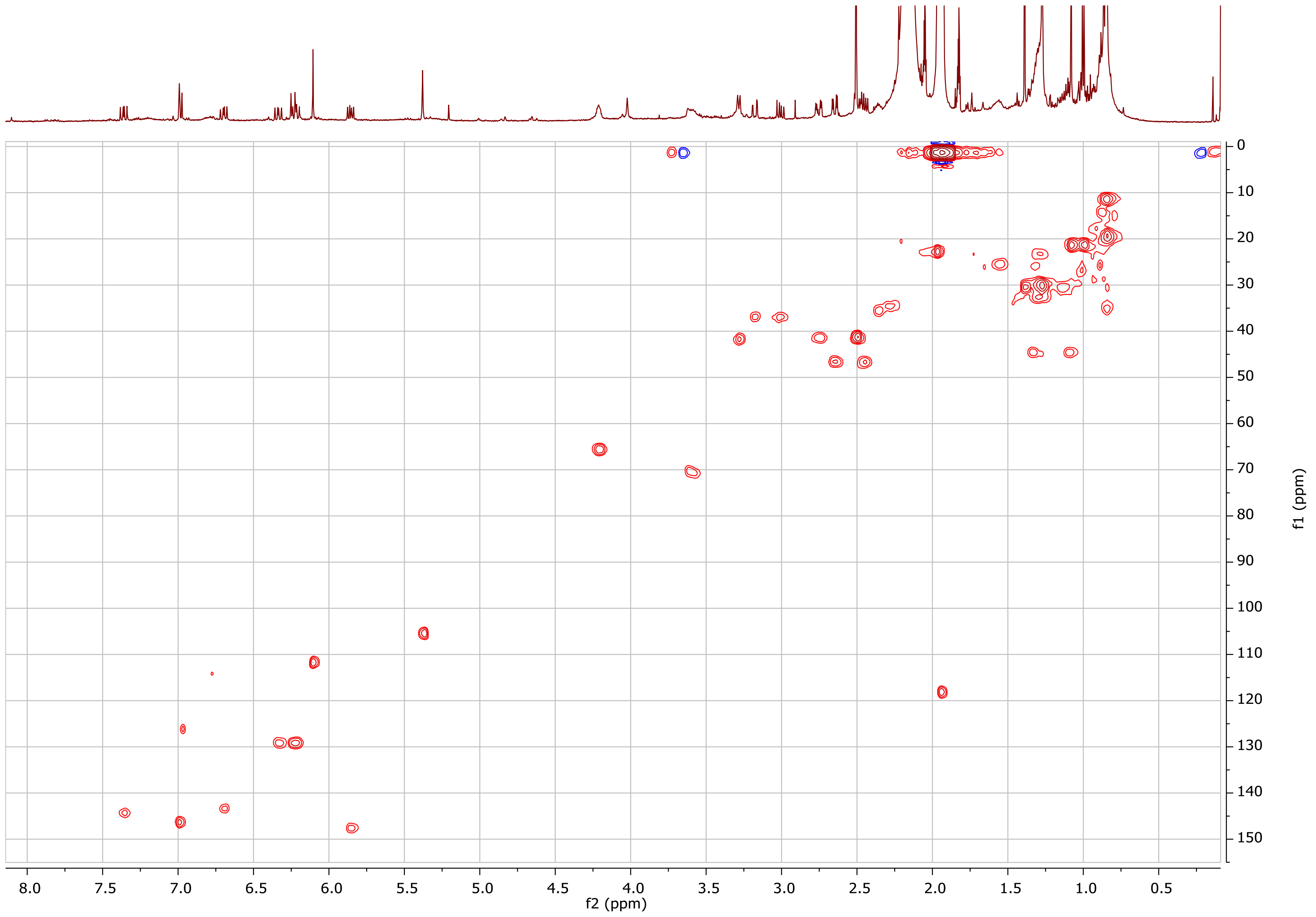

Fig. S31. HSQC NMR spectrum (600 MHz, CD_3CN) of bulbillosin E (**5**).

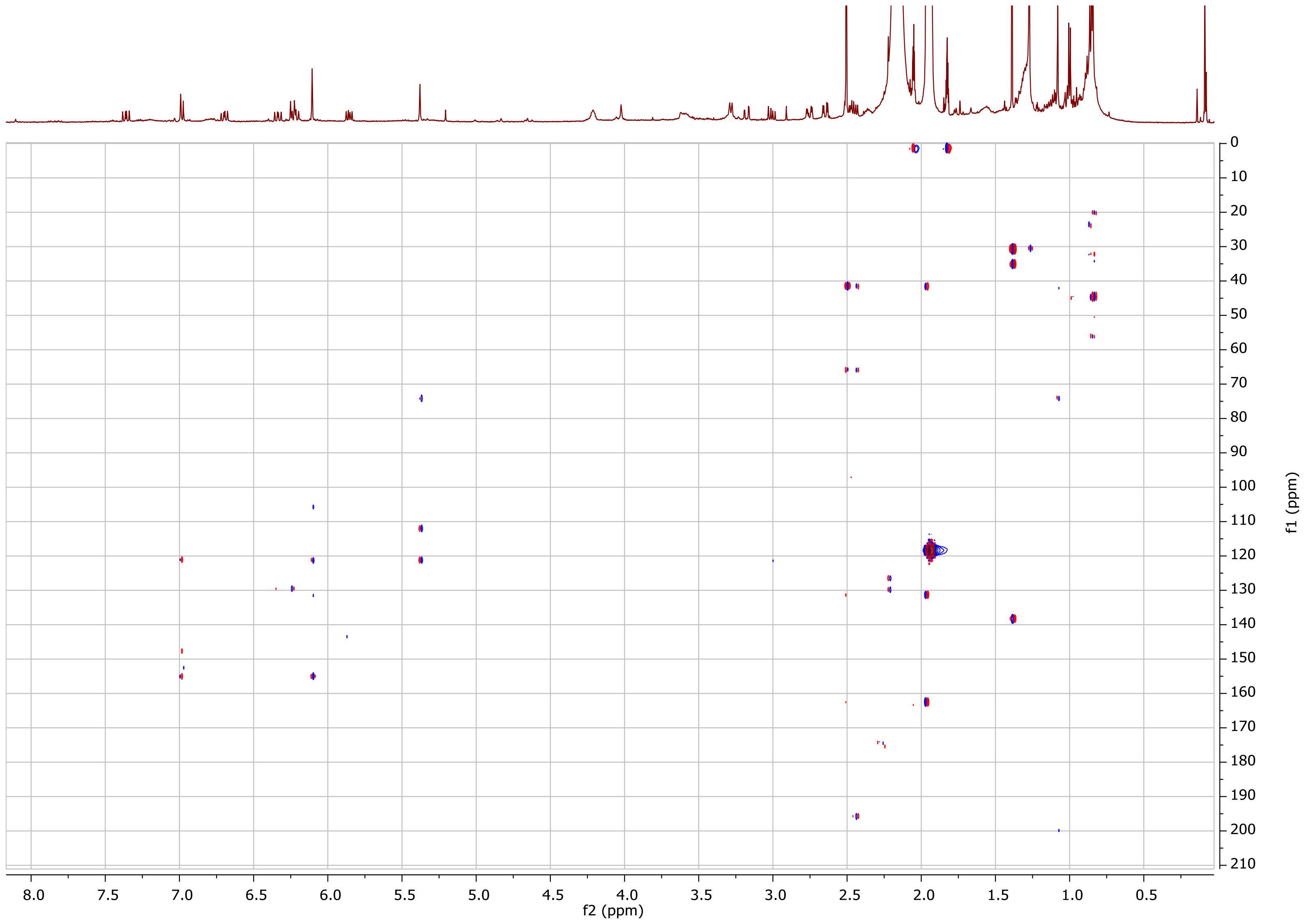

Fig. S32. HMBC NMR spectrum (600 MHz, CD_3CN) of bulbillosin E (**5**).

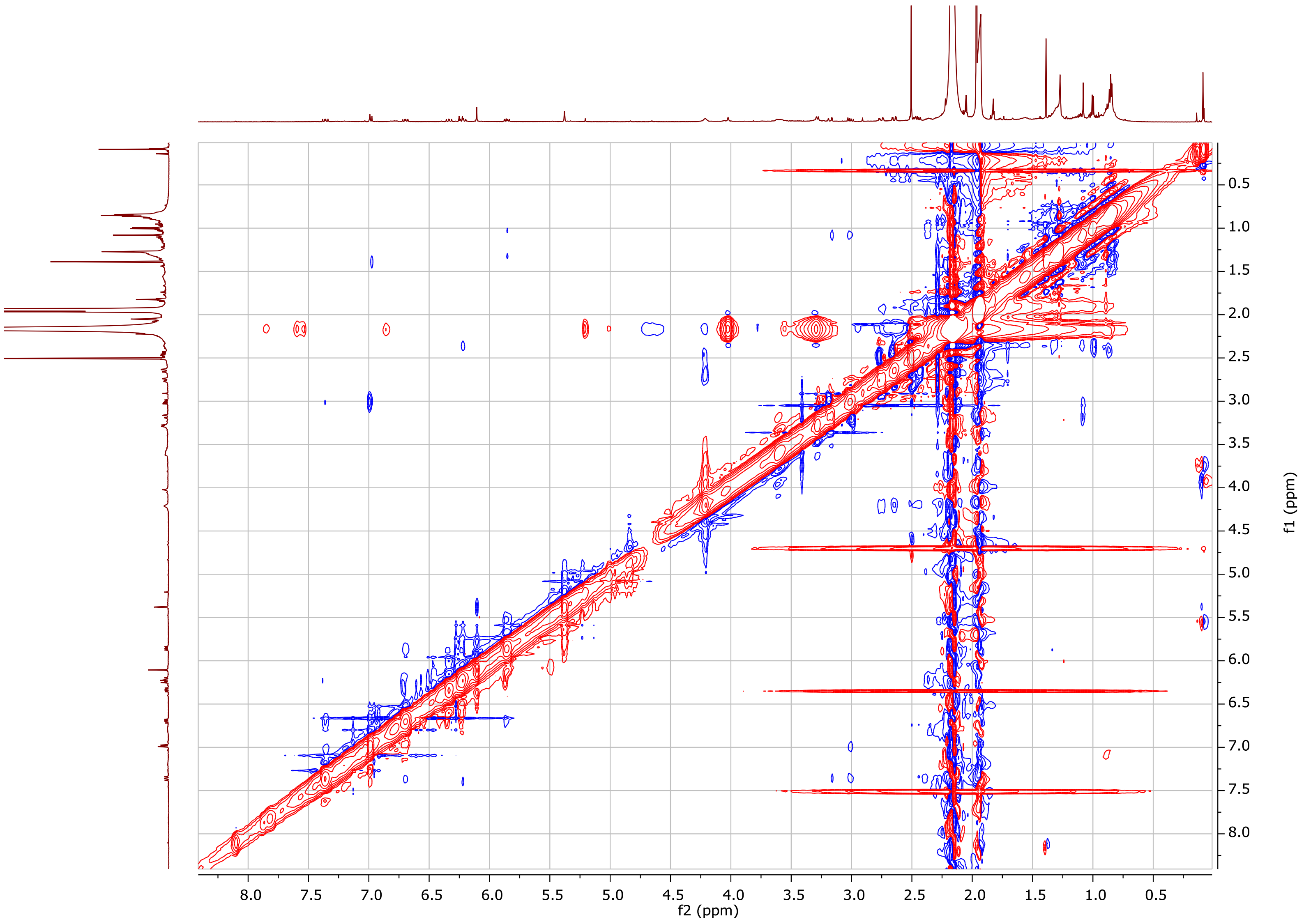

Fig. S33. NOESY NMR spectrum (600 MHz, CD_3CN) of bulbillosin E (**5**).

Spectroscopic and spectrometric data of the bulbillosins

*Bulbillosin A (**1**)*

Yellow solid; CD (MeOH) λ_{max} ($\delta\varepsilon$):227 nm (+4.9), 290 nm (+4.8), 359 nm (+8.7); UV (from HPLC-DAD, undefined solvent) λ_{max} 226 nm, 342 nm; ^{1}H NMR data, see **Table 1**; ^{13}C NMR data, see **Table 2**; HRESIMS *m/z* 547.2687 $[M+H]^+$ (calcd. for $C_{33}H_{39}O_7$, 547.2690, Δ 0.5 ppm) and 545.2547 $[M-H]^-$; R_t = 11.6 min.

*Bulbillosin B (**2**)*

Yellow solid; CD (MeOH) λ_{max} ($\delta\varepsilon$):209 nm (+5.1), 239 nm (+5.3), 275nm (-2.6), 311 nm (+7.0), 362 mn (+19.2), 434 nm (-6.3); UV (from HPLC-DAD, undefined solvent) λ_{max} 231 nm, 284 nm, 356 nm; ^{1}H NMR data, see **Table 1**; ^{13}C NMR data, see **Table 2**; HRESIMS *m/z* 545.2529 $[M+H]^+$ (calcd. for $C_{33}H_{37}O_7$, 545.2534, Δ 0.9 ppm) and 543.2398 $[M-H]^-$; R_t = 10.31 min.

*Bulbillosin C (**3**)*

Yellow solid; CD (MeOH) λ_{max} ($\delta\varepsilon$):215 nm (+3.7), 311 nm (+4.1), 371 nm (+9.9), 443 nm (-0.2); UV (from HPLC-DAD, undefined solvent) λ_{max} 291 nm, 355 nm; ^{1}H NMR data, see **Table 1**; ^{13}C NMR data, see **Table 2**; HRESIMS *m/z* 527.2422 $[M+H]^+$ (calcd. for $C_{33}H_{35}O_6$, 527.2428, Δ 1.1 ppm) and 525.2283 $[M-H]^-$; R_t = 12.1 min.

*Bulbillosin D (**4**)*

Yellow solid; CD (MeOH) λ_{max} ($\delta\varepsilon$):206 nm (+4.0), 323 nm (+9.7), 362 nm (+12.6); UV (from HPLC-DAD, undefined solvent) λ_{max} 203 nm, 343 nm; ^{1}H NMR data, see **Table 1**; ^{13}C NMR data, see **Table 2**; HRESIMS *m/z* 529.2575 $[M+H]^+$ (calcd. for $C_{33}H_{37}O_6$, 529.2585, Δ 1.9 ppm) and 527.2437 $[M-H]^-$; R_t = 11.6 min.

*Bulbillosin E (**5**)*

Yellow solid; CD (MeOH) λ_{max} ($\delta\varepsilon$):359 nm (+3.6); UV (from HPLC-DAD, undefined solvent) λ_{max} 224 nm, 331 nm; ^{1}H NMR data, see **Table 1**; ^{13}C NMR data, see **Table 2**; HRESIMS *m/z* 521.2887 $[M+H]^+$ (calcd. for $C_{32}H_{41}O_6$, 521.2898, Δ 2.1 ppm) and 519.2753 $[M-H]^-$; R_t = 9.7 min.

Table S4 Comparison of experimental ^{13}C NMR shifts of the side chain of bulbillosins A-E (**1-5**) with calculated ^{13}C NMR shifts of sambutoxin analogues (**6-9**) and experimental ^{13}C NMR shifts (+)-*N*-deoxymilitarinone A (**10**).

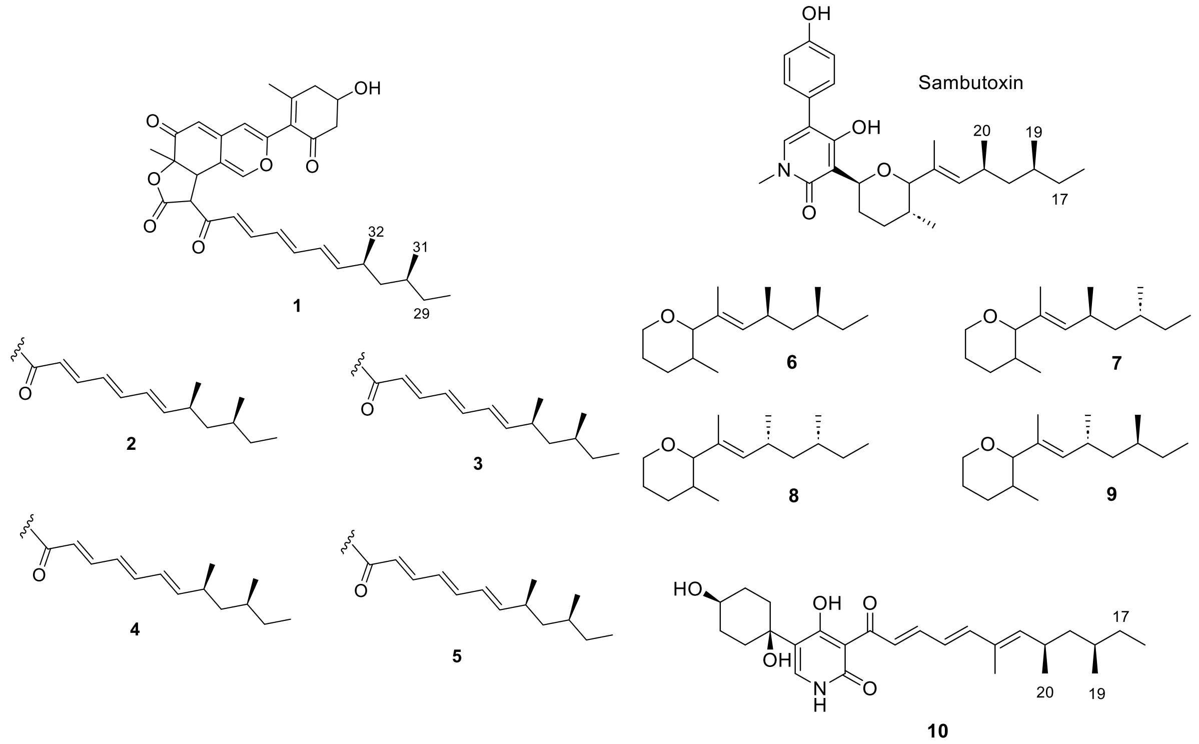

Compound	δC17	δC19	δC20	δC29	δC31	δC32	Δ C20-C19	Δ C31-C32
**1**				30.5	19.2	21.3		2.1
**2**				30.5	19.2	21.2		2.0
**3**				30.4	19.2	21.2		2.0
**4**				30.6	19.7	21.3		2.1
**5**				30.5	19.3	21.3		2.0
**6**[a]	31.3	18.4	21.3				2.9	
**7**[a]	28.3	19.4	20.9				1.5	
**8**[a]	31.5	18.1	21.0				2.9	
**9**[a]	27.7	19.8	20.5				0.7	
**10**[b]	31.3	19.5	21.6				2.1	

a. (Stahl et al. 1996); b. (Cheng et al. 2006)

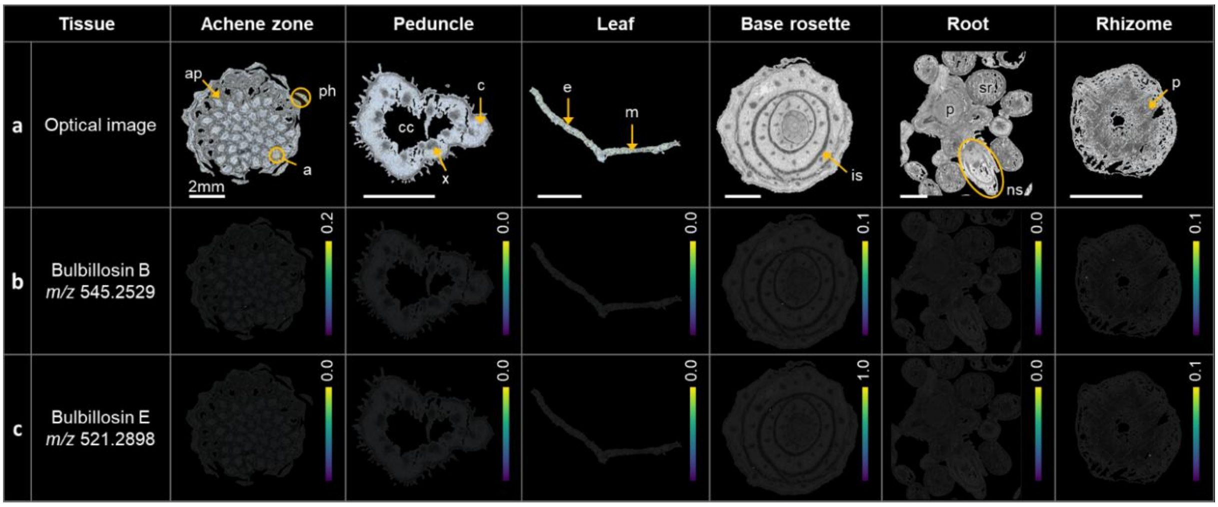

Fig. S34. Mass spectrometry imaging of *A. tataricus* tissues without detectable azaphilones. The relative distributions are displayed as heat maps, with the color code between black (for 0 ions detected) and yellow (for the maximum percentage of ions). The percentages were adjusted by compound normalized ion intensity. The resulting mass spectrometry images were all normalized by TIC

Table S5 Accurate mass measurement of azaphilones in *A. tataricus* tissues using MALDI-MSI. nd: not detected.

**Compound**	***m/z* of azaphilones $[M + H]^+$**		
	**theoretical**	**observed**	**error (ppm)**
Bulbillosin A	547.2690	547.2681	-1.6
Bulbillosin B	527.2428	527.2422	-1.1
Bulbillosin C	529.2585	529.2575	-1.9
Bulbillosin D	545.2537	nd	
Bulbillosin E	521.2898	nd	

Developmental Therapeutics Program
One Dose Bar Graph

NSC: D-844494 / 1	Conc: 1.00E-5 Molar	Test Date: May 08, 2023
Experiment ID: 2305OS25		Report Date: Oct 21, 2023

Panel/Cell Line	Growth Percent
Leukemia	
CCRF-CEM	2.77
HL-60(TB)	-20.30
K-562	4.99
MOLT-4	3.79
RPMI-8226	-23.94
SR	5.57
Non-Small Cell Lung Cancer	
A549/ATCC	21.65
EKVX	-3.83
HOP-62	-25.41
HOP-92	7.18
NCI-H226	-77.05
NCI-H23	3.90
NCI-H322M	28.08
NCI-H460	-30.57
NCI-H522	-54.94
Colon Cancer	
COLO 205	-31.69
HCC-2998	-51.64
HCT-116	2.71
HCT-15	-81.44
HT29	5.48
KM12	4.33
SW-620	-56.65
CNS Cancer	
SF-268	21.25
SF-295	6.65
SF-539	-55.07
SNB-19	3.34
SNB-75	68.34
U251	-59.06
Melanoma	
LOX IMVI	-89.98
MALME-3M	-77.54
M14	-35.47
MDA-MB-435	-61.77
SK-MEL-2	8.07
SK-MEL-28	-84.31
SK-MEL-5	-94.85
UACC-257	5.58
UACC-62	-58.07
Ovarian Cancer	
IGROV1	3.10
OVCAR-3	-90.67
OVCAR-4	13.01
OVCAR-5	-56.91
OVCAR-8	-19.66
NCI/ADR-RES	24.01
SK-OV-3	37.05
Renal Cancer	
786-0	26.04
A498	59.59
ACHN	9.97
CAKI-1	27.61
RXF 393	-32.04
SN12C	-22.80
TK-10	57.54
UO-31	0.67
Prostate Cancer	
PC-3	2.95
DU-145	24.95
Breast Cancer	
MCF7	-0.67
MDA-MB-231/ATCC	-40.07
HS 578T	-1.46
BT-549	6.92
T-47D	16.04
MDA-MB-468	-80.22

Bar Graph

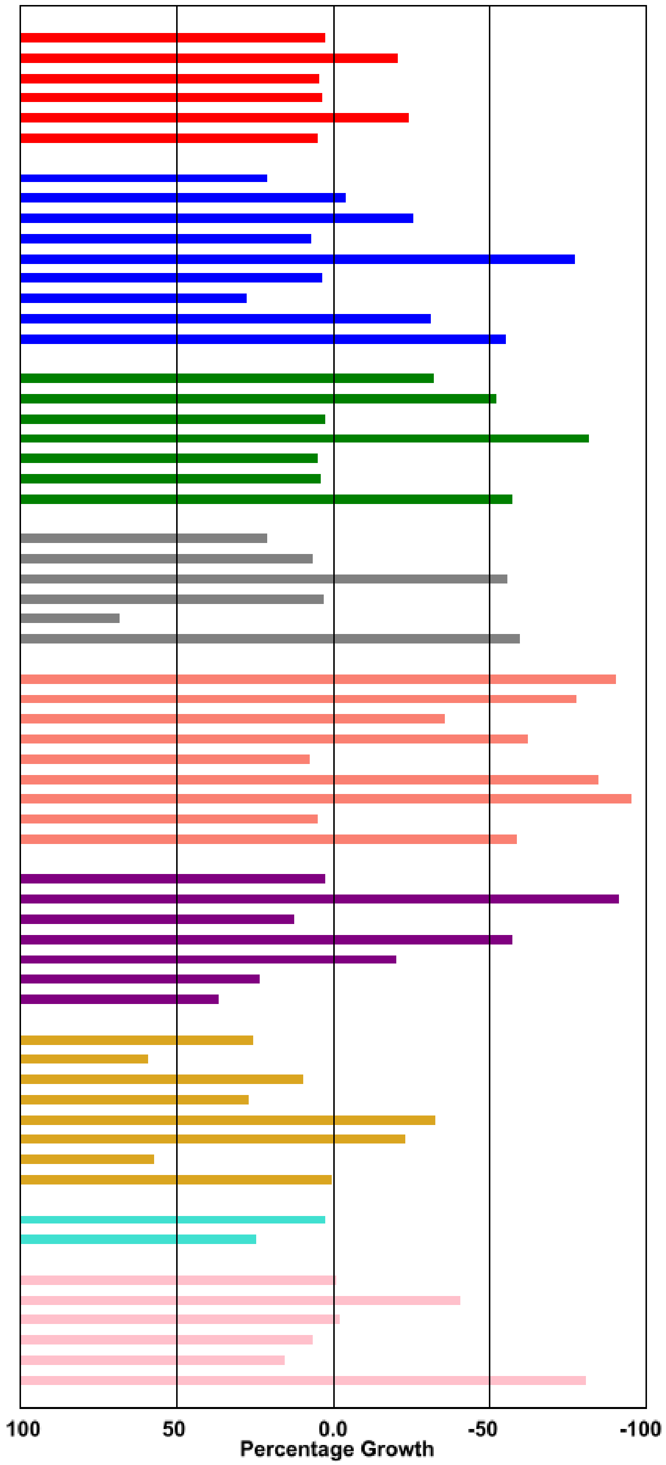

Fig. S35. NCI-60 one-dose screen (10 µM concentration), one dose bar graph for bulbillosin A (**1**).

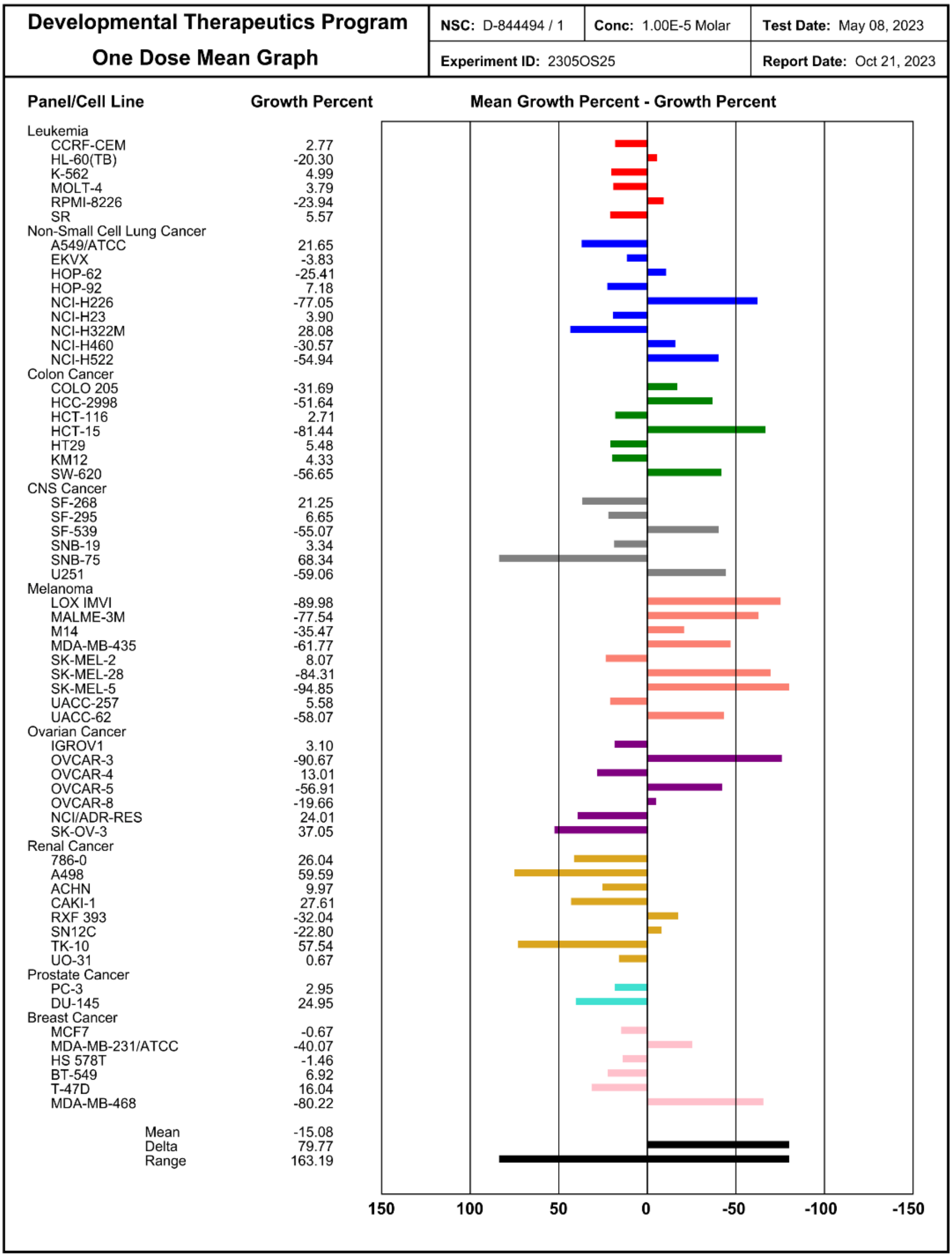

Fig. S36. NCI-60 one-dose screen (10 µM concentration), mean growth chart for bulbillosin A (**1**).

Table S6 NCI-60 five-dose screen, *in-vitro* testing results for bulbillosin A (**1**).

National Cancer Institute Developmental Therapeutics Program In-Vitro Testing Results

NSC : D - 844494 / 1	Experiment ID : 2309NS79	Test Type : 08	Units : Molar
Report Date : October 19, 2023	Test Date : September 18, 2023	QNS :	MC :
COMI : CoHaM	Stain Reagent : SRB Dual-Pass Related	SSPL : 1CNF	

			Log10 Concentration												
	Time		Mean Optical Densities					Percent Growth							
Panel/Cell Line	Zero	Ctrl	-8.1	-7.1	-6.1	-5.1	-4.1	-8.1	-7.1	-6.1	-5.1	-4.1	GI50	TGI	LC50
Leukemia															
CCRF-CEM	0.477	2.436	2.473	2.002	2.219	0.574	0.320	102	78	89	5	-33	2.18E-6	1.01E-5	> 7.50E-5
HL-60(TB)	0.628	2.539	2.227	2.334	2.056	0.631	0.276	84	89	75	0	-56	1.61E-6	7.54E-6	5.84E-5
K-562	0.208	1.792	1.760	1.780	1.603	0.543	0.216	98	99	88	21	1	2.78E-6	> 7.50E-5	> 7.50E-5
MOLT-4	0.634	2.690	2.664	2.599	2.307	0.818	0.306	99	96	81	9	-52	2.03E-6	1.05E-5	7.00E-5
RPMI-8226	0.716	2.400	2.380	2.425	2.243	0.668	0.500	99	101	91	-7	-30	1.96E-6	6.40E-6	> 7.50E-5
SR	0.232	0.991	0.994	0.989	0.915	0.231	0.122	100	100	90	0	-47	2.07E-6	7.42E-6	> 7.50E-5
Non-Small Cell Lung Cancer															
A549/ATCC	0.493	2.073	2.020	2.129	2.074	0.970	0.006	97	104	100	30	-99	3.90E-6	1.28E-5	3.14E-5
EKVX	0.744	1.734	1.723	1.794	1.695	0.861	0.107	99	106	96	12	-86	2.64E-6	9.92E-6	3.23E-5
HOP-62	0.530	2.020	2.093	2.043	2.112	1.268	0.183	105	102	106	50	-66	7.36E-6	2.02E-5	5.49E-5
HOP-92	1.317	1.790	1.735	1.756	1.713	1.484	0.360	88	93	84	35	-73	3.72E-6	1.59E-5	4.62E-5
NCI-H226	1.000	1.906	1.847	1.858	1.876	1.401	0.262	94	95	97	44	-74	5.82E-6	1.78E-5	4.71E-5
NCI-H23	0.650	2.030	1.978	2.082	1.958	1.122	0.124	96	104	95	34	-81	4.11E-6	1.49E-5	4.04E-5
NCI-H322M	0.745	2.094	2.038	1.963	1.916	1.320	-0.056	96	90	87	43	-100	5.10E-6	1.49E-5	3.35E-5
NCI-H460	0.264	1.674	1.643	1.674	1.550	0.535	0.028	98	100	91	19	-89	2.80E-6	1.13E-5	3.25E-5
NCI-H522	1.366	2.957	2.872	2.864	2.787	1.583	0.183	95	94	89	14	-87	2.48E-6	1.03E-5	3.23E-5
Colon Cancer															
COLO 205	0.479	2.339	2.431	2.393	2.305	0.900	0.123	105	103	98	23	-74	3.26E-6	1.28E-5	4.21E-5
HCC-2998	0.655	2.392	2.092	2.316	2.349	1.129	-0.020	83	96	98	27	-100	3.56E-6	1.23E-5	3.03E-5
HCT-116	0.885	3.402	3.404	3.410	3.403	2.454	0.162	100	100	100	62	-82	9.13E-6	2.03E-5	4.52E-5
HCT-15	0.320	2.015	1.960	1.963	1.846	0.194	0.011	97	97	90	-39	-97	1.53E-6	3.72E-6	1.15E-5
HT29	0.289	1.348	1.404	1.424	1.287	0.283	0.043	105	107	94	-2	-85	2.16E-6	7.14E-6	2.83E-5
KM12	0.543	1.942	1.779	1.889	1.714	0.810	-0.029	88	96	84	19	-100	2.49E-6	1.08E-5	2.85E-5
SW-620	0.318	1.944	1.772	1.821	1.718	0.393	0.061	89	92	86	5	-81	2.08E-6	8.48E-6	3.26E-5
CNS Cancer															
SF-268	0.708	1.815	1.734	1.719	1.709	0.802	0.007	93	91	90	8	-99	2.34E-6	8.99E-6	2.62E-5
SF-295	0.776	2.260	2.128	2.204	2.165	1.218	0.150	91	96	94	30	-81	3.62E-6	1.40E-5	3.95E-5
SF-539	0.541	1.824	1.761	1.787	1.798	0.659	0.049	95	97	98	9	-91	2.60E-6	9.26E-6	2.92E-5
SNB-19	0.532	1.580	1.522	1.517	1.538	0.994	-0.018	94	94	96	44	-100	5.76E-6	1.52E-5	3.37E-5
SNB-75	1.114	1.783	1.778	1.800	1.697	1.255	0.499	99	103	87	21	-55	2.73E-6	1.42E-5	6.41E-5
U251	0.433	1.709	1.632	1.652	1.598	0.666	-0.003	94	96	91	18	-100	2.76E-6	1.07E-5	2.83E-5
Melanoma															
LOX IMVI	0.448	2.390	2.282	2.374	2.214	1.136	0.071	94	99	91	35	-84	4.09E-6	1.48E-5	3.88E-5
MALME-3M	0.462	1.837	1.785	1.794	1.695	1.276	0.027	96	97	90	59	-94	8.61E-6	1.82E-5	3.86E-5
M14	0.503	1.739	1.719	1.696	1.649	1.155	0.043	98	96	93	53	-91	7.84E-6	1.74E-5	3.87E-5
MDA-MB-435	0.740	2.490	2.300	2.526	2.390	1.172	0.272	89	102	94	25	-63	3.24E-6	1.43E-5	5.29E-5
SK-MEL-2	1.466	2.635	2.637	2.679	2.539	2.161	0.109	100	104	92	59	-93	8.65E-6	1.85E-5	3.93E-5
SK-MEL-28	0.654	1.755	1.752	1.830	1.685	0.644	-0.020	100	107	94	-2	-100	2.16E-6	7.23E-6	2.33E-5
SK-MEL-5	0.906	3.123	2.934	3.043	2.680	1.080	-0.023	91	96	80	8	-100	1.95E-6	8.86E-6	2.58E-5
UACC-257	1.220	2.483	2.441	2.528	2.445	1.678	0.086	97	104	97	36	-93	4.45E-6	1.43E-5	3.49E-5
UACC-62	0.777	2.454	2.266	2.272	2.202	1.293	-0.003	89	89	85	31	-100	3.31E-6	1.29E-5	3.11E-5
Ovarian Cancer															
IGROV1	0.661	1.199	1.145	1.142	1.132	0.874	0.057	90	89	88	40	-91	4.54E-6	1.50E-5	3.62E-5
OVCAR-3	0.614	1.500	1.439	1.537	1.459	0.270	-0.070	93	104	95	-56	-100	1.49E-6	3.20E-6	6.84E-6
OVCAR-4	0.734	1.989	1.917	2.009	1.889	1.104	0.250	94	102	92	29	-66	3.52E-6	1.53E-5	5.11E-5
OVCAR-5	0.532	1.306	1.293	1.319	1.300	0.745	0.005	98	102	99	28	-99	3.65E-6	1.24E-5	3.07E-5
OVCAR-8	0.714	2.543	2.596	2.526	2.503	1.376	0.197	103	99	98	36	-72	4.48E-6	1.61E-5	4.66E-5
NCI/ADR-RES	0.491	1.585	1.552	1.629	1.496	1.070	0.168	97	104	92	53	-66	7.93E-6	2.09E-5	5.52E-5
SK-OV-3	0.536	1.590	1.557	1.549	1.664	1.357	0.088	97	96	107	78	-84	1.12E-5	2.28E-5	4.64E-5
Renal Cancer															
786-0	0.517	2.425	2.351	2.419	2.367	1.238	0.092	96	100	97	38	-82	4.66E-6	1.55E-5	4.04E-5
A498	1.429	2.215	2.161	2.146	2.206	2.256	-0.054	93	91	99	105	-100	1.39E-5	2.44E-5	4.28E-5
ACHN	0.387	1.605	1.573	1.653	1.606	0.939	-0.044	97	104	100	45	-100	6.17E-6	1.54E-5	3.40E-5
CAKI-1	0.769	2.577	2.448	2.545	2.461	1.535	0.051	93	98	94	42	-93	5.32E-6	1.54E-5	3.59E-5
RXF 393	0.748	1.412	1.363	1.394	1.364	0.858	0.080	93	97	93	16	-89	2.72E-6	1.07E-5	3.19E-5
SN12C	0.504	1.673	1.601	1.610	1.571	0.763	0.073	94	95	91	22	-86	2.97E-6	1.20E-5	3.51E-5
TK-10	0.937	1.812	1.708	1.727	1.702	1.430	0.021	88	90	87	56	-98	8.24E-6	1.74E-5	3.67E-5
UO-31	0.621	1.939	1.731	1.799	1.746	0.921	-0.029	84	89	85	23	-100	2.75E-6	1.15E-5	2.94E-5
Prostate Cancer															
PC-3	0.604	1.920	1.860	1.913	1.749	0.792	0.135	95	99	87	14	-78	2.42E-6	1.07E-5	3.75E-5
DU-145	0.296	1.012	1.001	1.048	1.023	0.656	-0.052	98	105	102	50	-100	7.53E-6	1.62E-5	3.49E-5
Breast Cancer															
MCF7	0.702	2.539	2.317	2.350	2.326	0.843	0.119	88	90	88	8	-83	2.24E-6	9.11E-6	3.24E-5
MDA-MB-231/ATCC	0.606	1.248	1.241	1.242	1.182	0.836	0.124	99	99	90	36	-80	4.10E-6	1.53E-5	4.16E-5
HS 578T	1.002	2.309	2.102	2.121	2.119	1.170	0.818	84	86	85	13	-18	2.31E-6	1.94E-5	> 7.50E-5
BT-549	0.501	1.407	1.396	1.389	1.402	0.652	0.160	99	98	99	17	-68	2.97E-6	1.18E-5	4.59E-5
T-47D	0.748	2.026	1.874	1.895	1.802	0.987	0.271	88	90	82	19	-64	2.42E-6	1.26E-5	5.10E-5
MDA-MB-468	0.889	1.689	1.634	1.694	1.615	1.010	0.019	93	101	91	15	-98	2.59E-6	1.02E-5	2.83E-5

National Cancer Institute Developmental Therapeutics Program<br>Waterfall Graph GI50	NSC : D - 844494/1	Units :Molar	SSPL :1CNF	EXP. ID :2309NS79
	Report Date :October 19, 2023		Test Date :September 18, 2023	

Panel	Cell Name	Hollow Fiber	GI50
Ovarian Cancer	OVCAR-3	*	-5.83
Colon Cancer	HCT-15		-5.82
Leukemia	HL-60(TB)		-5.79
Melanoma	SK-MEL-5		-5.71
Leukemia	RPMI-8226		-5.71
Leukemia	MOLT-4		-5.69
Leukemia	SR		-5.68
Colon Cancer	SW-620	*	-5.68
Melanoma	SK-MEL-28		-5.67
Colon Cancer	HT29		-5.67
Leukemia	CCRF-CEM		-5.66
Breast Cancer	MCF7		-5.65
Breast Cancer	HS 578T		-5.64
CNS Cancer	SF-268		-5.63
Breast Cancer	T-47D		-5.62
Prostate Cancer	PC-3		-5.62
Non-Small Cell Lung Cancer	NCI-H522	*	-5.61
Colon Cancer	KM12		-5.60
Breast Cancer	MDA-MB-468		-5.59
CNS Cancer	SF-539		-5.58
Non-Small Cell Lung Cancer	EKVX		-5.58
Renal Cancer	RXF 393		-5.56
CNS Cancer	SNB-75		-5.56
Renal Cancer	UO-31		-5.56
CNS Cancer	U251	*	-5.56
Leukemia	K-562		-5.56
Non-Small Cell Lung Cancer	NCI-H460		-5.55
Renal Cancer	SN12C		-5.53
Breast Cancer	BT-549		-5.53
Melanoma	MDA-MB-435	*	-5.49
Colon Cancer	COLO 205	*	-5.49
Melanoma	UACC-62	*	-5.48
Ovarian Cancer	OVCAR-4		-5.45
Colon Cancer	HCC-2998		-5.45
CNS Cancer	SF-295	*	-5.44
Ovarian Cancer	OVCAR-5	*	-5.44
Non-Small Cell Lung Cancer	HOP-92		-5.43
Non-Small Cell Lung Cancer	A549/ATCC		-5.41
Melanoma	LOX IMVI	*	-5.39
Breast Cancer	MDA-MB-231/ATCC	*	-5.39
Non-Small Cell Lung Cancer	NCI-H23	*	-5.39
Melanoma	UACC-257		-5.35
Ovarian Cancer	OVCAR-8		-5.35
Ovarian Cancer	IGROV1		-5.34
Renal Cancer	786-0		-5.33
Non-Small Cell Lung Cancer	NCI-H322M		-5.29
Renal Cancer	CAKI-1		-5.27
CNS Cancer	SNB-19		-5.24
Non-Small Cell Lung Cancer	NCI-H226		-5.23
Renal Cancer	ACHN		-5.21
Non-Small Cell Lung Cancer	HOP-62		-5.13
Prostate Cancer	DU-145		-5.12
Melanoma	M14		-5.11
Ovarian Cancer	NCI/ADR-RES		-5.10
Renal Cancer	TK-10		-5.08
Melanoma	MALME-3M		-5.07
Melanoma	SK-MEL-2		-5.06
Colon Cancer	HCT-116		-5.04
Ovarian Cancer	SK-OV-3		-4.95
Renal Cancer	A498		-4.86

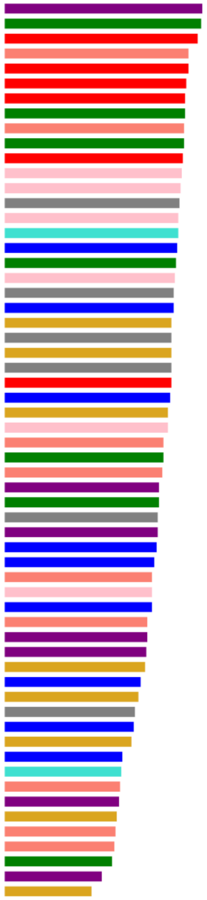

Log10 High Conc :-4.1

Fig. S37. NCI-60 five-dose screen, waterfull grapf GI_{50} for bulbillosin A (**1**).

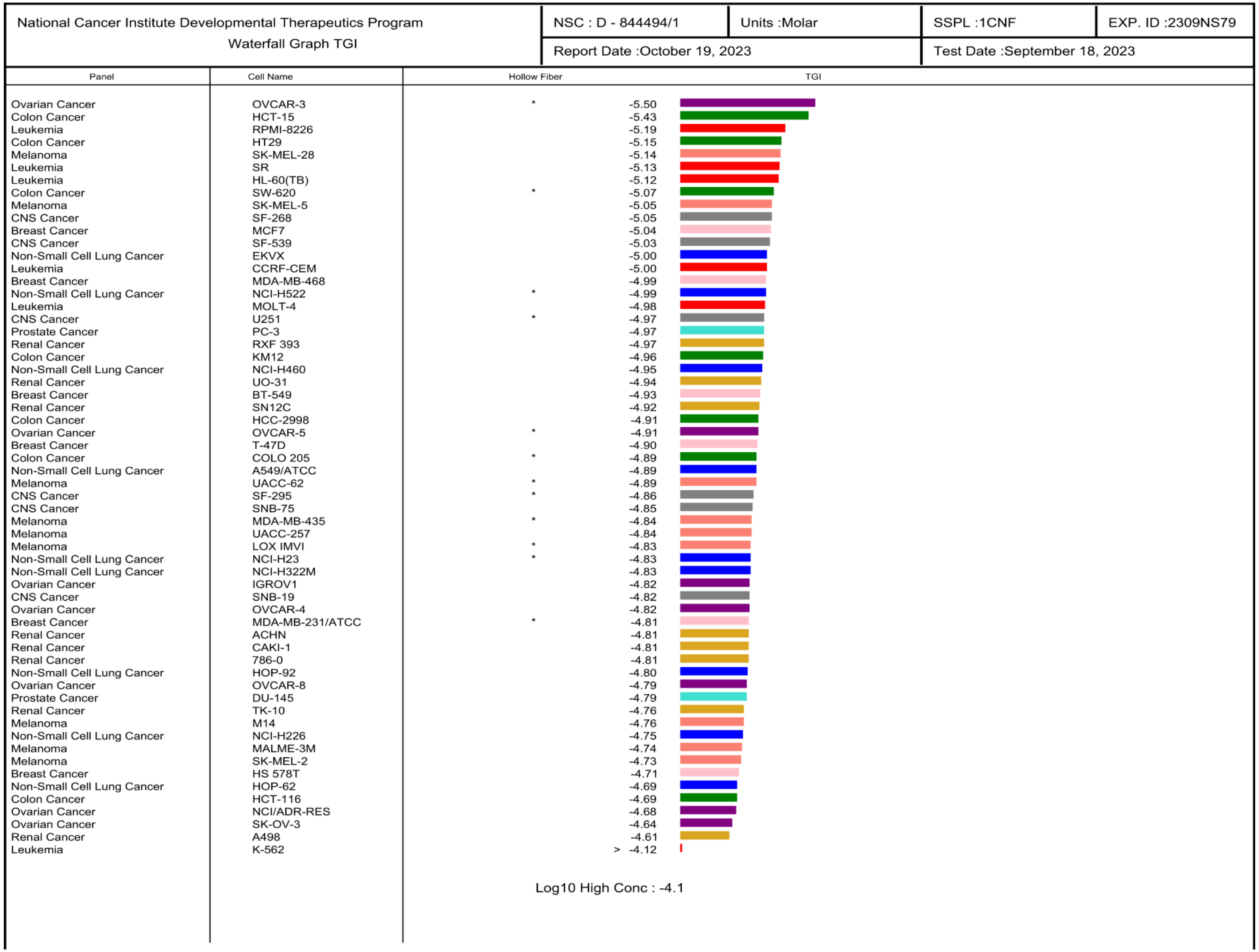

Fig. S38. NCI-60 five-dose screen, waterfull grapf TGI_{50} for bulbillosin A (**1**).

National Cancer Institute Developmental Therapeutics Program<br>Waterfall Graph LC50	NSC : D - 844494/1	Units :Molar	SSPL :1CNF	EXP. ID :2309NS79
	Report Date :October 19, 2023		Test Date :September 18, 2023	

Panel	Cell Name	Hollow Fiber	LC50
Ovarian Cancer	OVCAR-3	*	-5.16
Colon Cancer	HCT-15		-4.94
Melanoma	SK-MEL-28		-4.63
Melanoma	SK-MEL-5		-4.59
CNS Cancer	SF-268		-4.58
Breast Cancer	MDA-MB-468		-4.55
Colon Cancer	HT29		-4.55
CNS Cancer	U251	*	-4.55
Colon Cancer	KM12		-4.54
CNS Cancer	SF-539		-4.53
Renal Cancer	UO-31		-4.53
Colon Cancer	HCC-2998		-4.52
Ovarian Cancer	OVCAR-5	*	-4.51
Melanoma	UACC-62	*	-4.51
Non-Small Cell Lung Cancer	A549/ATCC		-4.50
Renal Cancer	RXF 393		-4.50
Non-Small Cell Lung Cancer	EKVX		-4.49
Non-Small Cell Lung Cancer	NCI-H522	*	-4.49
Breast Cancer	MCF7		-4.49
Non-Small Cell Lung Cancer	NCI-H460		-4.49
Colon Cancer	SW-620	*	-4.49
Non-Small Cell Lung Cancer	NCI-H322M		-4.48
CNS Cancer	SNB-19		-4.47
Renal Cancer	ACHN		-4.47
Prostate Cancer	DU-145		-4.46
Melanoma	UACC-257		-4.46
Renal Cancer	SN12C		-4.45
Renal Cancer	CAKI-1		-4.44
Ovarian Cancer	IGROV1		-4.44
Renal Cancer	TK-10		-4.43
Prostate Cancer	PC-3		-4.43
Melanoma	MALME-3M		-4.41
Melanoma	M14		-4.41
Melanoma	LOX IMVI	*	-4.41
Melanoma	SK-MEL-2		-4.41
CNS Cancer	SF-295	*	-4.40
Non-Small Cell Lung Cancer	NCI-H23	*	-4.39
Renal Cancer	786-0		-4.39
Breast Cancer	MDA-MB-231/ATCC	*	-4.38
Colon Cancer	COLO 205	*	-4.38
Renal Cancer	A498		-4.37
Colon Cancer	HCT-116		-4.35
Breast Cancer	BT-549		-4.34
Non-Small Cell Lung Cancer	HOP-92		-4.34
Ovarian Cancer	SK-OV-3		-4.33
Ovarian Cancer	OVCAR-8		-4.33
Non-Small Cell Lung Cancer	NCI-H226		-4.33
Breast Cancer	T-47D		-4.29
Ovarian Cancer	OVCAR-4		-4.29
Melanoma	MDA-MB-435	*	-4.28
Non-Small Cell Lung Cancer	HOP-62		-4.26
Ovarian Cancer	NCI/ADR-RES		-4.26
Leukemia	HL-60(TB)		-4.23
CNS Cancer	SNB-75		-4.19
Leukemia	MOLT-4		-4.15
Leukemia	CCRF-CEM		> -4.12
Leukemia	K-562		> -4.12
Leukemia	RPMI-8226		> -4.12
Leukemia	SR		> -4.12
Breast Cancer	HS 578T		> -4.12

Log10 High Conc : -4.1

Fig. S39. NCI-60 five-dose screen, waterfull grapf LC_{50} for bulbillosin A (**1**).

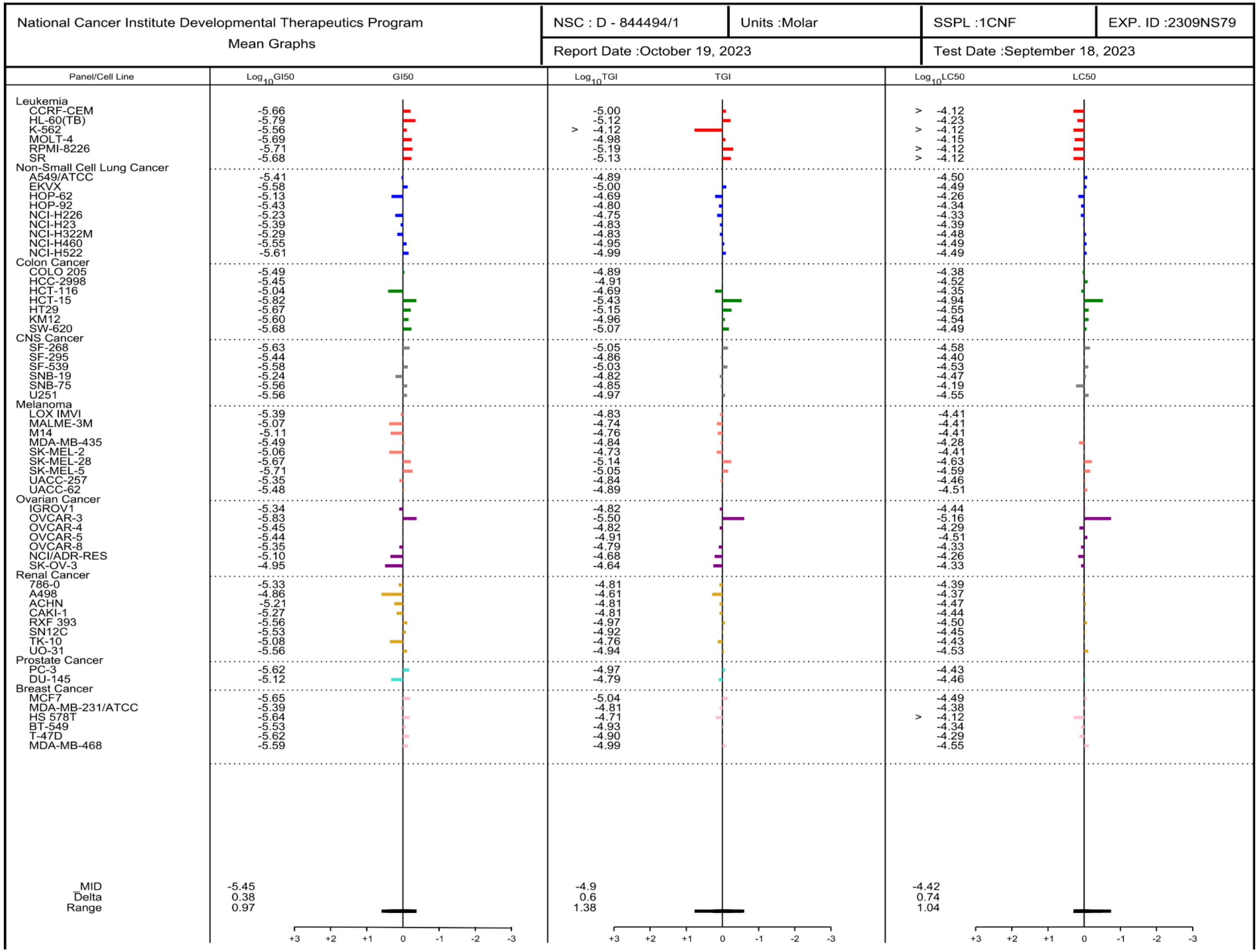

Fig. S40. NCI-60 five-dose screen, mean graphs GI_{50}, TGI_{50} and LC_{50} for bulbillosin A (**1**).

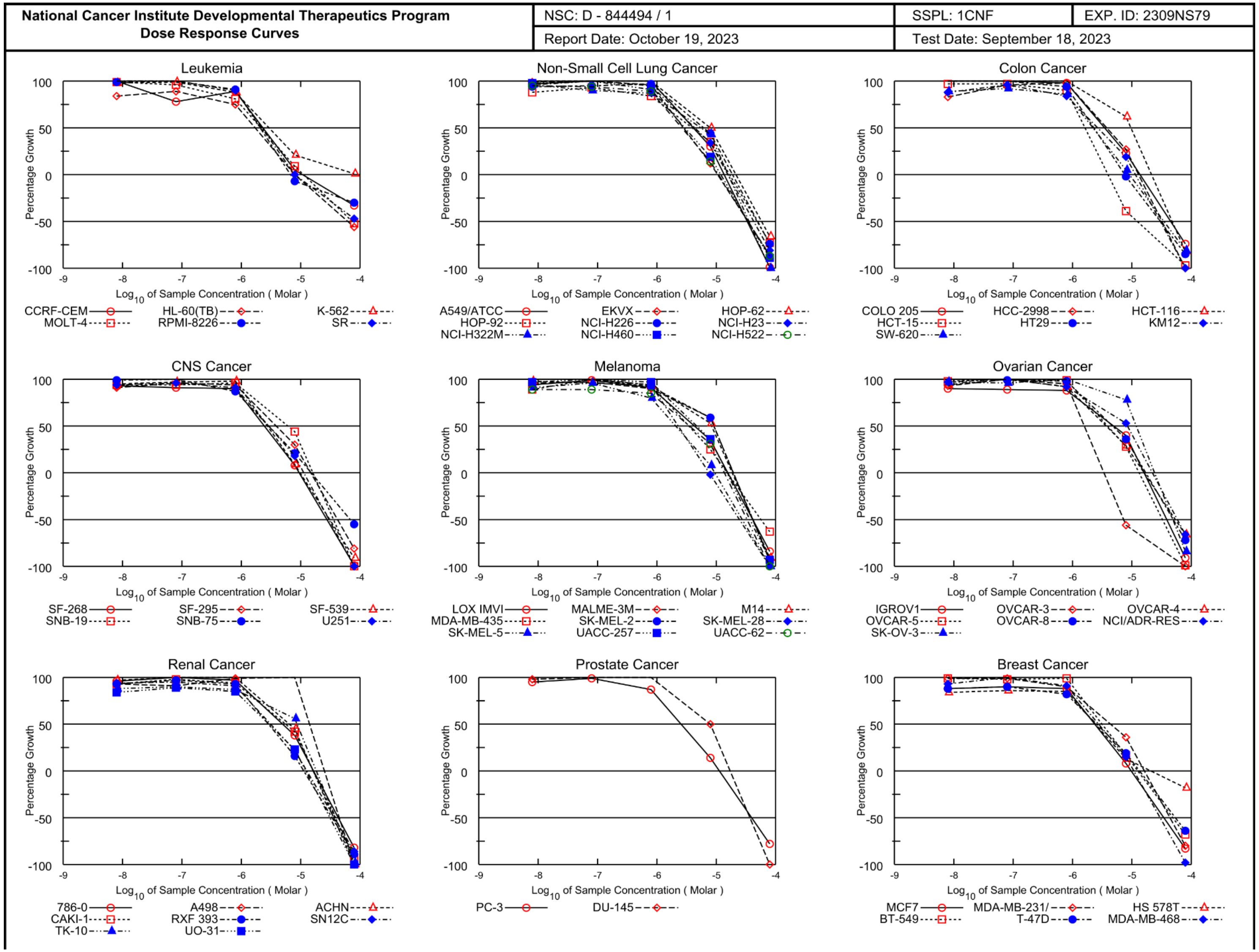

Fig. S41. NCI-60 five-dose screen, dose response curves by panel cancer cell lines for bulbillosin A (**1**).

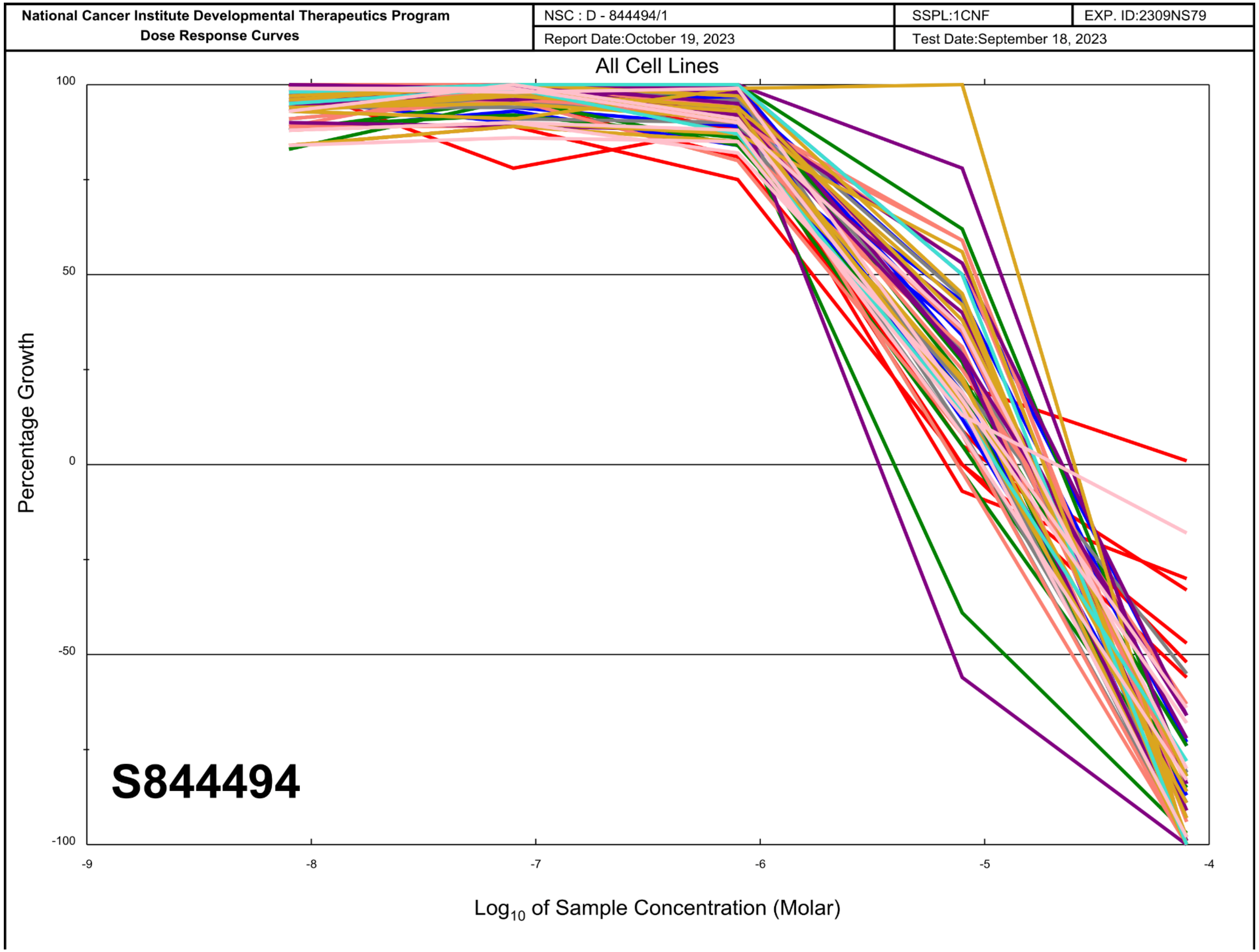

Fig. S42. NCI-60 five-dose screen, dose response curves all cancer cell lines for bulbillosin A (**1**).

References

Ahmed SA, Khan Z, Wang X, Moussa TAA, Al-Zahrani HS, Almaghrabi OA, Sutton DA, Ahmad S, Groenewald JZ, Alastruey-Izquierdo A, van Diepeningen A, Menken SBJ, Najafzadeh MJ, Crous PW, Cornely O, Hamprecht A, Vehreschild MJGT, Kindo AJ, Hoog GS de: (2016) Chaetomium-like fungi causing opportunistic infections in humans: a possible role for extremotolerance. Fungal Diversity 76:11–26. DOI: https://doi.org/10.1007/s13225-015-0338-5

Charria-Girón E, Stchigel AM, Čmoková A, Kolařík M, Surup F, Marin-Felix Y: (2023) *Amesia hispanica* sp. nov., Producer of the Antifungal Class of Antibiotics Dactylfungins. Journal of fungi (Basel, Switzerland) 9. DOI: https://doi.org/10.3390/jof9040463

Cheng Y, Schneider B, Riese U, Schubert B, Li Z, Hamburger M: (2006) (+)-*N*-Deoxymilitarinone A, a neuritogenic pyridone alkaloid from the insect pathogenic fungus *Paecilomyces farinosus*. Journal of Natural Products 69:436–438. DOI: https://doi.org/10.1021/np050418g

Hoog GS de, Ahmed SA, Najafzadeh MJ, Sutton DA, Keisari MS, Fahal AH, Eberhardt U, Verkleij GJ, Xin L, Stielow B, van de Sande WWJ: (2013) Phylogenetic findings suggest possible new habitat and routes of infection of human eumyctoma. PLoS Neglected Tropical Diseases 7:e2229. DOI: https://doi.org/10.1371/journal.pntd.0002229

Marin-Felix Y, Miller AN, Cano-Lira JF, Guarro J, García D, Stadler M, Huhndorf SM, Stchigel AM: (2020) Re-evaluation of the order Sordariales: Delimitation of Lasiosphaeriaceae s. str., and introduction of the new families Diplogelasinosporaceae, Naviculisporaceae, and Schizotheciaceae. Microorganisms 8. DOI: https://doi.org/10.3390/microorganisms8091430

Stahl M, Schopfer U, Frenking G, Hoffmann RW: (1996) Assignment of Relative Configuration to Acyclic Compounds Based on (13)C NMR Shifts. A Density Functional and Molecular Mechanics Study. The Journal of Organic Chemistry 61:8083–8088. DOI: https://doi.org/10.1021/jo960809n

van den Brink J, Facun K, Vries M de, Stielow JB: (2015) Thermophilic growth and enzymatic thermostability are polyphyletic traits within *Chaetomiaceae*. Fungal biology 119:1255–1266. DOI: https://doi.org/10.1016/j.funbio.2015.09.011

Vu D, Groenewald M, Vries M de, Gehrmann T, Stielow B, Eberhardt U, Al-Hatmi A, Groenewald JZ, Cardinali G, Houbraken J, Boekhout T, Crous PW, Robert V, Verkley GJM: (2019) Large-scale generation and analysis of filamentous fungal DNA barcodes boosts coverage for kingdom fungi and reveals thresholds for fungal species and higher taxon delimitation. Studies in Mycology 92:135–154. DOI: https://doi.org/10.1016/j.simyco.2018.05.001

Wang XW, Han PJ, Bai FY, Luo A, Bensch K, Meijer M, B K, Han DY, Sun BD, Crous PW, Houbraken J: (2022) Taxonomy, phylogeny and identification of *Chaetomiaceae* with emphasis on thermophilic species. Studies in Mycology 101:121–243. DOI: https://doi.org/10.3114/sim.2022.101.03

Wang XW, Houbraken J, Groenewald JZ, Meijer M, Andersen B, Nielsen KF, Crous PW, Samson RA: (2016a) Diversity and taxonomy of *Chaetomium* and chaetomium-like fungi from indoor environments. Studies in Mycology 84:145–224. DOI: https://doi.org/10.1016/j.simyco.2016.11.005

Wang XW, Lombard L, Groenewald JZ, Li J, Videira SIR, Samson RA, Liu XZ, Crous PW: (2016b) Phylogenetic reassessment of the *Chaetomium globosum* species complex. Persoonia 36:83–133. DOI: https://doi.org/10.3767/003158516X689657
